# A novel cystic fibrosis-mimetic *Pseudomonas* auxotrophic vaccine is protective *in vivo* and is associated with IL-17/IgA mucosal Immunity

**DOI:** 10.1016/j.ymthe.2026.06.029

**Published:** 2026-06-17

**Authors:** Youssouf Sereme, Bérengère Villeret, Ségolène Caboche, Samuel Ikeh, Hugo Leroy, Delphine Beury, Florence Maurier, Christophe Desterke, Maëlys Born-Bony, Zhou Xing, Romé Voulhoux, Jean-Michel Sallenave

**Affiliations:** 1 Institut National de la Santé et de la Recherche Médicale, U1149 (Centre de Recherche sur l’Inflammation), Université Paris-Cité, 16 Rue Henri Huchard, 75018 Paris, France; 2 Institut National de la Santé et de la Recherche Médicale, U1152 (Physiopathologie et Epidémiologie des Maladies Respiratoires), Université Paris-Cité, 16 Rue Henri Huchard, 75018 Paris, France; 3 Université de Lille, CNRS, Inserm, CHU Lille, Institut Pasteur de Lille, US41-UAR 2014, 59000 Lille, France; 4 University Paris Saclay, Faculty of Medicine, INSERM UMRS-1310, Villejuif, France; 5 McMaster Immunology Research Centre, McMaster University, Hamilton, ON, Canada; 6 Department of Medicine, McMaster University, Hamilton, ON, Canada; 7 Laboratoire de Chimie Bactérienne UMR7283, Centre National de la Recherche Scientifique, Aix-Marseille Université, Marseille, France

**Keywords:** mucosal vaccination, LasB, ASM, *Pseudomonas aeruginosa*, cystic fibrosis, IgA, IL-17, bacteriophage Pf4, Pf6

## Abstract

*Pseudomonas aeruginosa* (P.a) is a Gram-negative opportunistic pathogen that poses a major global health threat, particularly in immunocompromised individuals, patients with cystic fibrosis, and those with burn injuries or ventilator-associated pneumonia. Despite intense efforts, no vaccine is available for human use. In this context, live attenuated vaccines represent a promising but underexplored approach, offering the potential to elicit robust, long-lasting, and multifaceted immune responses including inducing trained immunity. Here, we sub-cultured *ΔLasB*-PAO1 (a P.a strain we have previously shown to have reduced virulence) in artificial sputum medium, a culture medium mimicking cystic fibrosis (CF) sputum in which bacteria often show auxotrophy. We showed that such strain (“V” for vaccine) was auxotrophic, less virulent, and had characteristics of “CF-like strains.” Crucially, “V” induced both local (IgA) and systemic humoral responses as well as memory Th17 immune responses, and could, when administered in the lung, but not intra-muscularly, protect mice against a lethal PAO1-WT infection. Overall, the present study demonstrates that our vaccine formulation, in addition to providing an advantageous auxotrophic phenotype adapted to the CF setting, was efficient, when given mucosally and was associated with a pathway involving Th17 responses and secretory IgA, a critical barrier that neutralizes pathogens before tissue invasion.

## Graphical abstract

**Figure:**
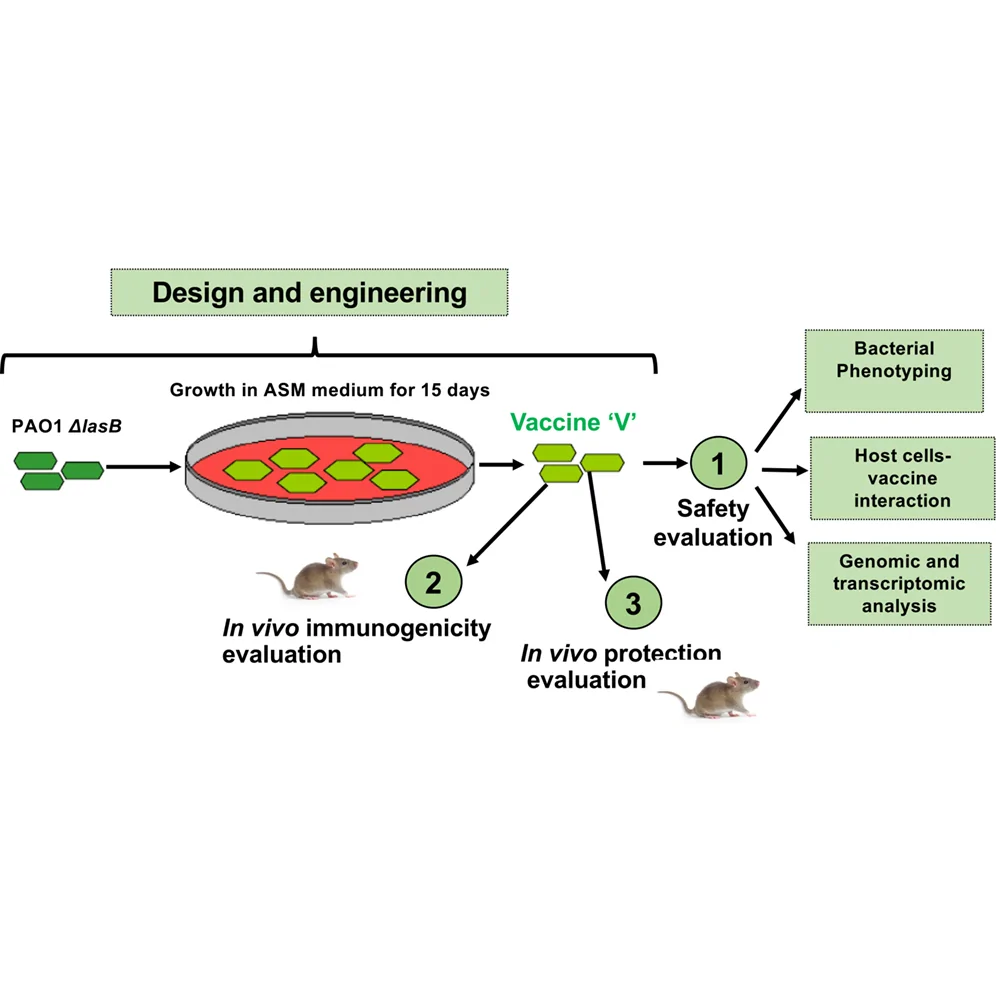

## Teaser

Sallenave and colleagues showed that a novel cystic fibrosis mimetic, attenuated, and auxotrophic *Pseudomonas aeruginosa* strain (“V”) demonstrates *in vivo* safety while eliciting durable humoral and cellular immunity in mice. Mucosal, but not intramuscular, vaccination confers protection against lethal challenge, highlighting the critical role of administration in effective vaccine-induced protection.

## Introduction

*Pseudomonas aeruginosa* (P.a) is a Gram-negative opportunistic pathogen that poses a major global health threat, in immunocompromised individuals, patients with cystic fibrosis (CF), and those with burn injuries or ventilator-associated pneumonia, and it has been classified as a critical priority pathogen.^1^ Despite therapeutic advances in CF,^2^^,^^3^ the rise of multidrug-resistant (MDR) and extensively drug-resistant (XDR) strains requires more effective preventive strategies. Despite this, no vaccine is currently available for human use. Although various platforms—including subunit, conjugate, and outer membrane protein-based vaccines—have been explored, most candidates have failed to provide broad, durable protection in clinical trials.^4^^,^^5^^,^^6^^,^^7^ In this context, live attenuated vaccines (LAVs) represent a promising underexplored approach, to elicit robust, long-lasting, and multifaceted immune responses including trained innate immunity.^4^^,^^5^^,^^6^^,^^7^^,^^8^ These include strong humoral and cellular (CD4^+^/CD8^+^ T cell) immunity—which closely parallels natural exposure.^9^ Additionally, certain live vaccines (e.g., oral polio, rotavirus) administered mucosally stimulate localized IgA and mucosal immunity, which can reduce transmission—a benefit rarely seen with inactivated or subunit formulations.^10^ Their replicative nature enables dose sparing, reducing antigen requirements and facilitating cost-effective mass production and distribution.^11^

However, traditional LAVs carry inherent safety risks. In immunocompromised patients, even attenuated strains may revert to a virulent form or cause unintended systemic infection. Moreover, LAVs require careful dose control and rigorous safety testing to minimize the risk of persistent colonization or dissemination, which could be dangerous in hospital or CF settings. Despite these risks, LAVs remain attractive because they stimulate both systemic and mucosal immunity, producing long-lasting protective responses.^4^^,^^5^^,^^6^^,^^7^ It was shown that by engineering auxotrophic bacterial strains that lack aromatic amino acids (*ΔaroA*) or D-glutamate, such LAVs were replication-impaired outside of a controlled environment, while retaining immunogenic components and being incapable of causing disease.^12^^,^^13^^,^^14^^,^^15^^,^^16^ Another interesting approach consisted of using an unnatural amino acid, creating a conditional *Pseudomonas* vaccine preventing bacterial infection.^17^

Here, we develop, for the first time to our knowledge, an auxotrophic P.a strain phenotypically as close as possible to clinical strains present in sputa secretions from patients with CF (pwCF), with reduced virulence while being robustly immunogenic. This was done by sub-culturing a genetically modified P.a (*ΔLasB*-PAO1) in artificial sputum medium (ASM), a culture medium mimicking CF sputum.^18^^,^^19^^,^^20^^,^^21^^,^^22^^,^^23^^,^^24^^,^^25^^,^^26^

We show by RNA-seq and whole genome sequencing (WGS) that it was indeed auxotrophic, less virulent, had characteristics of “small colony” CF-like strain, and secreted high amounts of filamentous phages (mainly Pf4). Crucially, it was able to induce both local (IgA) and systemic humoral responses as well as memory Th17 immune responses, and it could, when administered via the respiratory mucosal (but not intramuscular) route, protect mice against a lethal PAO1 infection.

## Results

### Genetic profiling of PAO1 strains cultured in LB or ASM medium

#### RNA-seq and whole genome sequencing of PAO1 strains cultured in LB or ASM medium

**Figure 1:**
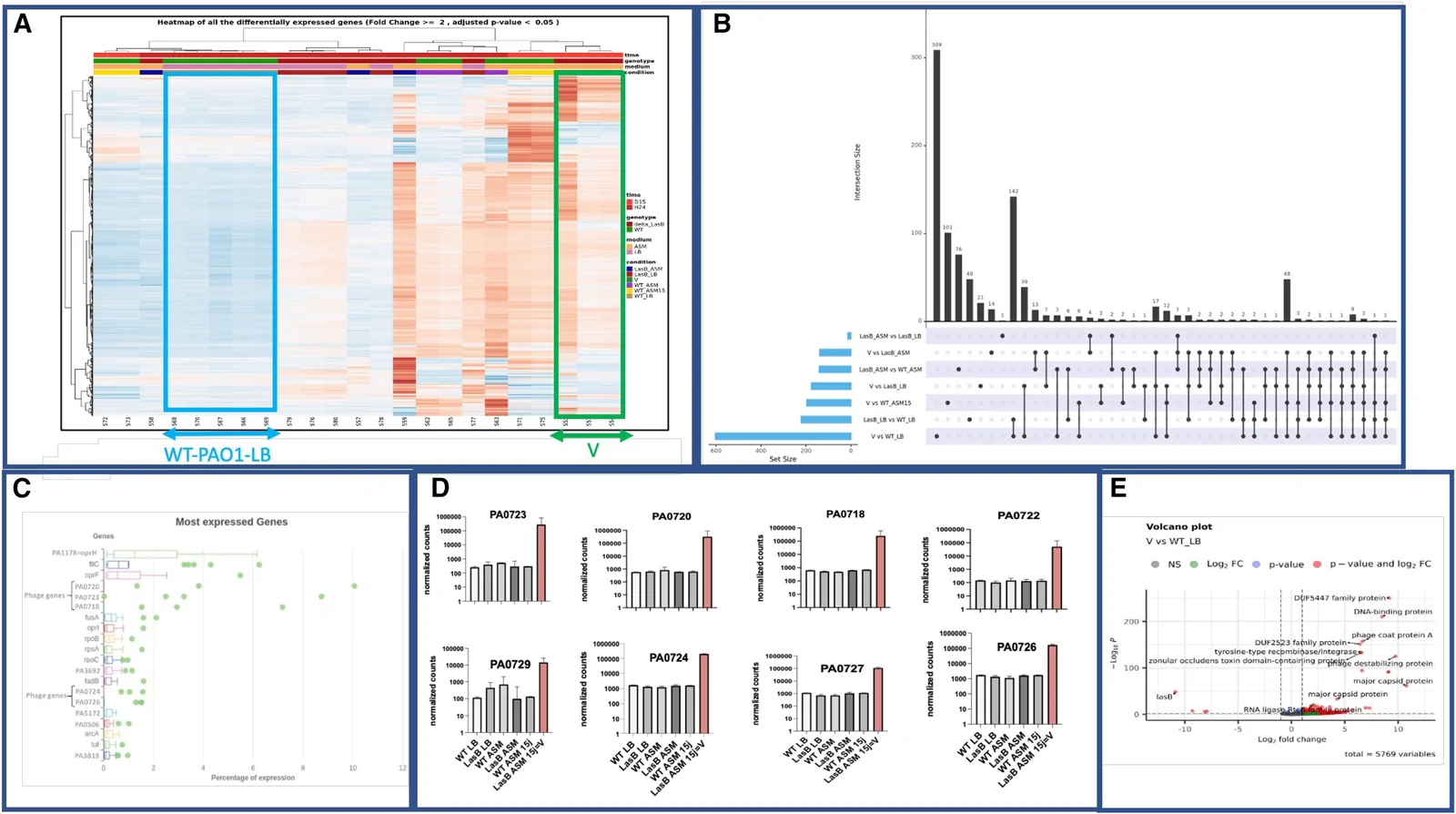
RNA-seq analysis (A) Heatmap of differentially expressed genes (fold change > 2, adjusted *p* < 0.05). Heatmap representing all genes significantly differentially expressed between experimental conditions: PAO1-WT-LB-24hrs, PAO1-WT-ASM-24hrs, PAO1-WT-ASM-15days, *ΔLasB*-PAO1-LB-24hrs, *ΔLasB*-PAO1-ASM-24hrs, *ΔLasB*-PAO1-ASM-15days = “V”: (fold change >2, adjusted *p* value <0.05). Expression values are shown as log_2_-transformed, z score-normalized counts across samples. By applying row scaling, each row corresponds to a gene and each column to a sample. Colors indicate relative expression levels, with red representing higher and blue lower expression compared with the mean. Hierarchical clustering was applied to both genes and samples to highlight expression pattern similarities. (B) UpSet plot of intersecting differentially expressed genes across contrasts (see A). The plot displays the top 50 intersection sets, corresponding to genes exhibiting significant fold change variations (adjusted *p* value <0.05, |fold change| > 2). Vertical bars represent the number of genes shared among specific combinations of contrasts, while horizontal bars indicate the total number of differentially expressed genes within each contrast. This visualization highlights the degree of overlap and specificity of transcriptional responses across experimental conditions. (C) Top 20 most highly expressed genes across all samples (see A). Displayed are the 20 genes with the highest expression levels across all RNA-seq samples, irrespective of experimental conditions. Expression is represented as a percentage of total transcriptomic reads per sample. (D) Top Pf4 bacteriophage normalized RNA counts are shown for the core region genes (see genomic diagram in Figure S1A). (E) Volcano plot showing differential gene expression between “V” and PAO1-WT-LB-24hrs. Volcano plot of all 5,769 genes showing log_2_ fold change versus –log_10_ adjusted *p* value, with genes significantly upregulated in “V” relative to PAO1-WT-LB highlighted in red (adjusted *p* ≤ 0.05, |log_2_ fold change| ≥ 1).

To genetically characterize the P.a vaccine, we first analyzed by bulk RNA-seq 6 different conditions of culture for PAO1-WT or *ΔLasB*-PAO1, varying the time of culture or the medium used (PAO1-WT-LB-24hrs, PAO1-WT-ASM-24hrs, PAO1-WT-ASM-15days, *ΔLasB*-PAO1-LB-24hrs, *ΔLasB*-PAO1-ASM-24hrs, *ΔLasB*-PAO1-ASM-15days = “V”), ASM being a culture medium mimicking CF sputum,^20^^,^^21^^,^^22^^,^^23^^,^^24^^,^^25^^,^^26^ secretions in which bacteria often show auxotrophy.^26^^,^^27^^,^^28^^,^^29^ A heatmap (Figure 1A), with each column representing a cultured sample, highlighted the distinct profile of the “V” condition (green rectangle), which exhibited a global upregulation of numerous genes relative to the PAO1-WT-LB control (blue rectangle). Indeed, the UpSet plot representation showed that the contrast “V” versus PAO1-WT-LB (24hrs) had most differentially expressed genes (set size = 599), and among these, most (*n* = 309) only belonged to that contrast (Figure 1B). Regardless, we showed that among the 20 most highly expressed genes across all samples (all 6 conditions/groups analyzed together) (Figure 1C) were *oprH*, *fliC*, *oprF*, and *oprI*, all genes already tested as subunit vaccines against P.a^4^ (Figure 1C). In addition, notably, 5 genes were Pf4 bacteriophage genes (*PA0720*, *PA0723*, *PA0718*, *PA0724*, and *PA0726*)^30^^,^^31^ and P.a. Pf prophages are known to play important roles in virulence and CF biofilm formation.^32^^,^^33^ The 12.4-kb Pf4 genome comprises 20 genes (*PA0715*–*PA0729*; Figure S1A), 12 of which form the core region shared with the Pf6 prophage. Both Pf4 and Pf6 are co-produced in PAO1 biofilms, where they contribute to host antibiotic resistance and virulence.^34^ Intrigued by this finding, we analyzed these 5 Pf4 genes, as well as other Pf4 genes in the 6 groups and showed that the transcription levels of the Pf4 genes belonging to the core region, including *PA0718*, *PA0720*, *PA0723*/*gVIII*, *PA0724*, and *PA0726* were dramatically increased in the “V” strain compared with the other groups (Figures 1D and S1B, Log2FC values between −7 and −11). By contrast, the expression of three genes (*PA015*, *PAO16*, *PAO16.1*/*pf4r*) were downregulated in the “V” strain (Log2FC values of values of +9, +8, and +8, respectively (Figure S1B red oval), compared to the PAO1-WT-LB (24hrs) strain and to the other groups (not shown). Interestingly, Guo et al.^34^ recently reported that superinfective (SI) Pf4 prophages released during CF biofilm growth undergo an approximate 27% reduction in genome size, corresponding to the so-called 3.3-Kb discarded region (DR) containing the three genes *PA015*, *PAO16*, and *PAO16.1/pf4r* (Figure S1A). By comparison, most genes from the Pf6 prophage did not show global overexpression in the “V” strain, compared with the PAO1-WT-LB (24 h) strain (Figure S1C). Among some exceptions, however, three Pf6-specific genes—*pfkA*, *pfkB*, and *pfkC*—were overexpressed in the “V” strain, with Log_2_FC values of −3.5, −5, and −5, respectively (Figure S1C, red oval). Interestingly, as previously reported,^34^ Pf6 *pfkA*, *pfkB*, and *pfkC* genes form a regulatory module that controls virion production of the co-resident Pf4 prophage. Altogether, this prophage transcriptomics analysis showing that Pf4 core genes (and the excisionase *xisf4*) were strongly upregulated and that genes *PA015*, *PAO16*, and *PAO16.1* (present in the DR region) were downregulated in the “V” strain suggested that culturing it during 15 days in ASM medium may have downregulated (or deleted) the genes present in the DR region of the Pf4 prophage DNA. To determine whether the DR genes were “simply” downregulated at the transcriptional level or whether that portion of phage DNA was indeed missing in the Pf4 genome, as evident in some P.a strains present in biofilms,^31^^,^^34^ WGS was then performed in PAO1-WT-LB-24hrs (henceforth named “PAO1-WT”) and in *ΔLasB*-PAO1-ASM-15d (“V” strain), 2 groups shown to be well differentially clustered (blue and green rectangles, Figure 1A).

Assembly and manual inspection of sequencing data obtained with Oxford nanopore technology led to a complete circular genomic sequence of 6,315,638 bp for the PAO1-WT strain (JBRXYX000000000) and 6,310,806 bp for the “V” strain (JBRXZF000000000).

Comparing the two genomic sequences displayed minimal differences, except obviously the absence of the *LasB* gene in the “V” strain and only a single nucleotide variant, located in an intergenic region. Nevertheless, a notable difference was observed in the Pf4 prophage sequence: the complete 12.4-kb Pf4 genome was detected only in the PAO1-WT strain, whereas the “V” carried only the 9.1-kb form, lacking the 3.3-kb region. Furthermore, we showed that this genotype was maintained over at least 57 passages. Indeed, we showed by qPCR that the Pf4r gene, present in the DR (Figure S1A), was virtually absent in the V strain, compared with the PAO1 strain (and that of several CF clinical strains, Figure S1D), in keeping with the fact that V consisted mainly of the secreted SI 9.1-kb form region. Conversely, the circularized form of the prophage (presence of the attP site), a marker of replication and secretion of Pf4, was present in much higher copies in V, compared with the other strains (Figure S1E).

Altogether, the WGS and qPCR analysis clearly confirmed the “prophage transcriptomics” data presented above, demonstrating that indeed, an SI 9.1-kb secreted form, lacking the 3.3-kb DR region of the full-size 12.4-kb Pf4,^31^^,^^34^ was generated in the “V” strain. Furthermore, when comparing the “complete” PAO1 RNA-seq data (i.e., not restricted to prophage genes), from PAO1-WT and “V,” it was striking that overall the top 12 mostly expressed genes in “V” (relative to PAO1-WT) were Pf4 genes and belonged to the Pf4 core region (Figure 1E; Table S1).

In addition to these phage genes, KEGG Pathway Enrichment analysis showed an induction of specific pathways in the “V” strain, i.e., “Biosynthesis of secondary metabolites,” “Ribosomes,” “Carbon metabolism,” “Biosynthesis of amino acids,” “Bacterial chemotaxis,” “Flagellar assembly,” “Other carbon fixation pathways, “Citrate cycle,” “Biosynthesis of various nucleotide sugars,” “Oxidative phosphorylation,” “Phosphonate and phosphinate metabolism,” “Xylene degradation,” and “Fluorobenzoate degradation” (Figure S2). These pathways intersect at multiple levels, particularly in energy metabolism, redox reaction, cofactor biosynthesis, biosynthetic precursors, stress response, and virulence regulation in bacteria, closely mirroring the nutrient-restricted and redox-challenged environment of CF. Indeed, the upregulation of secondary metabolite biosynthesis suggests that “V” is shifting metabolic resources from virulence toward a persistence mode of community behavior and survival (slow growth, persistence). Relatedly, the upregulation of the node “Biosynthesis of nucleotide sugars” suggests the generation of the “building blocks” used by glycosyltransferases to assemble a wide variety of extracellular polysaccharides, including exopolysaccharides and alginate, involved in biofilm matrix generation and structural stability (e.g., UTP-glucose-phosphate uridyltransferase GalU, alpha-1,3-rhamnosyltransferase, glycosyltransferase family 4 proteins) (Table S1). This is confirmed by the upregulation of genes involved in alginate biosynthesis regulators (e.g., *AlgR*, *MucA*, *MucB*, *MucC*, *AlgP*, *AlgZ)*, stress response σ factors (*RpoE*, *RphoH*, *SigX*, *AlgU*), and stress response and DNA repair (e.g., *RecA*, *LexA*, *OspR*, *KatA*, *AhpC*, *DsbA*, *peroxiredoxin*, *glutaredoxin*; Table S1). Also, iron metabolism genes (siderophore-like or redox proteins) were upregulated in “V” (bacterioferritin, bacterioferritin-associated ferredoxin, Bacteriohemerythrin, azurin, ferric iron uptake transcriptional regulator, *IscR*, *ErpA*, succinate dehydrogenase, cytochrome *b*556 subunit, cytochrome *c* oxidase subunits *CcoM/CcoQ-FixQ*). Interestingly, potentially feeding into that “stressed” phenotype, the upregulated KEGG nodes phosphonate and phosphinate metabolism, xylene degradation, and fluorobenzoate degradation, together with central nodes like carbon metabolism, the citrate cycle, and biosynthesis of amino acids, suggest a bacterial metabolic adjustment toward nutrient flexibility and survival in a “stressful” environment.

Together, these transcriptional and pathway changes suggest that strain “V” undergoes a broad metabolic and stress-adaptive reprogramming consistent with a transition toward persistence and survival under cystic-fibrosis-like environmental pressures.^18^^,^^19^^,^^20^^,^^21^^,^^22^^,^^23^^,^^24^^,^^25^^,^^26^^,^^35^

### Δ*LasB-*PAO1 “V” strain cultured in ASM medium is auxotrophic and less virulent, with “small colony variant” characteristics

**Figure 2:**
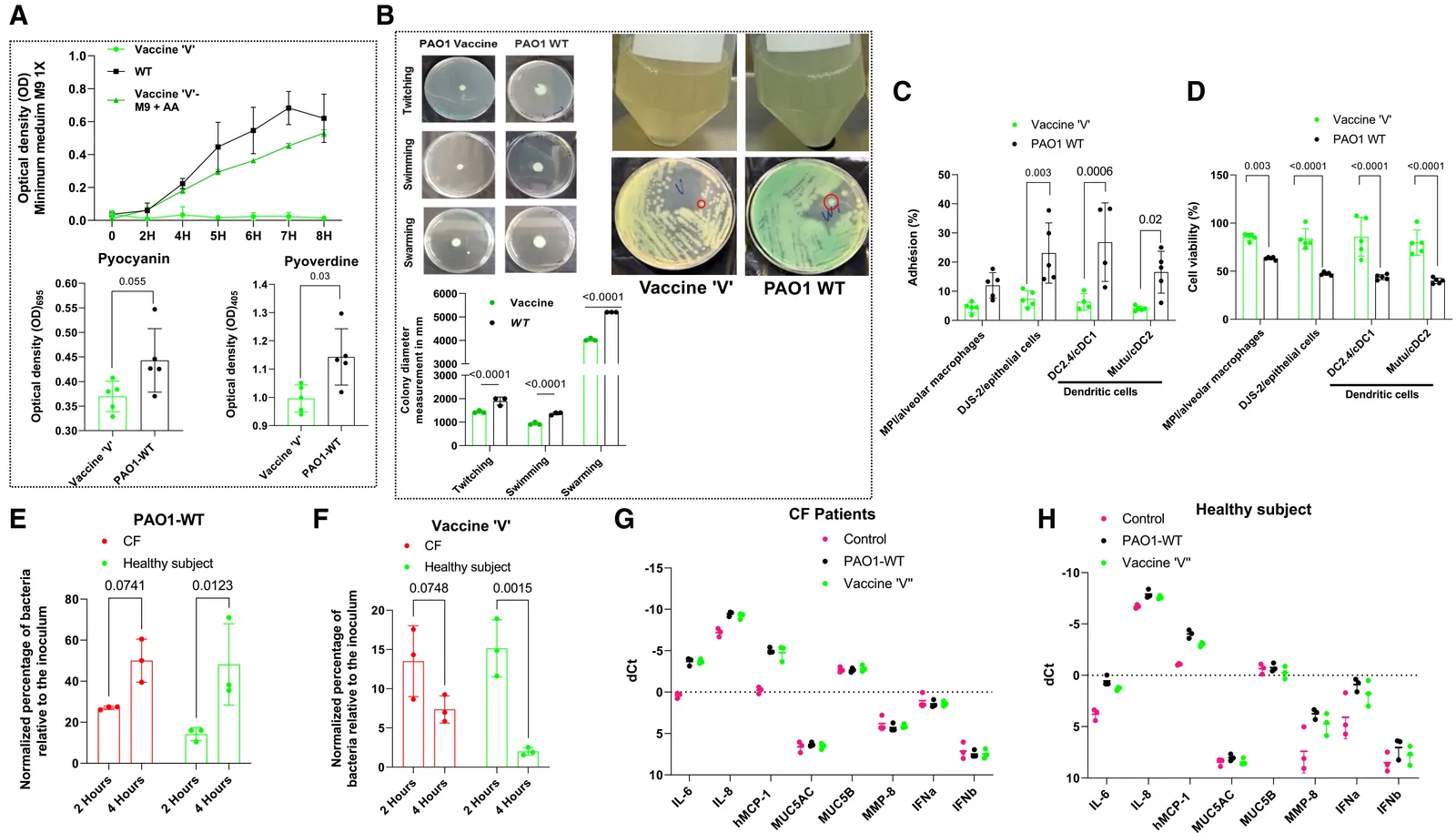
Evaluation of the virulence of the PAO1 vaccine strain “V” (A) (1) Growth curves for P.a PAO1-WT-LB and the vaccine strain “V” in M9 minimal medium and M9 supplemented with amino acids (AA). (2) Pyocyanin and pyoverdin secretion in PAO1 WT and “V.” (B) measurement of motility twitching, swimming, and swarming (*n* = 3) of the vaccine strain “V,” compared with the PAO1-WT-LB strain (*p* < 0.05), and phenotypic appearance of colonies. Colony diameter measurement is presented as mean SD values. (C) Percentage of adhesion of PAO1-WT or “V” to alveolar macrophages (MPI cell line, *n* = 5), to lung club epithelial (DJS-2 cell line, *n* = 5), and to dendritic cells DC2.4/cDC1 (*n* = 5) and Mutu/cDC2 (*n* = 5). Two-way ANOVA with Tukey’s multiple comparisons tests were used to determine statistical significance (*p* < 0.05). (D) Cell viability of MPI (*n* = 5), DJS-2 (*n* = 5), DC2.4 (*n* = 5), and Mutu/cDC2 (*n* = 5) was measured post infection with the bacterial strains (two-way ANOVA with Tukey’s multiple comparisons, *p* < 0.05). (E and F) Primary bronchial epithelial cells from a healthy (H) or CF individual (CF) (Mucilair, Epithelix) were infected at a MOI of 1 with either PAO1-WT or V over 2 and 4 h. Bacterial load was then assessed by pooling supernatants and cell lysates. (G and H) An aliquot of cell lysates was used for RNA preparation, and cytokine/mediator levels were expressed as dCt/HPRT. NB: the lower the dCT, the higher the expression.

To examine the biologic property of the P.a vaccine (Δ*LasB-PAO1* “V”), we cultured the “V” strain (generated in ASM medium during a 15-day culture, as explained above) in the minimal medium M9 and found that only adding specific amino acids was able to restore its growth to a level comparable to that of PAO1-WT (Figure 2A), indicating an auxotrophic phenotype. In addition to auxotrophy, the phenotypic shape of the “V” colonies and their mobility behavior (twitching, swimming, swarming) were also akin to that of the small colony variants (scv) typically found in CF secretions^36^^,^^37^ (Figures 2B and S3) that we also found in some of our clinical strains (Figure S3C). Importantly, the persistence of that phenotypic change was checked on multiple occasions, over more than 50 passages, and an example of a 7-day time course of sub-passaging PAO1 WT and V is shown in (Figure S3A). This shows, remarkably, a difference in pigmentation/color (from “greenish” to “yellowish”) between the PAO1-WT and “V” strain, which may be caused by several factors, including partial bacterial lysis caused by prophage induction (see above), changes in metabolism including that of phenazines/pyocynins, pyoverdins, and flavin-related enzymes (6,7-dimethyl-8-ribityllumazine synthase, NADPH-dependent FMN reductase, ferredoxin FdxA, ferredoxin-NADP+ reductase, NO-inducible flavohemoprotein, FAD-binding oxidoreductase, quinolones; see Table S1). Indeed, we showed that pyoverdine and pyocyanin, two virulence factors giving a typical blue/greenish color to P.a secretomes, were downregulated in “V” (Figure 2A).

Functionally, the potential decreased virulence of “V” was demonstrated by its diminished capacity to adhere to a variety of eukaryotic cell lines (alveolar macrophages, epithelial cells, and dendritic cells; Figure 2C), and by its lesser cytotoxic properties on these cells (Figure 2D). We also compared the differential persistence of “V” and of PAO1-WT in primary bronchial epithelial cells from a healthy individual and from a CF patient and showed, while PAO1 was increasing in the 0- to 4-h period, V was, in contrast, cleared faster (Figures 2E and 2F). Also, by measuring the expression of a variety of inflammatory mediators (panels G and H), we showed, expectedly, that it was generally higher in CF cells, compared with “healthy/cells” (lower dCTs meaning higher expression), irrespective of whether cells were unstimulated (“Control”) or infected with PAO1-WT or with V. In addition, although both strains were equally “inflammatory” in CF cells (panel G), probably reflecting a saturation effect in the latter, there was a trend, although not statistically significant, for a reduction, in healthy cells, in the levels of certain cytokines in V- versus PAO1 WT-infected conditions (panel H).

Crucially, we also demonstrated *in vivo* (Figure S4A), using an infectious dose (5.10^7^ cfu) 50× higher than that used for our immunization protocols (10^6^ cfu, see below), that compared with PAO1-WT, V bacterial load was 2–3 logs lower in bronchoalveolar lavages (BALs), lungs, and spleens, 24 h post infection (panel B). Furthermore, in keeping with the latter, the cytokine secretion was reduced in BALs and spleen extracts of V-infected animals (panels C-D).

In addition, using higher doses of bacteria, we performed dose-response infections and showed that, in accordance with the above data, V was much less virulent *in vivo* (100% and 0% mortality for 5/6.5.10^8^ cfu of PAO1 WT and V, respectively; panels E-F).

Altogether, the above *in vitro* and *in vivo* observations suggested the “V” strain to bear similarities with those adapted to “CF-live environments,” being auxotrophic and less virulent than the PAO1 strain, thus supporting its utilization for immunization.^18^^,^^19^^,^^20^^,^^21^^,^^22^^,^^23^^,^^24^^,^^25^^,^^26^^,^^35^

### Respiratory mucosal, but not intramuscular, immunization with Δ*LasB-PAO1* “V” strain induces robust mucosal immune responses

**Figure 3:**
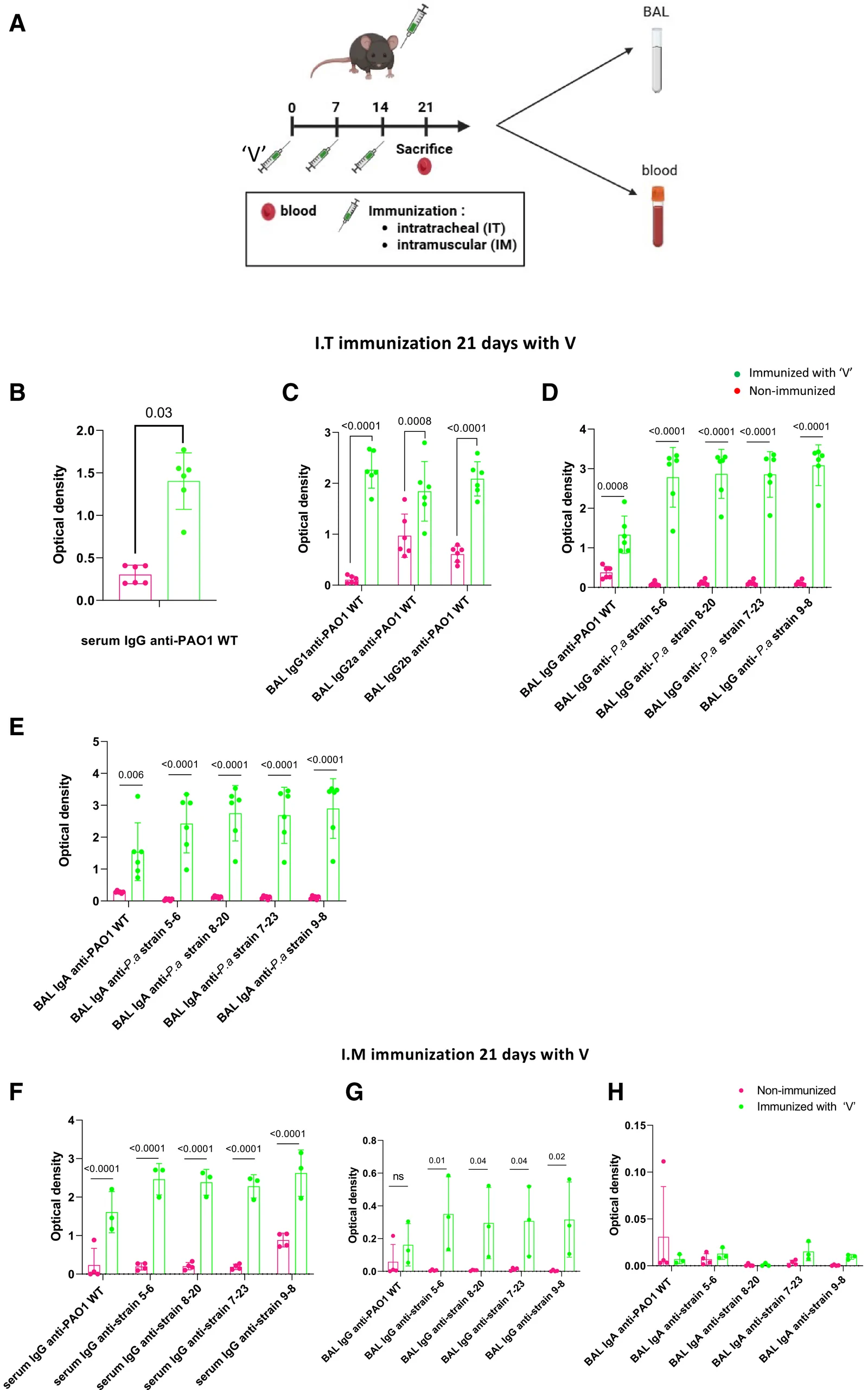
Assessment of the immunogenicity of the “V” strain: Humoral response (A) Immunization protocol: at days 0, 7, and 14, C57BL/6 male mice were given (IT [*n* = 6] or IM [*n* = 3]) either PBS (control) or were immunized with 10^6^ CFU of the vaccine “V” strain. At day 21, 7 days after the last boost, BALs and blood were collected. (B and F) Measurement of anti-PAO1-WT, anti-P.a 5–6, 8–20 (clinical mucoid strains), and anti-P.a 7–23, 9–8 (clinical non-mucoid) serum IgG levels. (C) Measurement of anti-PAO1-WT IgG1, Ig2a, and IgG2b BAL levels. (D and G) Measurement of anti-PAO1-WT, anti-P.a 5–6, 8–20, 7–23, 9–8 BAL IgG levels. (E and H) Measurement of anti-PAO1-WT, anti-P.a 5–6, 8–20, 7–23, 9–8 BAL IgA levels. Statistics: Mann-Whitney non-parametric test for (A), and multiple *t* tests were used for (B–G), and error bars represent standard deviation (SD).

We then assessed the immunogenicity of the “V” strain in C57/Bl6 mice following vaccination via either a lung mucosal oro-pharyngeal route (referred indiscriminately to as “intra-tracheal” [i.t.]) or an intramuscular (i.m.) route. Thus, the mice were vaccinated at D0, D7, 14, and were sacrificed at D21 (Figure 3A). We showed that at day 21 (1 week after the last boost), while both routes induced robust serum anti-PAO1 IgG production (Figures 3B and 3F), only i.t. immunization elicited high levels of specific anti-PAO1-IgG and -IgA in BALs (Figures 3D and 3E), when compared with i.m. vaccination (Figures 3G and 3H). In BALs, all the tested isotypes (IgG1, IgG2a, and IgG2b) were equally induced following i.t. immunization (Figure 3C).

Importantly, BAL IgG and IgA recognized both PAO1-WT and unrelated mucoid and non-mucoid clinical strains (respectively labeled 5–6; 8–20 and 7–23; 9–8; Figures 3D and 3E), and serum IgGs had the same cross-reactive properties (Figure 3F).

**Figure 4:**
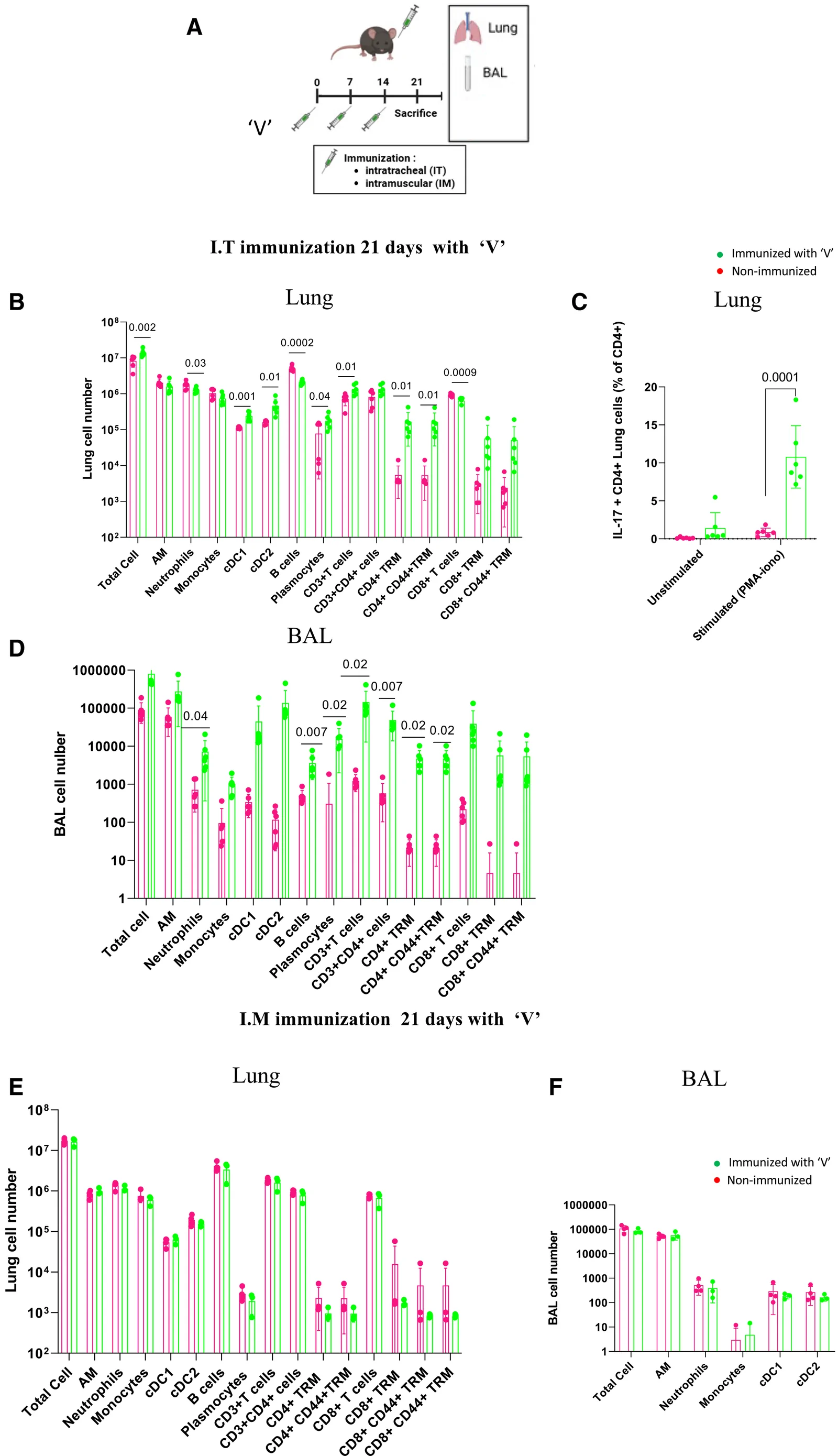
Flow cytometry assessment of the immunogenicity of the “V” strain: Cellular response (A) Immunization protocol as in Figure 3. Counts of total cells, AMs, neutrophils, monocytes, dendritic cells (cDC1 and cDC2), B lymphocytes, plasma cells, CD3+ T cells, CD3+CD4+ T cells, CD4+CD44+TRM, CD8, CD8+TRM, and CD8+CD44+TRM T cells were assessed by flow cytometry in lungs (B and E) and BALs (D and F). (C) Cells were stimulated with PMA-ionomycin and percent of IL-17+CD4+ cells was assessed. NB: TRMs (tissue-resident memory cells) are defined here as CD3+CD69+CD103+. The flow cytometry strategies are depicted in Figures S5, S6, S7, and S8. Multiple *t* tests were used to determine significance, *p* < 0.05. Error bars represent standard deviation (SD).

When cellular responses were assessed by flow cytometry at the same time point (Figure 4A, see Figures S5, S6, S7, and S8 for flow cytometry strategy) mucosal immunization with “V” was again more efficient at inducing immune responses in both lung tissues (Figure 4B) and BAL compartments (Figure 4D). Notably the numbers of neutrophils, dendritic cells (both cDC1 and cDC2), B cells, plasmocytes, and CD4 T cells (both classical and tissue-resident memory [TRM]) as well as those of CD8+ T cells were increased in lungs. Importantly, the proportion of IL-17+ cells among CD4+ T cells was increased in “V”-immunized animals after PMA/ionomycin stimulation (Figure 4C).

By comparison, none of the lung and BAL cell populations were increased in i.m-immunized animals (versus non-immunized animals) (Figures 4E and 4F).

After fully depleting CD4 T cells in blood, spleen, and lungs in PBS/V-immunized animals (Figure S9 panels B1-B2, E1-E2, F1-F2, H1-H2, I) and assessing for CD45 intra-vascular labeling (Figure S9 panels A2, B2, C2, D2, E2, F2, G2, H2), we showed that TRMs (initially present in the lungs of isotype-treated vaccinated mice, panel D1) were also efficiently removed (panel F1). Despite this, interestingly, intra-nasally (i.n) V-vaccinated CD4 T-depleted mice were equally protected against a lethal PAO1 challenge (panel J).

Then, by comparing the mucosal immunogenicity of the “V” strain to that of other PAO1 strains, i.e., PAO1-WT, *ΔLasB*-PAO1, and *ΔLasB*-PAO1 HI (heat-inactivated) (all strains cultured in LB), we showed that the specific anti-PAO1 antibody responses in serum and BALs were enhanced when the “V” strain was considered (Figure S10). When the cellular immune responses were assessed by flow cytometry, they were globally found to be equivalent, as assessed in BALs (Figure S11) and lungs (Figure S12), again demonstrating that the reduced virulence of the “V” strain (Figures 2 and S4) was not affecting its immune-stimulating potential (and was even beneficial in terms of antibody responses), compared with other PAO1 strains.

**Figure 5:**
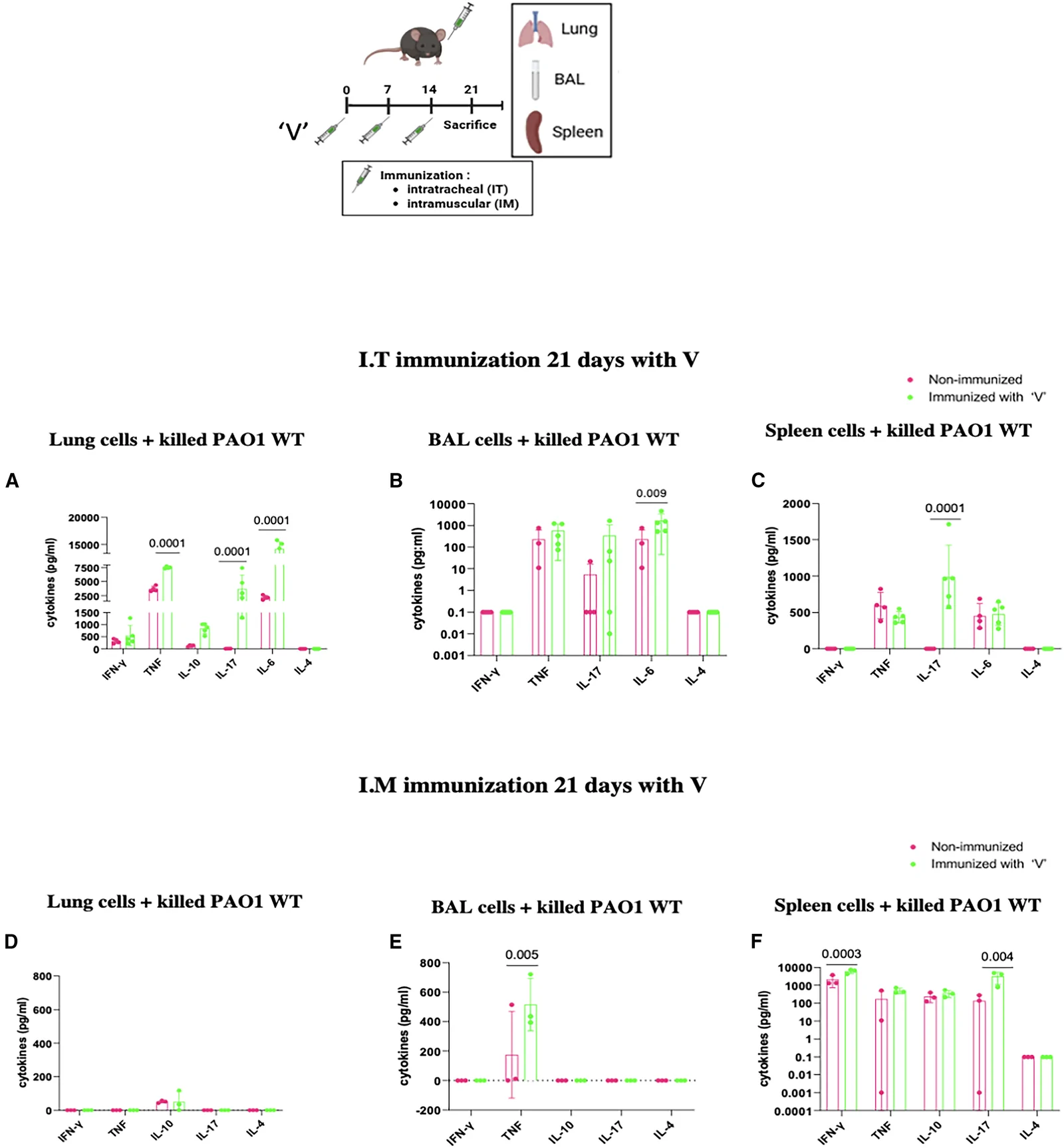
Cytokine readout after *ex vivo* cell stimulation with killed PAO1-WT After i.t. or i.m. immunization (see Figures 3 and 4), lung (A and D), spleen (C and F), and BAL (B and E) were harvested, and cells were incubated (10^6^, 10^6^, 20,000 cells, respectively) with gentamycin-killed bacteria (MOI = 1) during 48 h. IFN-g, TNF, IL-10, IL17, IL-6, and IL-4 cytokines levels were then measured (ELISA) in cell supernatants. Two-way ANOVA comparison tests were used for statistical analysis (*p* < 0.05). Error bars represent standard deviation (SD).

We therefore focused the rest of our study on the “V” strain and further showed by performing *ex vivo* tissue antigenic recall experiments (with killed PAO1-WT) that mucosal i.t. immunization was far superior at inducing a lung Th17 response (as measured by IL-17 levels) when compared with the i.m. vaccination protocol (Figures 5A and 5D). Interestingly, splenic responses were equivalent in terms of IL-17 recall responses, irrespective of the route of immunization (Figures 5C and 5F), and while spleen IFN-g levels were increased post i.m. immunization (Figure 5F), no splenic IFN-g was detectable post i.t. immunization (Figure 5C). When BAL cell antigenic recall was considered, IL-17 secretion was detectable in i.t.-immunized mice (Figure 5B) but not after i.m. vaccination (Figure 5E). IL-17 was likely produced by T cells, which accounted for roughly 20% of total cells in immunized mice (Figure 4D). On the other hand, BAL TNF (Figures 5B and 5E) was likely produced mainly by innate immune cells since these cells account for 95% of total cells in non-immunized mice (Figure 4D) and since the levels of this cytokine are roughly comparable in non-immunized and i.t. immunized mice (in the latter, resident AMs and dendritic cells account for to 40% and 20% of total cells, respectively, Figure 4D).

### Respiratory mucosal, but not intramuscular, immunization with Δ*LasB-*PAO1 “V” confers potent immune protection against a lethal PAO1-WT challenge

**Figure 6:**
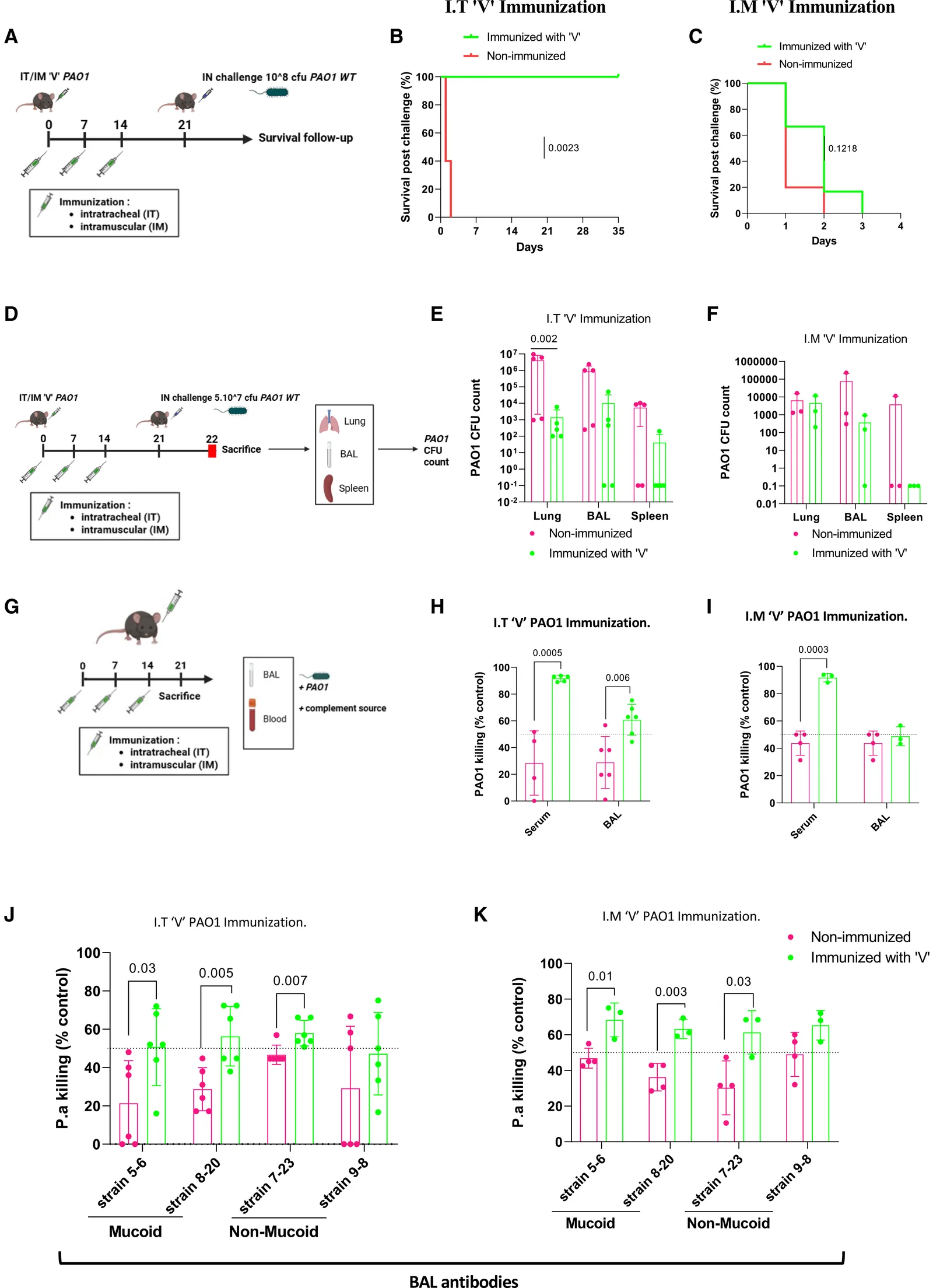
PAO1-WT challenge following either i.t. or i.m. immunization with the “V” strain C57BL/6 mice (A) were immunized i.t. (B) or i.m. (C) as in Figures 3 and 4. At day 21, 7 days after the last boost, mice (*n* = 6 in each group) were challenged i.n. with 10^8^ cfu of PAO1-WT. Mice were observed for survival (Mantel-Haenszel log rank tests). (D) C57BL/6 mice were immunized i.t. or i.m. and were challenged i.n. with a sub-lethal dose (5 × 10^7^ cfu of PAO1-WT). 24 h later, mice were sacrificed, and bacterial cfu counts were determined in lungs, BALs, and spleens (E and F). Two-way ANOVA comparisons were used to determine statistical significance (*p* < 0.05). Error bars represent standard deviation (SD). (G) Sera and BALs were collected from sacrificed animals (D) and were assessed for complement-dependent bactericidal assays: bacterial counts were determined after incubating PAO1-WT (H and I) or P.a clinical strains (J and K) with either sera or BALs with an exogenous source of complement (see materials and methods for details). Results are expressed as percent of numbers of PAO1-WT input load. Two-way ANOVA comparisons were used to determine statistical significance (*p* < 0.05). Error bars represent standard deviation (SD).

After demonstrating above the marked differences (and few similarities) between the i.t. and i.m. vaccination protocols in terms of immunogenicity, we then tested how this might affect protection against a lethal PAO1-WT (10^8^ cfu) challenge, performed i.n. 7 days after the last boost (Figure 6A). In addition, to emphasize the importance of mucosal immunization over that of intramuscular vaccination, we followed the same protocol using also an i.n immunization procedure (Figure S13A) and showed that both i.t. and i.n. immunization were clearly more efficient (Figures 6B and S13A) than i.m. at protecting mice (Figure 6C).

To assess the magnitude of bacterial infection, we infected different cohorts of mice using a sub-lethal challenge dose of 5.10^7^ cfu of PAO1-WT (Figure 6D) and quantified bacterial counts at 24 h post challenge. While the bacterial load was very significantly diminished in the lungs of i.t. immunized (Figure 6E), it remained very high in i.m.-vaccinated mice (Figure 6F). In order to determine whether antibodies may have played a role in the clearing of bacteria noted above, we then tested *ex vivo* the complement-dependent bactericidal activity of serum and BAL antibodies against PAO1-WT and against the same P.a mucoid (5–6, 8–20) and non-mucoid (7–23, 9–8) clinical strains mentioned above (Figure 3). In keeping with data showing that both i.t. and i.m. immunization protocols generated strong antibody levels (Figure 3), we showed that these antibodies (from serum and BALs for i.t.-immunized animals, Figure 6H) were able to activate the complement and kill all P.a strains (PAO1-WT) and clinical strains 5–6, 8–20, 7–23, and 9–8 (BAL antibodies, Figures 6J and 6K). However, notably, the level of antibodies present in the BAL of mice i.m. immunized was not sufficient to kill P.a (Figure 6I).

### Respiratory mucosal immunization with Δ*LasB-PAO1* “V” induces long-lasting humoral and cellular immune responses

**Figure 7:**
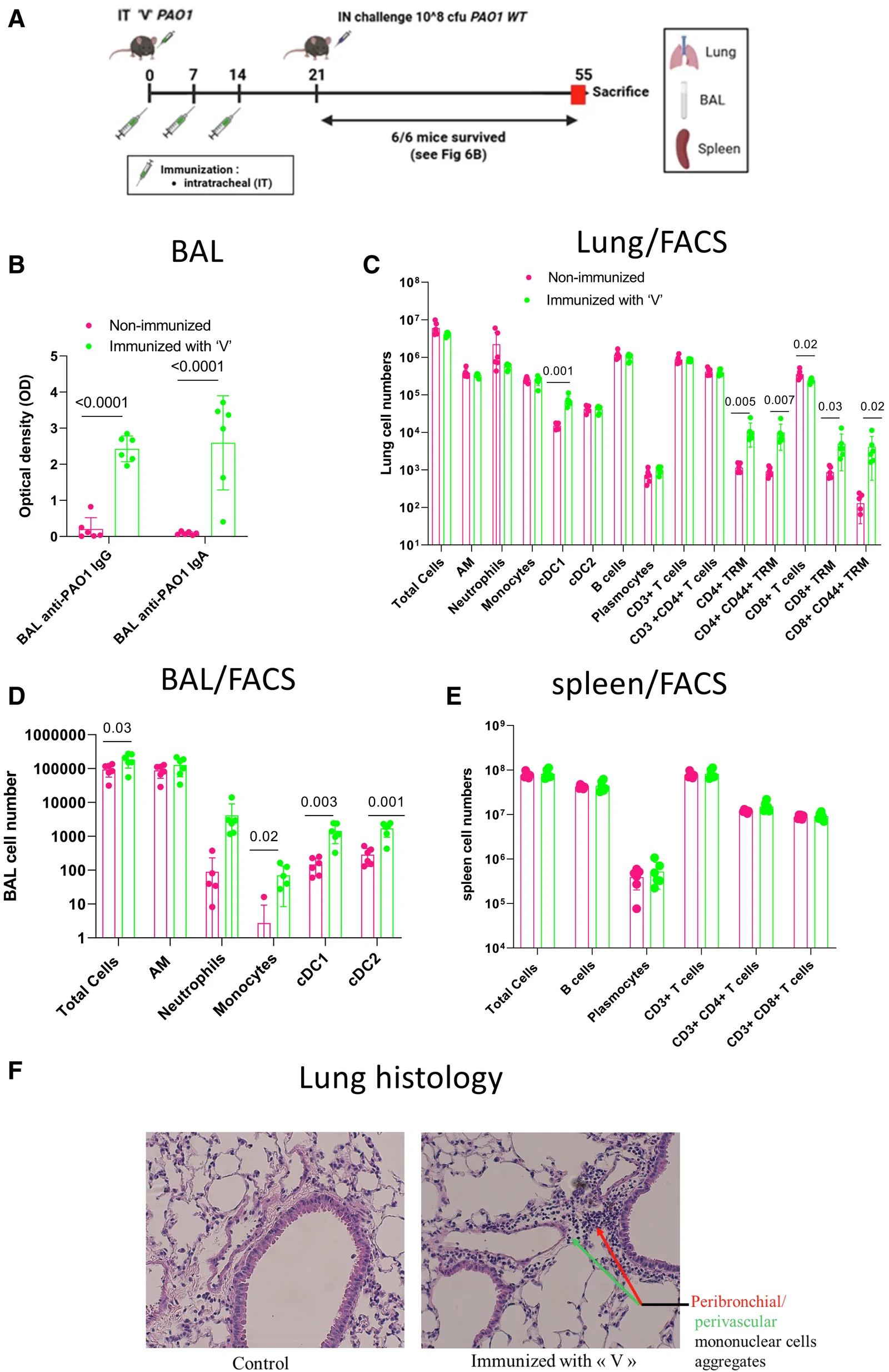
Evaluation of “intermediate” memory immune responses at day 55, i.e., 41 days post “V” i.t. immunization and 34 days post challenge with PAO1-WT (A) Mice that survived the challenge with PAO1-WT (6/6, Figure 6) were observed until day 55 (i.e., 34 days post challenge). They were then sacrificed, and lungs, BALs, and spleens were collected. (B) Anti-PAO1-WT IgG and IgA were measured in BALs. (C–E) BAL, lungs, and spleen cellular immune responses were analyzed by flow cytometry as in Figure 5. Multiple *t* tests were used to determine statistical significance (*p* < 0.05). Error bars represent standard deviation (SD). (F) Lung histology was performed as described in materials and methods.

To determine the “intermediate” and “long-term” longevity of “V”-induced memory immune responses, we first assessed lungs, BALs, and spleens of mice at days 55 and 69 (Figures 7A and S13B), respectively 34 days and 48 days after the original i.t. or i.n. “V” immunization protocol and the ensuing i.n. PAO1-WT challenge (which resulted in 100% survival; see Figures 6B and S13A).

Importantly, we found that the levels of specific anti-PAO1-WT IgG and IgA were maintained in these BALs and sera at these “intermediate” time points (Figures 7B and S13C–S13E) to levels at least equivalent as those observed at day 21 following “simple immunization” (Figures 3C and 3D). Also, the serum antibodies were able, in a complement-dependent fashion, to induce the killing of both PAO1-WT and “V” strains (Figure S13F).

When lung immune cells were investigated by flow cytometry, it is noticeable that although there was a slight reduction at days 55 and 69 (approximately 1 log and ½ log for days 55 [i.t., Figure 7C] and 2 log and ½ log for 69 days [i.n., Figure S13G]) in the levels of lung CD4 and CD8 TRMs, respectively, when compared with day 21 (Figure 4B), they were still very significantly increased in “V”-immunized mice compared with their non-immunized counterparts. In keeping with this, histological observation of lungs at day 55 showed peribronchial and perivascular mononuclear cell aggregates in immunized mice (Figure 7F).

In BALs, expectedly, inflammation had almost completely subsided at that time point since alveolar macrophages (AM) levels returned to normal values (between 95% and 98%; Figures 7D and S13H). However, although representing very minor populations in absolute numbers, monocytes, neutrophils, and DC levels were still increased in vaccinated and challenged animals at days 55 and 69 compared with untreated mice, particularly in i.t.-immunized mice (Figure 7D). In contrast, no differences in lymphocyte numbers were observed in the spleens between treated and control animals (Figures 7E and S13I).

**Figure 8:**
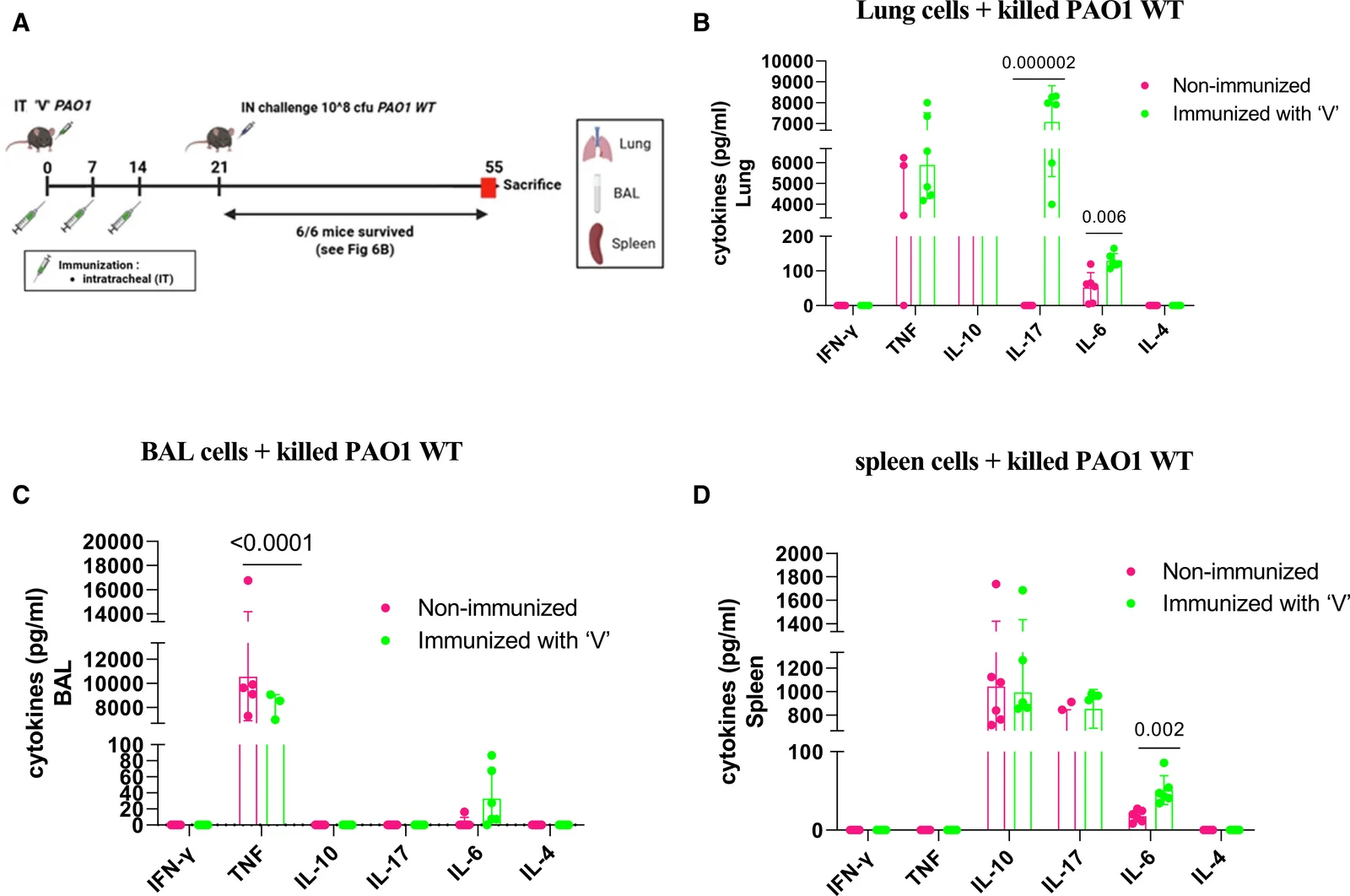
Cytokine output following long-term immunization with “V” and challenge with killed PAO1-WT (A) Same as Figure 7. Lung (B), BAL (C), and spleen (D) cells (see Figure 7) were incubated with gentamycin-killed PAO1-WT (MOI = 1), as in Figure 5 legend. Cell supernatants were collected and IFN-g, TNF, IL-10, IL-17, IL-6, and IL-4 cytokine levels were measured. Two-way ANOVA comparisons were used to determine statistical significance (*p* < 0.05). Error bars represent standard deviation (SD).

Upon *ex vivo* antigenic recall with killed PAO1-WT (using the same protocol applied post immunization on day 21; see Figure 5), lung parenchymal cells still secreted at these late time points (day 55 for i.t. immunization; Figure 8A; and day 69 for i.n. immunization; Figure S13B) impressively high levels of IL-17 (Figures 8B and S13J), respectively. Notably, lung cells also showed enhanced IL-6 secretion compared with non-immunized animals. Interestingly, BAL cells (and most likely AMs since they were the overwhelmingly predominant cell type in BALs at day 55 (Figure 7D) seemed to display a memory-like phenotype and responded differently to killed PAO1-WT in terms of TNF (statistical significance) and IL-6 production (trend) (Figure 8C). By contrast, surprisingly, after antigenic recall, splenic cell IL-17 levels from i.n. immunized animals were much higher than their i.t.-immunized counterparts (Figure S13K vs. Figure 8D), whereas splenic IL-6 levels were both increased in i.t. and i.n. protocols.

Finally, we analyzed the responses to a sub-lethal PAO1 WT challenge, 105 days (“long-term protocol”) after the last immunization boost (Figure S14A) and showed that compared with non-immunized mice, long-term vaccinated mice, exhibited, 15 days post challenge, still impressive increased anti-PAO1 WT serum IgGs and BAL IgAs (Figures S14B and S14C).

When flow cytometry was performed on lung cells, we showed that CD4 T cells were still increased, and there was a trend toward increased levels of CD4 TRMs in “V”-immunized + PAO1-WT treated mice (Figure S14D). Similarly, in BAL cells, there was a trend toward increased neutrophils in the latter group, compared with the “non-immunized” + PAO1-WT treatment (panel E). In contrast, no differences in lymphocyte numbers were observed in the spleens between the 2 groups (panel F).

As above, upon *ex vivo* antigenic recall with killed PAO1-WT, the cytokine mostly upregulated was IL-17, both in lungs and the spleen, and yet again, the induction was more marked in cells from V-immunized animals (Figures S14G and S14H).

### Respiratory mucosal immunization with Δ*LasB-*PAO1 “V” induces high levels of anti-bacteriophage humoral responses

Given the very significant presence of the secreted SI form of Pf4 bacteriophages and the prevalence of the expression of their genes in the “V” strain (see above Figures 1 and S1; Table S1), we also specifically evaluated here anti-phage humoral responses. After showing that all protocols of immunization used throughout (with PAO1-WT, *ΔLasB*-PAO1, *ΔLasB*-PAO1-HI, “V”) generated antibodies against PAO1-WT (Figure S10), we further demonstrated that all these protocols also induced, albeit at different levels, anti-phage antibodies in sera (IgG, Figure S15A) and in BALs (IgGs and IgAs) (Figures S15B and S15C). However, expectedly (since bacteriophages are strongly induced in the “V” strain), the levels of anti-phage antibodies were the highest when “V” was used as an immunogen, and as before, i.n./i.t. immunization was more effective than i.m. immunization.

In addition, to assess further the durability of the anti-phage antibodies, we analyzed the sera and BALs at day 120 as above (105 + 15). Indeed, we showed that “V”-immunized + PAO1-WT-treated mice had higher levels of serum anti-phage IgGs as well anti-phage IgGs and IgAs in BALs (Figures S15D and S15E).

## Discussion

Despite decades of work, most historical *P. aeruginosa* vaccines failed to progress beyond encouraging murine data, partly because they pre-dated the now well-recognized importance of respiratory mucosal route of immunization, IL-17-mediated mucosal immunity, and airway IgA.^4^^,^^5^^,^^6^^,^^7^^,^^9^ Earlier vaccine efforts focused mainly on inducing systemic IgG through parenteral delivery, a strategy that offers limited protection at the airway surface where colonization and biofilm initiation occur. Moreover, few studies examined IL-17 responses or evaluated mucosal antibody induction.^13^^,^^38^^,^^39^ Similarly, although some studies have explored auxotrophic P.a strain-based vaccines,^12^^,^^14^^,^^15^^,^^16^^,^^17^ to the best of our knowledge, none have developed, critically, CF-adapted or “CF-like” vaccine strains, which differ markedly from acute laboratory isolates in virulence traits, biofilm behavior, and immune evasion. Although more recent preclinical work has begun to incorporate mucosal adjuvants, intranasal platforms, and Th17-skewing approaches, this historical lack of attention to mucosal immunity and to clinically relevant chronic strains likely contributed to earlier failures and underscores the need for strategies that generate local, IgA- and IL-17-competent immunity against the phenotypes dominating chronic lung disease. Indeed, respiratory delivery should allow for more precise targeting of both the upper and lower respiratory tract, ensuring consistent dosing and potentially enhancing immunogenicity at the primary site of pathogen entry. This is well illustrated—albeit outside the field of P.a vaccination—by efforts in viral immunization, such as FluMist/Fluenz Tetra for influenza and ChAdOx1 nCoV-19 for SARS-CoV-2 in phase I trials.^40^^,^^41^^,^^42^^,^^43^^,^^44^^,^^45^ Relatedly, Jeyanathan et al. demonstrated in a recent phase I study that individuals who had received an mRNA COVID-19 vaccine (Pfizer and/or Moderna) showed no detectable lung mucosal immunity—neither humoral nor cellular—at least 3 months after their last dose. In contrast, inhaled aerosol delivery of a chimpanzee adenovirus platform (Ch-triCoV/Mac) expressing three SARS-CoV-2 antigens effectively filled this gap in respiratory immunity, generating measurable CD8 T cell responses (both circulating and resident memory TRM) and protective antibodies up to 48 weeks post vaccination.^45^

In that context, we developed and characterized here a novel P.a vaccine strain (“V”), by maintaining a *ΔLasB*-PAO1 strain in culture for 15 days in ASM (a culture medium mimicking CF sputum,^18^^,^^19^^,^^20^^,^^21^^,^^22^^,^^23^^,^^24^^,^^25^^,^^26^ secretions in which bacteria often show auxotrophy.^26^^,^^27^^,^^28^^,^^29^ As an attenuated *P. aeruginosa* strain, we selected this isolate based on our data showing that the LasB virulence factor is the most abundant protein in PAO1 secretomes,^46^^,^^47^ and that the *ΔLasB*-PAO1 strain—when cultured in standard LB medium—exhibited reduced virulence both *in vitro* and *in vivo*.^46^^,^^47^ Furthermore, numerous studies have demonstrated that LasB is present in clinical samples either in early- or late-stage CF disease^48^^,^^49^^,^^50^(for a recent review, see Everett and Davies^50^), and its presence has been associated with decreased antibiotic sensitivity and enhanced biofilm formation.^51^^,^^52^

A thorough WGS and RNA-seq analysis of our V strain (Figures 1, S1, and S2) demonstrated an increased expression of genes present in the core region of the filamentous prophage Pf4 and 3 genes of Pf6 (*pfkA*, *pfkB*, and *pfkC*), known to positively regulate Pf4. This induced the secretion of the SI form of the phage, known to be abundant in biofilms and to reduce, as demonstrated here, the twitching capacity of P.a.^31^^,^^32^^,^^53^ In addition to the phage genes mentioned above, KEGG Pathway Enrichment analysis revealed upregulation of several metabolic pathways in the “V” strain (including “Biosynthesis of secondary metabolites, phosphonate and phosphinate, amino acid, and various nucleotide sugars”), all pointing toward activation of stress programs. Under these metabolically stressful conditions, the strain also exhibited an apparent auxotrophy for tryptophan, tyrosine, and phenylalanine. This functional auxotrophy, combined with the metabolic adaptations observed, reinforced the idea that the strain’s physiological profile closely mirrored the nutrient-restricted and redox-challenged environment of the mucoid CF sputum.^18^^,^^19^^,^^20^^,^^21^^,^^22^^,^^23^^,^^24^^,^^25^^,^^26^^,^^35^

As a whole, this validated our hypothesis that the “V” strain may indeed be a highly relevant and promising vaccine candidate as a “CF-mimic” strain, including in patients with cystic fibrosis. Indeed, despite very significant advances in the field through the use of modulators and correctors,^2^^,^^3^ significant side effects exist, and a sizable numbers of patients do not have access to these medicines, justifying the need for further approaches, including vaccinal ones.

Our data indicate that the engineered strain exhibited reduced *in vitro* and *in vivo* virulence (Figures 2 and S4) and was globally at least as immunogenic as other PAO1 strains (PAO1-WT, *ΔLasB*-PAO1, *ΔLasB*-PAO1-HI) cultured in classical LB medium, in terms of induction of antigen-specific cellular immune responses both in the airways and lung tissue (Figures S11 and S12), of antibody responses (notably IgAs) (Figures 3B–3E and S10), and in terms of lung IL-17 immunity (Figures 4B–4D, 5A–5C, S13J, and S13K).

Importantly, only respiratory immunization protocols significantly protected mice against a lethal challenge of PAO1-WT (Figures 6 and S13), and immune memory responses lasted for at least 120 days (Figure S14) in keeping with the increasingly recognized importance of mucosal immunization. Indeed, the antigen presentation and the associated immune responses including chemotactic and cell adhesion signals are expected to differ in totally different tissue environments following different routes of immunization. Ample preclinical^42^^,^^54^^,^^55^^,^^56^ and clinical data^42^ have shown that differently from i.t route, , the i.m. route of immunization results in immune responses sequestered within the peripheral blood and lymphoid tissues unless local lung immune signals are introduced.

Interestingly, we showed that the “V” strain, which greatly upregulated phage genes as discussed above (Figure 1) also induced increased anti-phage IgG and IgA antibodies up to 120 days (Figure S15), compared with the other PAO1 strains used.

This may be particularly relevant given the reported anti-phagocytic activity of these phages,^30^ and consistent with this, Sweere et al. showed that anti-phage antibodies can significantly reduce wound infections caused by *P. aeruginosa*.^30^

Notably, the vaccine also conferred protective efficacy in mice lacking CD4 T cells during the effector phase of responses (Figure S9), suggesting that immunogenicity can be maintained even in a compromised immune setting. Mechanistically, although vaccine-induced protection was associated with increased IL-17 and IgA responses in CD4+ immunocompetent mice, this suggests the presence of redundant or compensatory protective mechanisms in CD4-mice, and that IL-17 may serve as correlate rather than direct mediator of immunity in this model. Also, even if directly implicated, IL-17 production may derive from CD4^−^ cellular sources, including gamma delta T cells, natural killer T cells, and innate lymphoid cells, which could contribute to protection independently of conventional CD4^+^ T cells.^57^^,^^58^^,^^59^^,^^60^^,^^61^^,^^62^

Beyond cystic-fibrosis-associated disease, significant unmet need also exists in non-CF respiratory infections, in COPD, bronchiectasis, or in hospital-acquired settings such as ventilator-associated pneumonia, where acute infection by opportunistic and often polymicrobial pathogens remains a major contributor to intensive care unit morbidity and mortality.^4^^,^^5^^,^^6^^,^^7^^,^^63^^,^^64^

In contrast to the chronic colonization characteristic of CF airways, these infections arise in the context of epithelial barrier disruption and critical illness, suggesting a complementary but distinct clinical niche for preventive and/or early therapeutic vaccination strategies. More broadly, similar considerations may apply to other high-risk populations with impaired barrier integrity, such as burn patients, in whom invasive bacterial infections continue to represent a major cause of adverse outcomes.

It should be noted that this study has several limitations, including the lack of in-depth mechanisms regarding the differential role of immune cells in the protective phenotype. In addition, even though it has been previously shown by others that experimental vaccines that were protective against PAO1 also offered enhanced protection against P.a strains of greater clinical relevance,^65^^,^^66^^,^^67^ further studies will be needed to confirm this with the V strain. Finally, to date, vaccine strategies against *P. aeruginosa* that have progressed into clinical evaluation have largely relied on killed whole-cell, polysaccharide, or protein-based formulations, including formaldehyde-inactivated vaccines, which demonstrated immunogenicity but limited clinical efficacy. In contrast, LAV approaches (e.g., auxotrophic or genetically attenuated strains) have shown promising protection in preclinical models but have not yet advanced into clinical trials.^4^^,^^5^^,^^6^^,^^7^^,^^9^^,^^11^ Further preclinical and clinical evaluation of V will therefore be equally essential to balance the potential immunogenic benefits with the inherent risks of a live vaccine platform.

## Materials and methods

### Ethical statement

Animal experiments were carried out at the animal facility of the Paris-Cité Faculty of Medicine, Bichat site in Paris, in accordance with current regulations (Directive 2010/63/EU of September 22, 2010, and Decree no. 2013–118 of February 1, 2013, on the protection of animals used for scientific purposes). The protocol was approved by an animal experimentation ethics committee approved by the French Ministry of Higher Education and Research (APAFIS number: 27834).

Please see the supplemental information for the following: bacterial strains, culture, RNA-seq and whole genome sequencing; bacterial auxotrophy and motility tests; and cell line culture, maintenance, and bacterial infection.

### *In vivo* experiments

#### Animals, anesthetizing procedures, and bacterial infection

7- to 10-week-old male C57BL/6 mice were purchased from Janvier Labs. Animals were kept in a specific pathogen-free facility under 12-h light/dark cycles, with free access to food and water. Procedures were approved by our ethical committee and by the French Ministry of Education and Research (APAFIS number 27834). Prior to *in vivo* procedures, mice were anesthetized by intraperitoneal injection of 150 μL of a solution containing ketamine (10 mg/mL) and 2% Rompun (xylazine, 2 mg/mL) diluted in 0.9% NaCl. Bacteria were prepared from a frozen stock. After overnight culture, they were then diluted into fresh LB medium and incubated until reaching the exponential growth phase (OD_600_ between 0.4 and 0.8). Bacterial concentration was estimated based on the relationship that an OD_600_ of 1 corresponds to 10^9^ bacteria/mL. Immediately after administration, the exact number of administered bacteria were checked post hoc by diluting the vaccine dose (20 μL) on LB agar. Plates were incubated at 37°C overnight and colonies counted in order to determine that number.

#### *In vivo* assessment of PAO1-WT and “V” differential clearance and virulence

C57/Bl6 mice were infected i.n. with 5 × 10^7^ cfu of either PAO1-WT or V. 24 h later, animals were sacrificed, and lungs, BALs, and spleen were recovered for CFU assessment. Cytokine levels were also measured by ELISA in BALs or spleen.

In independent experiments, to compare the virulence of PAO1-WT and V, mice were similarly infected with various doses of bacteria, and body weights and survival were followed over time.

#### *In vivo* immune responses analysis

C57/Bl6 mice were either mock-immunized (PBS) or immunized with three doses of various PAO1-WT strains (10^6^ cfu of vaccine “V” PAO1 strain, PAO1-WT, *ΔLasB*-PAO1, *ΔLasB*-PAO1-HI, a dose chosen based on our historical experience on “benign,” sub-lethal, and lethal doses) or with PBS, i.m. or mucosally (i.n./i.t.). Immunizations were performed via either the oropharyngeal/i.t. (the two terms are used indiscriminately throughout the manuscript) or i.n. at weekly intervals. This short interval was chosen to promote a rapid induction of the adaptive immune response, in line with previously described murine models using short intervals for the evaluation of highly immunogenic vaccines. This was followed by collection of blood, lungs, spleen, and BAL either at day 21, i.e., 7 days after the last boost, or at days 55 or 69, i.e., 34 or 48 days post PAO1-WT challenge (“intermediate memory experiments”). Alternatively, the responses to a sub-lethal PAO1 WT challenge, 105 days after the last immunization boost (3 immunizations, 1 week apart, as above) were analyzed 15 days after (“long-term memory experiments”). Organs and body fluids were then analyzed (see below and the supplemental information) with a variety of *ex vivo* readouts (flow cytometry, ELISAs, bacterial counts and killing assays, *ex vivo* antigenic recall, histology).

#### Mouse survival after immunization and challenge

After immunization with the “V” strain, either i.t., i.n. or i.m, (or mock-immunized with PBS) as explained above, different cohorts of mice were challenged at day 21 i.n. with a lethal dose of PAO1-WT (10^8^ cfu) and were monitored for survival.

#### Role of CD4 T cells in the protective phenotype

Mice were mock vaccinated with PBS or with “V” as in Figures 6 and S13. At day 20, they were injected intraperitoneally with 250 μg of anti-CD4 antibody (or isotype) and i.n. with 30 μg of the same antibodies. At day 21, this was repeated i.n. with 30 ugs of antibodies. At day 23, 2 mice from each group were anesthetized and injected retro-orbitally with 2 ugs of anti-CD45-PB for intra-vascular labeling. 3 min later, these mice were sacrificed, and spleen and blood were retrieved, as well as lungs (after perfusion), for assessment of CD4 T cell depletion and CD45 labeling. The reminder mice in each group were challenged with a lethal dose of PAO1-WT (10^8^ cfu), and survival was assessed.

#### Measurement of anti-P.a-specific antibodies: Whole-cell ELISA

Blood and bronchoalveolar lavage fluid samples were taken at day 21 after mock immunization or after days 21, 55, 69, and 120 (see materials and methods). PAO1-WT bacteria were inactivated at 90°C for 60 min after overnight culture, and centrifuged at 20,000 × *g* for 10 min. Resuspended pellets were diluted to a DO_600_ of 0.2, and 100 μL of the different bacterial suspensions was transferred to 96-well ELISA plates, which were incubated overnight at 37°C. After blocking with 1% bovine serum albumin and washing with PBS containing Tween 20, 100 μL of mouse samples (serum and BAL, diluted 1/2) was added to each well and incubated for 2 h at 37°C. Next, after further washing, 100 μL of a 1:10,000 dilution of secondary anti-mouse antibodies (IgG [for serum and BAL], anti-H&L horse [for BAL], IgA anti-H&L goat, Abcam, Paris, France) coupled to horseradish peroxidase was added. After further washing as above, 100 μL of TMB substrate (Sigma T0440-1L, France) was then added for 30 min. In the absence of a standardized reference curve using purified antigen-specific antibodies, OD readings were performed at 450 nm using a TECAN reader.

### Data analysis

For most of our experiments, our group sizes (*N* = 5–8) were calculated to provide >80% power and a significance level (α) of 0.05 to detect a 1-log difference in most of our readouts. Mice were randomized upon arrival from the vendor to ensure equal distribution of weights and age across cohorts. While investigators were not blinded during the vaccination phase, downstream experiments were performed by researchers blinded to the treatment groups to eliminate observer bias. Statistical analyses were performed using GraphPad Prism v10. Results are expressed as mean ± SD. Significance was determined using two-tailed Mann-Whitney tests, one-way ANOVA with multiple comparisons, or Mantel-Haenszel log rank tests, with *p* < 0.05 considered significant.

## Data and code availability

• All reagents will be available upon reasonable request, and all data are available in the main text or the supplemental information.
• The complete genomic and annotated sequences were deposited to NCBI GenBank: JBRXYX000000000 and JBRXZF000000000 (for PAO1-WT and “V” strains, respectively).

## Acknowledgments

We wish to thank Mses Clémentine Cornu, Christelle Muñoz, and Blandine Arleri (JMS’ laboratory) for their help with some of the experiments and Dr. Brice Barbat (LCB-UMR7283) for generating the *ΔLasB*-PAO1 mutant. We also thank Drs. Loredana Saveanu (CRI/INSERM U1149, Paris) and Imane El Meouche (INSERM, IAME, Paris) for useful discussions. We are also indebted to Prs. Catherine Llanes and Patrick Plésiat (Université Marie et Louis Pasteur, CNRS, Chrono-environnement [UMR 6249], Besançon, France) for their gift of the P.a clinical strains used here, to *Vaincre la Mucoviscidose* (grant RF20230503231), to a COPOC funding from 10.13039/501100001677Inserm (MAT-API-00990-A-02), and to the association *Gregory Lemarchal*. Thanks also go to Pr. E. Tartour (Hôpital Européen Georges-Pompidou, Paris) for reviewing a draft of this manuscript.

## Author contributions

Conceptualization: Y.S., J.-M.S.; investigation: Y.S., B.V., S.G., S.I., H.L., D.B., F.M., M.B.B., J.-M.S.; formal analysis: Y.S., B.V., S.C., F.M., J.-M.S.; resources: J.-M.S., R.V.; writing – original draft: Y.S., J.-M.S., S.C., F.M.; writing - review and editing: Y.S., B.V., S.C., S.I., F.M., C.D., M.B.B., Z.X., R.V., J.-M.S.; project administration: J.-M.S.; supervision: J.-M.S.; and funding acquisition: J.-M.S.

## Declaration of interests

The authors declare that they have filed a patent application related to the technology described in this manuscript.

## Supplemental information

Document S1. Figures S1–S15 and Tables S2 and S3

Table S1. Genes upregulated in “V” versus PAO1-WT (log2fold change >1 and *p* < 0.05

Document S2. Article plus supplemental information
