# Supplementary material for: A novel cystic fibrosis-mimetic Pseudomonas auxotrophic vaccine is protective in vivo and is associated with IL-17/IgA mucosal Immunity
Source: Mol Ther. 2026 Jun 17;34(9):5302–18. doi: 10.1016/j.ymthe.2026.06.029 (PMC13555540; doi:10.1016/j.ymthe.2026.06.029)
Supplement: Supplementary file 1 [file mmc1.pdf]

YMTHE, Volume *34*

Supplemental Information

A novel cystic fibrosis-mimetic *Pseudomonas* auxotrophic vaccine is protective *in vivo* and is associated with IL-17/IgA mucosal Immunity

Youssouf Sereme, Bérengère Villeret, Ségolène Caboche, Samuel Ikeh, Hugo Leroy, Delphine Beury, Florence Maurier, Christophe Desterke, Maëlys Born-Bony, Zhou Xing, Romé Voulhoux, and Jean-Michel Sallenave

Supplemental information

Supplemental Materials and Methods

Bacterial strains and culture

PAO1-WT and clinical strains are described in Born-Bony M et al. (1) (**Table S3**). Briefly, PAO1 WT belongs to the serogroup 5 whereas the clinical strains 8-20 and 7-23 belong to the serogroups 2 and 16, respectively (the clinical strain 9-8 was non-typable).

Bacterial strains were initially recovered from frozen stocks (–80 °C) and streaked onto LB agar plates, followed by overnight incubation at 37 °C. A single isolated colony was then inoculated into liquid LB medium to prepare a preculture, which was incubated overnight at 37 °C under agitation (180–200 rpm). This preculture was used to inoculate a main bacterial culture, grown under the same conditions until reaching the exponential growth phase (OD600 ≈ 0.6–0.8).

ΔLasB- PAO1 was constructed from PAO1-WT strain(2) according to the protocol of Kaniga et al.(3) Briefly, to construct the pKNG101*ΔLasB* plasmid, two DNA fragments corresponding to the ~500-bp upstream and ~500-bp downstream regions of *LasB* gene (PA3724) were respectively amplified from PAO1 chromosomal DNA by PCR using primer couples A (5'CAGGTCGACGGATCCCCGGGTTGCCTTCAACATCGACCGC3') - B (5'ACCGAGCTTACTTCATCTTGTTCAGTTCTCC3') and C (5'AACAAGATGAAGTAAGCTCGGTGGTCCC3') – D 5'TATGCATCCGCGGGCCCGGGGGATTCGATGAAACGGGTGATG3') and ligated by overlapping PCR using primer couple A-D and AB & CD PCR products as DNA template.

The overlapping PCR product was cloned into linearized pKNG101 by the SLIC method. The resulting constructs were transformed into *E. coli* CC118λpir and introduced into P.a PAO1-WT by conjugation. The strains in which the chromosomal integration event occurred were selected on P.a isolation agar gentamycin (Gm) plates. Excision of the plasmid, resulting in the deletion of the chromosomal target gene was performed after selection on Luria-Bertani (LB) plates containing 6% sucrose. Clones that became sucrose resistant and Gm sensitive were confirmed to be deleted for the *LasB* gene by PCR analysis using primer couple E (5'GAGCCGATGTTCCACGGGG3' - F 5'ATCCAGCCGCCGTCGCCGA3'). The vaccine strain *ΔLasB*-PAO1 ('V' strain) was then obtained by sequential growth over 15 days in artificial sputum medium (ASM, complete composition given in[18]), by changing the ASM medium every four days. Before experiments, this 'V' strain and other P.a strains (PAO1-WT, *ΔlasB*-PAO1, *ΔlasB*-PAO1-HI (heat-inactivated), mucoïd and non mucoïd clinical strains, all cultured in LB medium) were grown overnight at 37 °C on LB agar plates. Single colonies were then inoculated into 10 mL of LB broth and incubated overnight at 37 °C with shaking (180 rpm) to prepare a pre-culture. The optical density (OD) at 600 nm was measured, and the bacteria were then diluted in fresh LB to reach an OD of 0.08. This culture was incubated at 37 °C with shaking for 2–3 hours until it reached exponential growth, corresponding to an OD between 0.4 and 0.8. The OD was measured again before further use.

To assess the impact of both genotype and culture conditions (24hrs or 15 days, under continuous selective pressure), six groups were defined at the onset of the study: PAO1-WT-LB-24hrs, PAO1-WT-ASM-24hrs, PAO1-WT-ASM 15days, *ΔLasB*-PAO1-LB-24hrs, *ΔLasB*-PAO1-ASM-24hrs, and *ΔLasB*-PAO1ASM-15days ('V' strain).

The phenotype of the V strain has been analysed through various experiments (see below) over 70 sequential passages.

RNA-Seq and Whole genome sequencing analysis

P.a PAO1-WT and *ΔLasB*-PAO1 strains were grown as indicated above, either in LB or in ASM, during 24hrs or 15 days. All conditions were performed in biological replicates, resulting in 23 RNA samples in total. Total RNA was extracted using the NucleoSpin RNA Kit (Macherey-Nagel, ref. 740955.50) according to the manufacturer's instructions. Ribosomal RNA was depleted using the QIAseq FastSelect -5S/16S/23S kit (Qiagen), and stranded libraries were prepared using the QIAseq Stranded RNA Library Kit (Qiagen). Sequencing was performed on an Illumina NovaSeq 6000 platform in paired-end mode (2 × 150 bp). Raw RNA-seq data have been deposited in the NCBI Sequence Read Archive (SRA) under BioProject accession number PRJNA1293891.

Reads were pseudo-aligned to a custom P.a transcriptome, based on GenBank: JBRXYX000000000 sequence. Transcript abundance was quantified using the nf-core/rnaseq v3.18.0 pipeline(4) and kallisto v0.51.1(5). Processed count data are available in the Gene Expression Omnibus (GEO) under accession number GSE303184. Differential expression analysis was performed using DESeq2 v1.38.3(6) and R v4.2.2 . Gene Set Enrichment Analysis (GSEA) was performed using clusterProfiler v4.6.2(7) and the Kyoto Encyclopedia of Genes and Genomes (KEGG) database to identify significantly enriched biological pathways. For q-PCR analysis, q-PCR of the Pf4 r gene (Pf4r-qF : AGTTTGGCCCGACTTCTGGG ; Pf4r-qR : CGTCAGCCCGATAGAGCAAGA) amplified a 206pb fragment in the the 3.3 kb DR (discarded region), absent in the excised SI Pf4 phage. Also, a q-PCR analysis of the circularization site of Pf4 (attP), located between the integrase gene and the repressor genes was performed (Pf4 FWD : TAGGCATTTTCAGGGGGCTTGG ; Pf4 REV : GAGCTACGGAGTAAGACGCC), amplifiying a 135 bp fragment. This allowed for the discrimination between the circularized Pf4 DNA and the linear Pf4 prophage sequence.

Whole genome sequencing (WGS)

The bacterial strains were cultured in LB medium overnight at 37°C. PAO1-WT-LB-24hrs and *ΔLasB*-PAO1-ASM-15d 'V' strain genomic DNA was extracted with the QIAamp DNA mini kit (Qiagen). gDNA quality was evaluated using TapeStation 4200 (Agilent Technologies), and quantified by spectrophotometry (Nanodrop One, Thermo Fisher), and fluorimetry (Qubit, ThermoFisher). The libraries were prepared with the Native barcoding kit v14 (SQK-NBD 114.24, Oxford Nanopore Technologies), and following the Ligation Sequencing gDNA Native Barcoding for PromethION protocol (Oxford Nanopore Technologies). The sequencing was done with a PromethION flowcell on a PromethION 2 solo sequencer (Oxford Nanopore Technologies), with the super accuracy live basecalling mode, during 72 hours.

Adapters were trimmed from the raw reads using Porechop v0.2.4(8), and reads shorter than 500 bp were filtered out using NanoFilt v2.7.1(9). Sequences corresponding to the ONT internal positive control (DCS—DNA Control Strand) were removed by mapping reads to the DCS sequence with Minimap2 v2.28-r1209(10), using the -ax map-ont option, and retaining only unmapped reads. A total of 387,000 reads were retained for the PAO1-WT strain and 353,000 reads for the 'V' strain. Reads have been deposited in SRA under BioProject accession number PRJNA1353604. De novo assembly was performed using Flye v2.9.3-b1797(11) with the options --nano-hq --scaffold --asm-coverage 50 --genome-size 6.2m. Assembled sequences were compared using DNAdiff v1.3 from the MUMmer package(12). Manual curation was carried out by visualizing read mappings in the Integrative Genomics Viewer (IGV)(13).

Genome annotation was performed using NCBI prokaryotic genome annotation pipeline (PGAP)(14). Complete genomic and annotated sequences were deposited to NCBI GenBank under JBRXYX000000000 and JBRXZF000000000 IDs, respectively, for the PAO1-WT and 'V' strains.

Bacterial auxotrophy test, pyocyanin and pyoverdine measurement.

The various PAO1-WT strains were cultured until they reached the exponential growth phase (DO between 0.4 and 0.8), and the optical density (OD) was measured at 600 nm. The bacteria were then resuspended in 10 mL of laboratory-made M9 minimal medium (Gibco by Life Technologies) to obtain an OD of 0.05. Incubation was carried out at 37°C with agitation (210 rpm). DO was measured every two hours for bacteria grown in M9 medium and the *ΔlasB*-PAO1-ASM-15j-vaccine 'V' strain was also, when needed, grown in M9 medium supplemented with aromatic amino acids (40 µg/mL tryptophan, tyrosine and phenylalanine)(15). Pyoverdine and pyocyanin secretion was measured in PAO1 WT and V secretomes, at 405 nm and 695 nm, respectively, as in (16).

Bacterial motility assays

PAO1-WT strains were grown to exponential phase (OD_{600} = 0.4–0.8). Swimming and swarming were assessed by spotting 5 µL of culture at OD_{600} = 0.1 on 0.3 % agar plates. Plates were incubated at 37 °C for 8 h (swimming) or 24 h (swarming), and motility zones were measured. Contraction motility was evaluated by spotting 5 µL at OD_{600} = 0.1 on 1 % agar overlying plastic. After 24 h, the agar was removed and bacterial spread on the plastic was measured.

Cell line culture and maintenance

DC2.4 dendritic cells (17) were cultured in IMDM supplemented with L-glutamine, HEPES, 10 % FBS, and penicillin/streptomycin. Cells were seeded at 2-3 × 10^4 cells/mL in 100 mm dishes and passed when they are confluent after observation under a microscope. Adherent cells were detached using PBS with 10 mM EDTA. MUTU (DC1*,* MPI alveolar macrophages(18) and DJS-2 epithelial cells(19) were cultured similarly, using IMDM (MUTU), RPMI (MPI with 30 ng/mL mGM-CSF), or DMEM media (DJS-2). Adherent cells were recovered by EDTA or trypsin treatment, counted, and replated to 1 to 2.10^6 cells in a T150 flask in 30 ml of medium. Healthy and CF primary bronchial epithelial cells (Mucilair^{TM}) were obtained from Epithelix (Geneva, Switzerland), and cultured according to the manufacturer's instructions.

Cell line culture and *in vitro* bacterial infection

Cells (5 × 10^4) from the indicated cell lines (see above) were seeded into 96-well plates in the appropriate culture medium (see above) and incubated overnight at 37 °C. Following bacterial culture, the different strains were resuspended in the corresponding cell culture media supplemented with 10% fetal bovine serum (FBS) without antibiotics, and infected with bacteria at a multiplicity of infection (MOI) of 1 (corresponding to 5 × 10^4 bacteria per well).

After appropriate times (3-4 hours for MPIs, 4 hours for Epithelix cells and 24 hours for the other cell lines (DC2.4, MUTU, and DJS-2), and after centrifugation, cell culture supernatants were then removed and stored for subsequent cytokine quantification assays (ELISA RnD Diagnostics).

In vitro PAO1 adhesion assay

Cells (3×10^5 per well) were seeded in 16-well plates and incubated overnight at 37 °C in appropriate medium with 10 % FBS and antibiotics. PAO1 bacterial strains were prepared in media (see section above) with 10 % FBS and used to infect cells at a multiplicity of infection (MOI) = 1 during 1h. After washing thrice with PBS, cells were lysed with 500 µL PBS containing 0.02 % Triton on ice for 10 min. Released bacteria were plated on LB agar and incubated overnight at 37 °C for CFU enumeration.

In vitro viability assay

Cells were plated in 96-well plates at 5×10^4 per well and incubated overnight. Cells (MPI, DC2.4, Mutus) were infected (MOI = 1) and incubated for 3 h (MPI) or 24 h (other cell lines, see above). Ten microliters of MTT labeling reagent (4,5-dimethylthiazol-2-yl)-2,5-diphenyltetrazolium bromide; Cell Proliferation Kit I (MTT), Sigma-Aldrich) was added to each well, followed by incubation for 4 hours at 37 °C. After incubation, the supernatant was removed and 50 µL of solubilization buffer (Cell Proliferation Kit I (MTT), Sigma-Aldrich) was added to each well. Plates were then incubated for an additional 30 minutes at 37 °C to allow complete solubilization of formazan crystals. Absorbance was measured at 540 nm using a TECAN microplate reader

Ex-vivo tissue analysis

a)Flow cytometry analysis

BAL cells were pelleted, resuspended in DMEM with 10 % FBS and counted. After perfusion, lung tissue was digested with collagenase (1mg/mL) (Sigma C2139-500MG, France)/DNase (50 µg/ml) (Signa DN25-10 MG, France), filtered with 70 µm filters and then 40 µm filters (Gigma, France), centrifuged, treated with ACK lysis buffer, washed, and suspended (2.10^6 cells) in a final volume 100 µl PBS1X. Spleen cells were homogenized in a single-cell suspension through mechanical disruption. Briefly, the spleens were grinded, filtered on a 40µm sterile filter and 2.10^6 cells were resuspended in 100 µl PBS1X. All cells were first incubated (10 min, 4°C) with a mixture of a viability dye and Fc Block antibody, washed (2,000 rpm, 5min) with FACS buffer (PBS, 1% BSA, 0.5Mm EDTA) and then incubated (30 min, 4°C) with a cocktail of cell surface conjugated antibodies in FACS buffer with either a myeloid or lymphoid antibody panels (see **Table S2** for a list of antibodies). For intra-cellular IL-17 labelling, 10^6 lung cells were stimulated with PMA/Ionomycin in the presence of monensin and brefeldin A for 4 h. Data were acquired the same day with a LSR Fortessa cytometer (BD Biosciences) with BD FACSDiva software and analysed with FlowJo (Tree Star, Ashland, OR, USA).

b) Lung histology

Perfused and fixed lungs (4%PFA in PBS, overnight at 4°C) were embedded in paraffin, sectioned in 4um slides and then stained with Hematoxylin-eosin by the INSERM U1149 'Plateforme de Morphologie'. Observations and microscopic photographs were taken at a 20X magnification.

c) Ex-vivo antigenic recall with killed PAO1-WT *and cytokine measurements*

Lung (10^6), spleen (10^6), and BAL (2×10^4) cells were plated (96-well plate) and stimulated with gentamicin-killed PAO1-WT for 48 h in RPMI with 10 % FBS in a total volume of 0.2 ml. After centrifugation at 500g, supernatants were collected and analyzed for TNF- α, IFN-γ, IFN-α, IL-6, IL-4, and IL-17 by sandwich ELISA (Duoset Bio-Techne, France).

d) Complement-dependent bactericidal activity assay

Complement-dependent bactericidal activity was evaluated in serum and bronchoalveolar lavage (BAL) fluid from immunized and non-immunized mice against the PAO1 strain and four clinical isolates (5-6, 8-20 [mucoid], 7-23, and 9-8 [non-mucoid]), using a previously described method (20). Briefly, bacteria were grown overnight at 37°C in liquid LB medium and then diluted to an optical density at 600 nm (OD600) of 0.05. Cultures were incubated at 37°C until reaching the exponential growth phase (OD600 between 0.4 and 0.6). Bacteria were then washed twice and resuspended in PBS. An appropriate volume of the bacterial suspension was adjusted to obtain a final concentration of 10^4 colony-forming units (CFU).

For the assay, 10 µL of bacterial suspension containing 10^4 bacteria were mixed with 10 µL of heat-inactivated serum (65°C for 45 minutes), 10 µL of rabbit complement (3–4 weeks old; Pel-Freez® Biologicals, AR, USA, 15,20), and 10 µL of PBS in a 96-well plate, and the mixture was incubated at 37°C for 1 hour. After incubation, serial dilutions in PBS were performed, followed by plating on solid LB medium and overnight incubation at 37°C. Bacterial clearance was calculated by comparing the number of viable PAO1 and clinical strains at the beginning and end of the assay. Heat-inactivated sera from non-immunized mice were used as negative controls. Bactericidal activity greater than 50% in at least two dilutions was considered as a positive result.

Purification and quantification of bacteriophages

Bacteriophages were purified according to the protocol described by Sweere et al.[21] Briefly, the 'V' strain was cultured in 75 ml of LB broth for 48 hours at 37 °C with agitation. Bacteria were removed by centrifugation at 6,000 × g for 5 minutes, and the supernatant was treated with 1 µg/mL DNase I (Roche, ref. 4716728001) for 2 hours at 37°C before being sterilised by vacuum filtration through a 0.22 µm filter. Pf phages were precipitated from the supernatant by adding 0.5 M NaCl and 4% polyethylene glycol (PEG) 8000 (Sigma ref. P5413). All phage solutions were incubated overnight at 4°C. Then, they were centrifuged at 13,000 × g for 20 minutes, and the pellet was resuspended in sterile TE buffer (pH 8.0). The suspensions were again centrifuged at 15,000 × g for 20 minutes, and the supernatants were subjected to another cycle of PEG precipitation. The purified phage pellets were then resuspended in sterile PBS and concentrated on an Amicon UltraCell 100 (100 kDa molecular weight cut-off), and their DNA concentration checked by PCR

(not shown). The final solution was sterilized by filtration with a 0.2 µm filter (Millex™ polyethersulfone syringe filter, reference: 83.1826.001, Germany).

Measurement of anti-phage specific antibodies (whole cell ELISA).

Blood and bronchoalveolar lavage fluid samples were collected as indicated above (in the 'In *vivo experiments'* section e of the Main M&M). Purified phages were diluted in PBS and 100 µl of different dilutions were transferred to 96-well ELISA plates, which were incubated overnight at 37°C. After blocking with 1% bovine serum albumin (BSA) and washing with PBS, 100 µL of mouse samples (serum and BAL, diluted 1/2) were added to each well and incubated for 2 h at 37°C. Next, 100 µL of a 1:10000 dilution of secondary anti-mouse antibodies were added and readings performed as above (M&M, section E).

Supplemental Table legends

Table S1 : Genes up-regulated in 'V' versus PAO1-WT (log2fold change >1 and p<0.05), see Excel file.

Table S2 : List of antibodies used for flow cytometry experiments

Antibodies	Fluorochrome	Isotype	Clone	Provider	Cat number	Dilution
CD45	PE/Cyanine7	Rat IgG2b, k	30-F11	Biolegend	103113	1/200
CD11b	PerCP/Cyanine5.5	Rat IgG2b, κ	M1/70	Biolegend	101227	1/200
CD11c	Pacific Blue	Armenian Hamster IgG	N418	Biolegend	117321	1/200
Siglec F	BV711	Rat LOU, also known as Louvain, LOU/C, LOU/M IgG2a, κ	E50-2440	BD Biosciences	740764	1/200
I-A/I-E	APC	Rat IgG2b, κ	M5/114.15.2	Biolegend	107613	1/200
Ly6G	Alexa Fluor 700	Rat IgG2a, κ	1A8	Biolegend	127621	1/200
Ly6C	PE	Rat IgM, κ	AL-21	BD Biosciences	560592	1/400
Fixable Viability Dye	eFluor 506			eBioscience	65-0866-14	1/1000
TruStain FcX ™ (anti-mouse CD16/32)		Rat IgG2a, λ	93	Biolegend	101320	1/200
Mixed extra-myeloid cell marking						
CD45	PE/Cyanine7	Rat IgG2b, k	30-F11	Biolegend	103113	1/200
CD19	Pacific Blue	Rat IgG2a, κ	6D5	Biolegend	115526	1/200
CD3ε	APC	Armenian Hamster IgG	145-2C11	Biolegend	100311	1/100
NK1.1	Brilliant Violet 650	Mouse IgG2a, κ	PK136	Biolegend	108735	1/100
CD335 (NKp46)	FITC	Rat IgG2a, κ	29A1.4	Biolegend	137606	1/200

**Antibodies**	**Fluorochrome**	**Isotype**	**Clone**	**Provider**	**Cat number**	**Dilution**
CD4	Alexa Fluor 700	Rat / IgG2a, kappa	RM4-5	eBioscience s	56-0042-82	1/200
CD8	PerCP/Cyanine5.5	Rat IgG2a, κ	53-6.7	Biolegend	100733	1/200
CD69	PE-Dazzle	Armenian Hamster IgG	H1.2F3	Biolegend	104536	1/200
CD44	PE	Rat IgG2b, κ	IM7	Biolegend	103024	1/200
CD138	Brilliant Violet 605	Rat IgG2a, κ	281-2	Biolegend	142515	1/200
CD62L	APC/Cyanine7	Rat IgG2a, κ	MEL-14	Biolegend	104428	1/200
Fixable Viability Dye	eFluor 506			eBioscience	65-0866-14	1/1000
TruStain FcX ™ (anti-mouse CD16/32)		Rat IgG2a, λ	93	Biolegend	101320	1/200

Antibodies	Fluorochrome	Isotype	Clone	Provider	Cat number	Dilution
Mixed marking Mixed extra-cell lymphoid						
CD45	Pacific Blue	Rat IgG2b, κ	S18009F	Biolegend	157212	1/200
CD11b	FITC	Rat IgG2b, κ	M1/70	Biolegend	101205	1/200
IA-IE	FITC	Rat IgG2b, κ	M5/114.15.2	Biolegend	107605	1/200
CD3ε	APC	Armenian Hamster IgG	145-2C11	Biolegend	100311	1/100
CD4	Alexa Fluor 700	Rat / IgG2a, kappa	RM4-5	eBiosciences	56-0042-82	1/200
CD8	PerCP/Cyanine5.5	Rat IgG2a, κ	53-6.7	Biolegend	100733	1/200
CD25	Brilliant Violet 650	Rat IgG1, λ	PC61	Biolegend	102037	1/200
Fixable Viability Dye	eFluor 506			eBioscience	65-0866-14	1/1000
TruStain FcX ™ (anti-mouse CD16/32)		Rat IgG2a, λ	93	Biolegend	101320	1/200
Mixed extracellular marking - Stimulation with PMA/Ionomycin						
FOXP3	PE	Mouse IgG1, κ	150D	Biolegend	320008	1/40
IL-17A	Brilliant Violet 711	Rat IgG1, κ	TC11-18H10.1	Biolegend	506941	1/40
RORgt	PE-CF594	Mouse IgG2a, κ	Q31-378	BD Biosciences	562684	1/40

Table S3 : Bacterial strains used in the study

Strain	Type	Mucoid	Reference
PAO1-WT strain	Reference strain	No	(ATCC 15692)
ΔLasB-PAO1	Laboratory strain	No	(LCB-UMR7283, CNRS, Aix Marseille Université)[22]
	Strains isolated from a cystic fibrosis (CF) patient		Ref [23]
5-6		Yes	
8-20		Yes	
7-23		No	
9-8		No	

Supplemental Fig legends

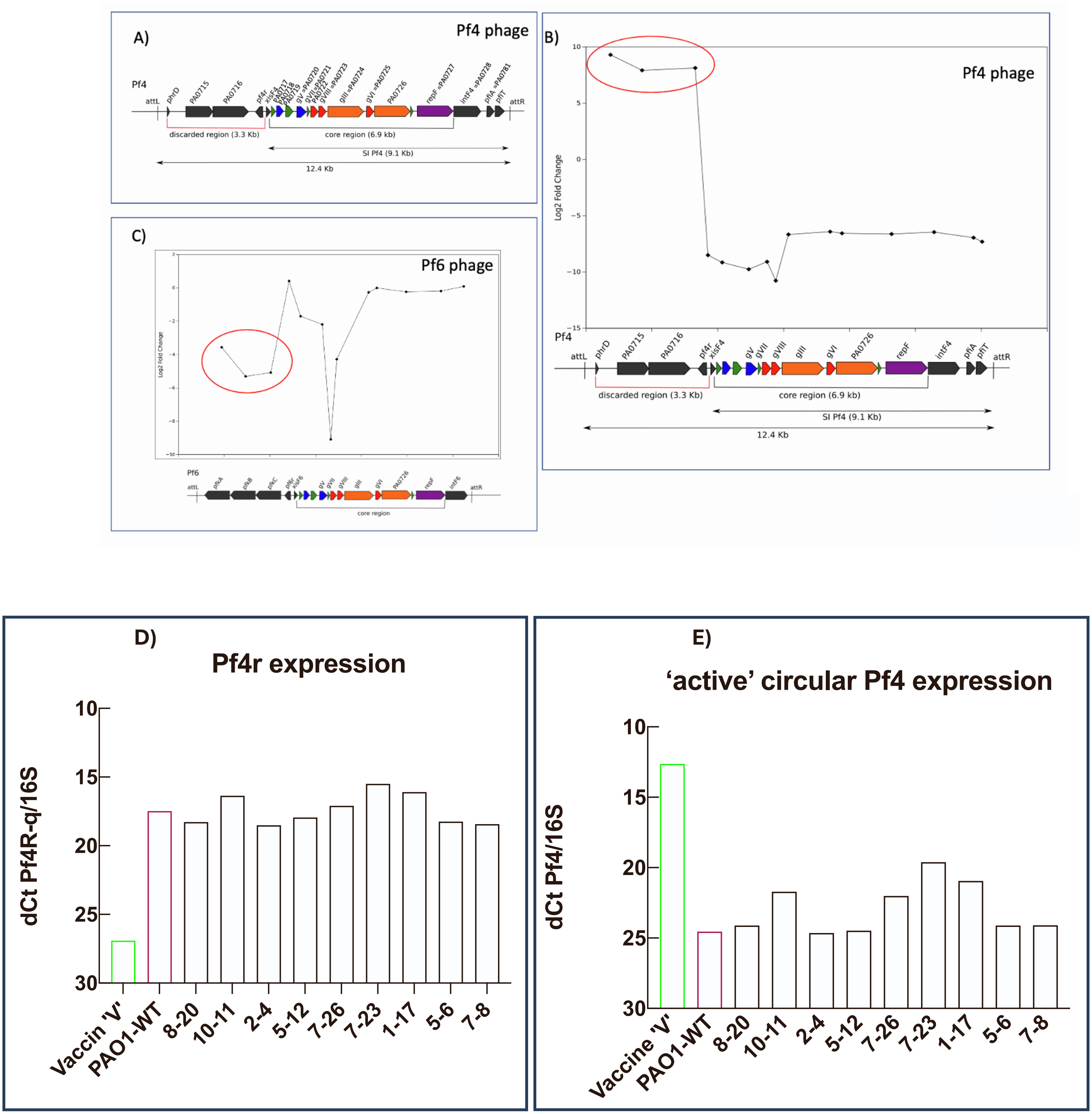

Fig S1

Fig S1:

A)Genomic organization of bacteriophage Pf4. B) RNA expression levels (RNAseq) of Pf4 genes expressed in Log2FC fold change (‘V’ = *ΔLasB*-PAO1-ASM-15d versus PAO1-WT-LB-24hrs). Genes circled (red) represent genes highly down-regulated in ‘V’, i.e *PA0715, PA0716, pf4r* from the discarded region (DR). C) Genomic organization of bacteriophage Pf6 and RNA expression (RNAseq) of Pf6 genes expressed in fold change as above (‘V’ = *ΔLasB*-PAO1-ASM-15d versus PAO1-WT-LB-24hrs). Genes circled in red represent genes up-regulated in ‘V’, i.e *pfkA, pfkB, pfkC,* genes forming a regulatory module controlling virion production of the co-resident Pf4 prophage[34]. **D)** The expression of the repressor r gene of Pf4 was analysed in PAO1 WT, ‘V’, and several CF clinical strains, by q-PCR amplifying a 206pb fragment in the R2 region of DR (discarded region). NB : the highest the dCT, the lower the level of expression (Pf4r-qF : AGTTTGGCCCGACTTCTGGG ; Pf4r-qR : CGTCAGCCCGATAGAGCAAGA). **E)** q-PCR analysis of the circularization site of Pf4 (attP), located between the integrase gene and the repressor genes was performed (Pf4 FWD : TAGGCATTTTCAGGGGGCTTGG ; Pf4 REV : GAGCTACGGAGTAAGACGCC), amplifiying a 135 bp fragment. This allowed for the detection of only the circularized Pf4 DNA and not the linear Pf4 prophage sequence. NB : the highest the dCT, the lower the level of expression.

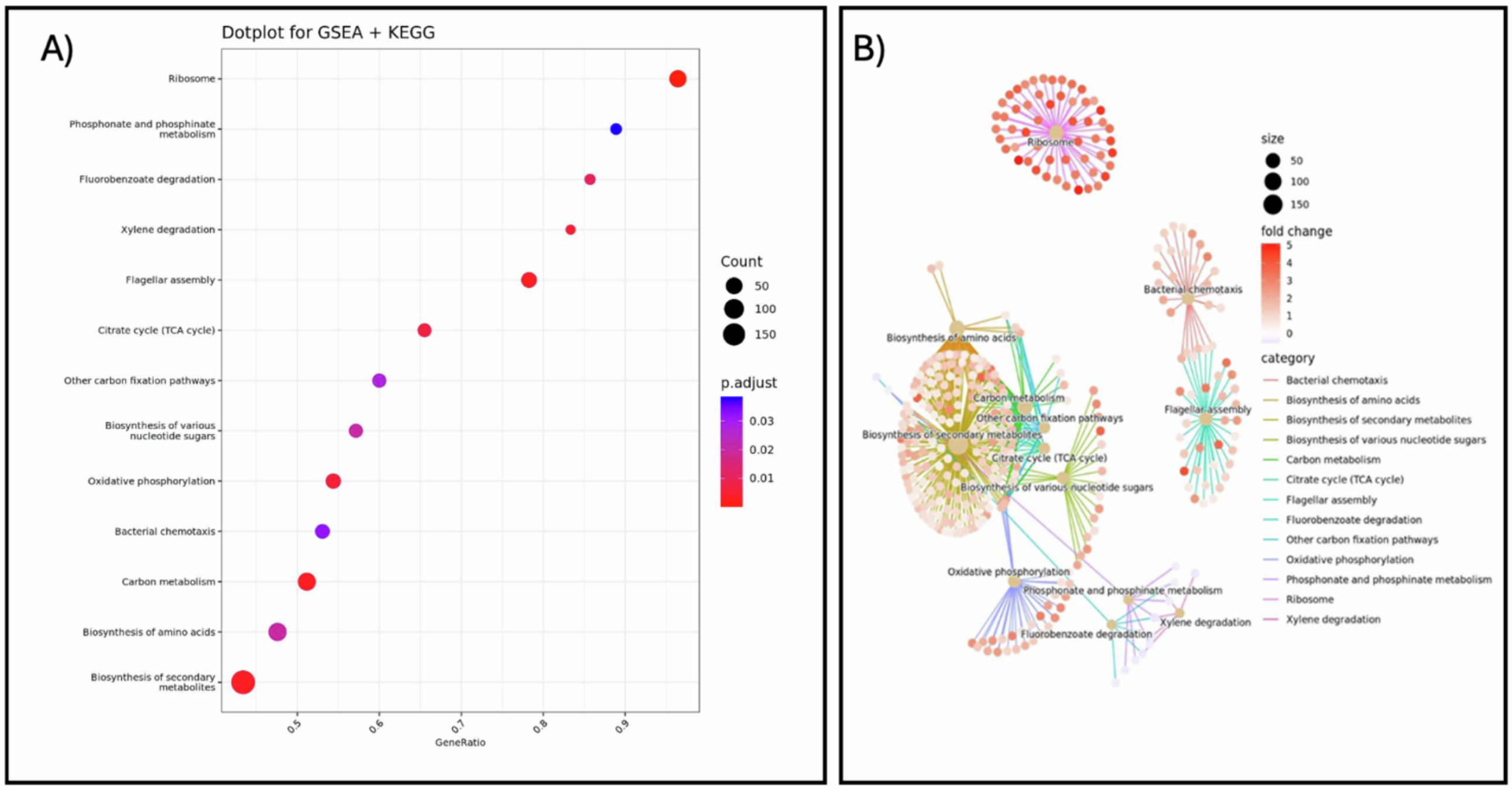

Fig S2

FigS2: A) KEGG pathway enrichment analysis of differentially expressed genes. Gene Set Enrichment Analysis (GSEA) was performed using the Kyoto Encyclopedia of Genes and Genomes (KEGG) database to identify significantly enriched biological pathways. The dot plot displays the 13 KEGG terms with the highest enrichment scores. Each dot represents a pathway, with the term names shown on the left of the plot. The gene ratio (proportion of input genes associated with each pathway) is indicated on the x-axis, ranging from 0.4 to 1.0**.** Dot size reflects the number of genes contributing to each pathway, and color intensity corresponds to the adjusted p-value (FDR)**,** with darker colors indicating higher statistical significance. B) : KEGG Gene-concept network

Shown are associations between differentially expressed genes (DEGs) and significantly enriched KEGG pathways identified in the RNA-seq dataset. Each large node represents an enriched KEGG term (e.g., *Ribosome*, *Flagellar assembly…*), with node size proportional to the number of DEGs mapped to that pathway. Small nodes correspond to individual DEGs, colour-coded according to

their $\log_2$ fold change (red, upregulated; blue, downregulated). Edges connect genes to their associated KEGG pathways and indicate shared functional relationships.

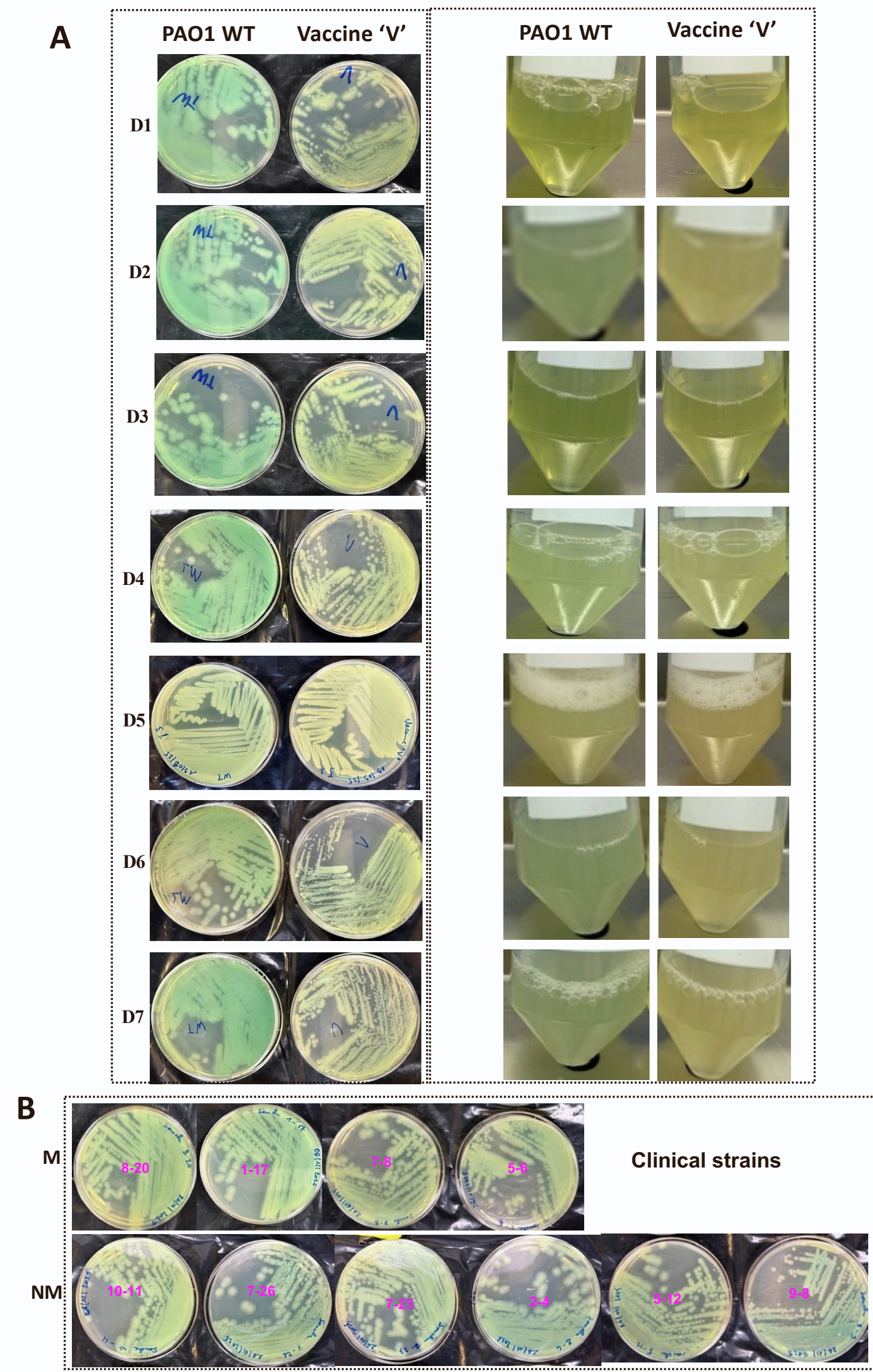

C

Strains	PAO1 WT	Vaccine 'V'	5-6	7-8	7-23	8-20	9-8	2-4	5-12	7-26	10-11	1-17
Colony morphology	Large	Small	Large	Large	Large	Large	Small	Small	Small	Large	Small	Large
Auxotrophy	No	Yes	No	No	No	Yes	No	No	No	Yes	Yes	No
Mucoid	-	-	Yes	Yes	No	Yes	No	No	No	No	No	Yes

Fig S3

Fig S3 :
(A) Phenotypic characterization of WT and vaccine strains, colony appearance after successive subculturing for 7 days.
(**B**) Phenotypic characterization of 10 clinical strains (M=Mucoid, NM=Non-mucoid). (**C**) Microbiological characterization of strains (colony morphology, small/large ; auxotrophy ; mucoid status)

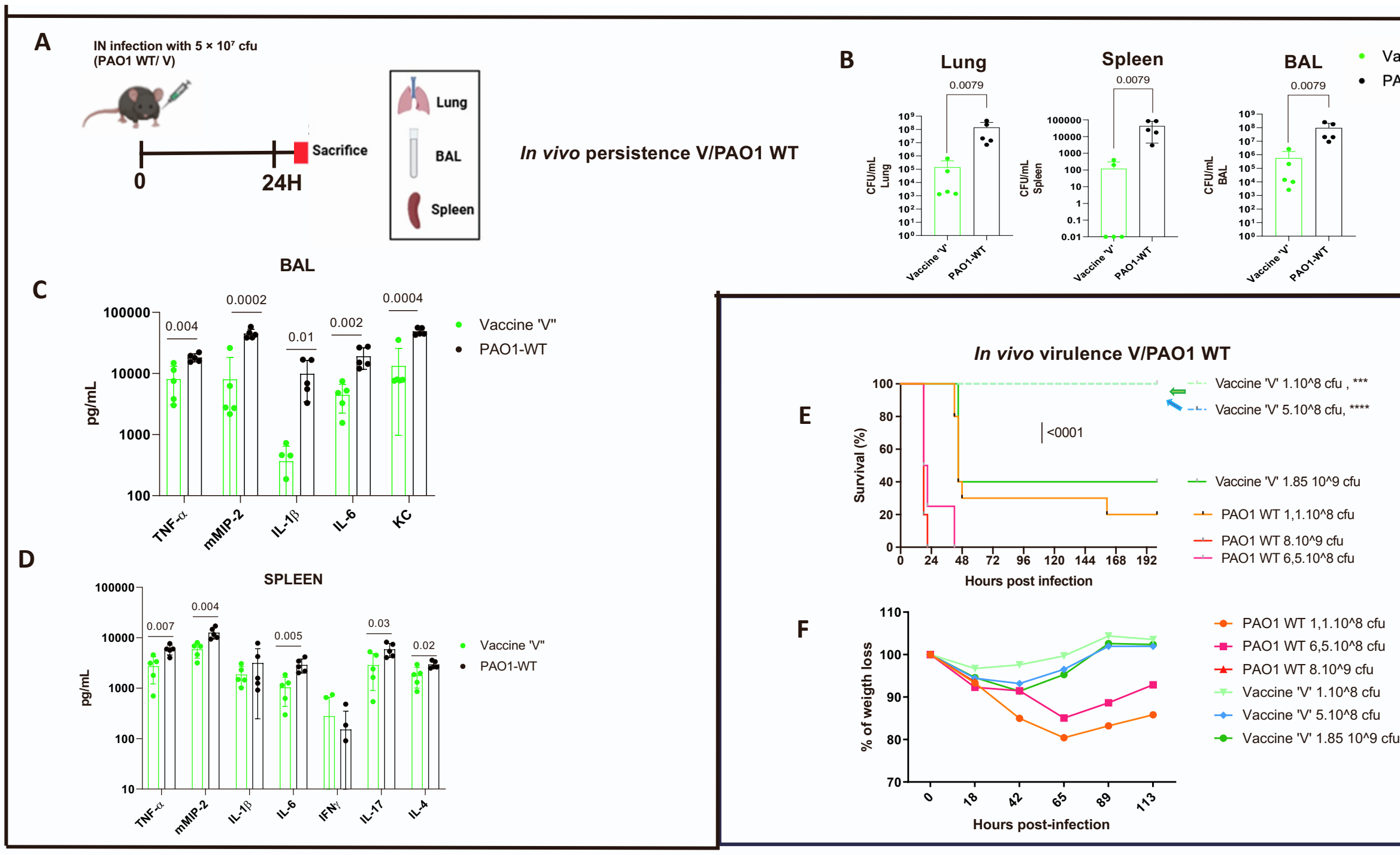

Fig S4

Fig S4 *In vivo* persistence and virulence of PAO1-WT and V

A-D) C57/Bl6 mice were infected i.n with 5 x 10^7 cfu of either PAO1-WT or V (A). 24 hrs later, animals were sacrificed, and lungs, BALs and spleen were recovered for CFU assessment (B). Cytokine levels were also measured by ELISA in BALs (C) or spleen (D)

E-F) In independent experiments, mice were similarly infected with various doses of PAO1-WT or V and survival (panel E) and weights (panel D) were followed over time. Survival (Kaplan-Meier representation was plotted and Log-Rank (Mantel-Cox) tests performed : *** (p=0.0005) : V 1.10^8 pfu/PAO1 WT ; **** (p<0. 0001) : V 5.10^8 pfu/PAO1 WT. NB : in E), the survival of the 6 mice is reported ; in F), the weight of the 6 mice is reported for 0 to 18 hrs, but only that of 5 from 18 to 113 hrs (the mice infected with PAO1 WT 8.10^9 cfu were dead at 18hrs). Mann-Whitney test (A), multiple t-tests comparisons (B-C) were used (p<0.05). Error bars represent standard deviations (SD).

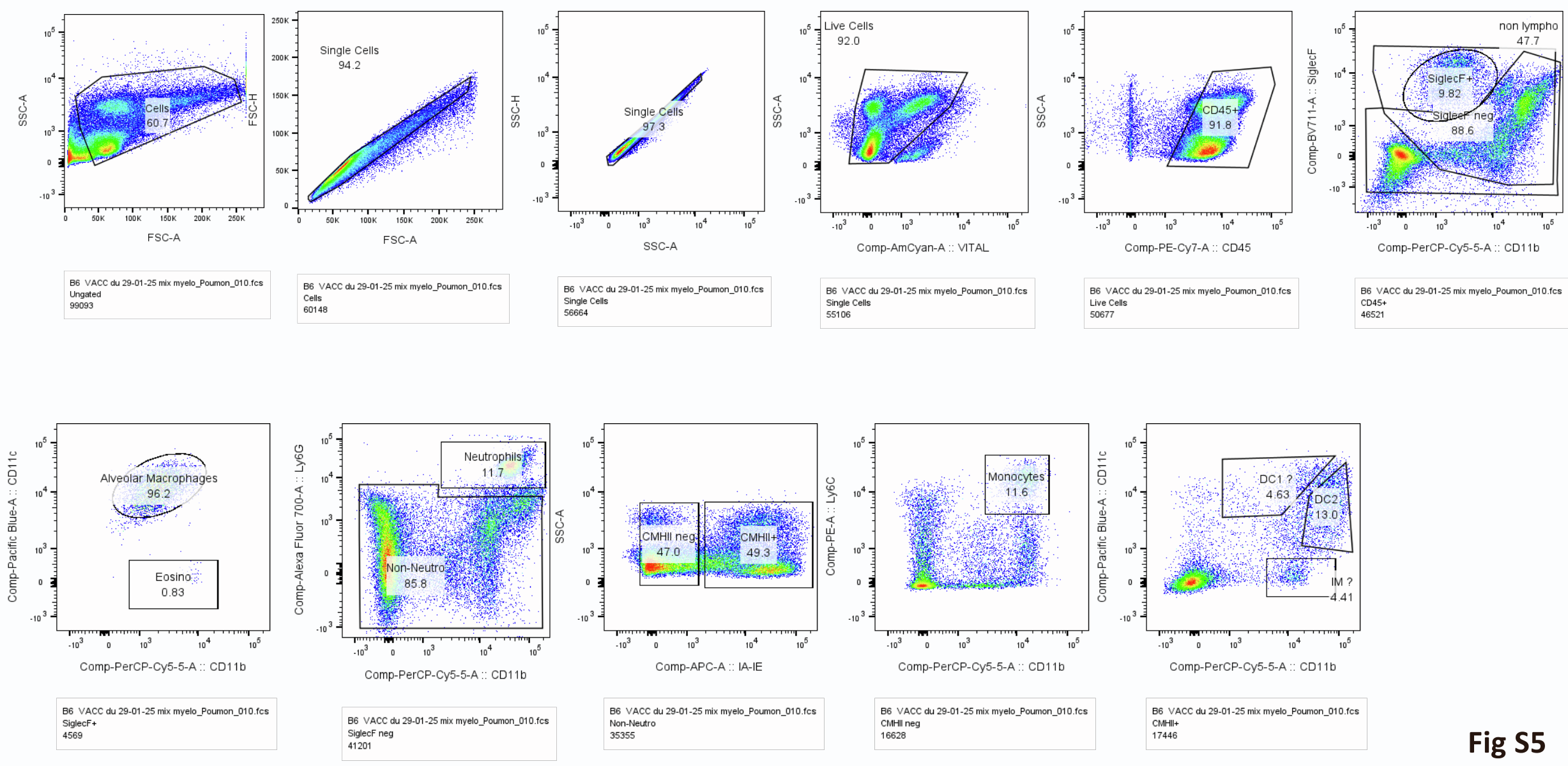

Fig S5

Fig S5 : LUNG – MIX MYELOID GATING STRATEGY

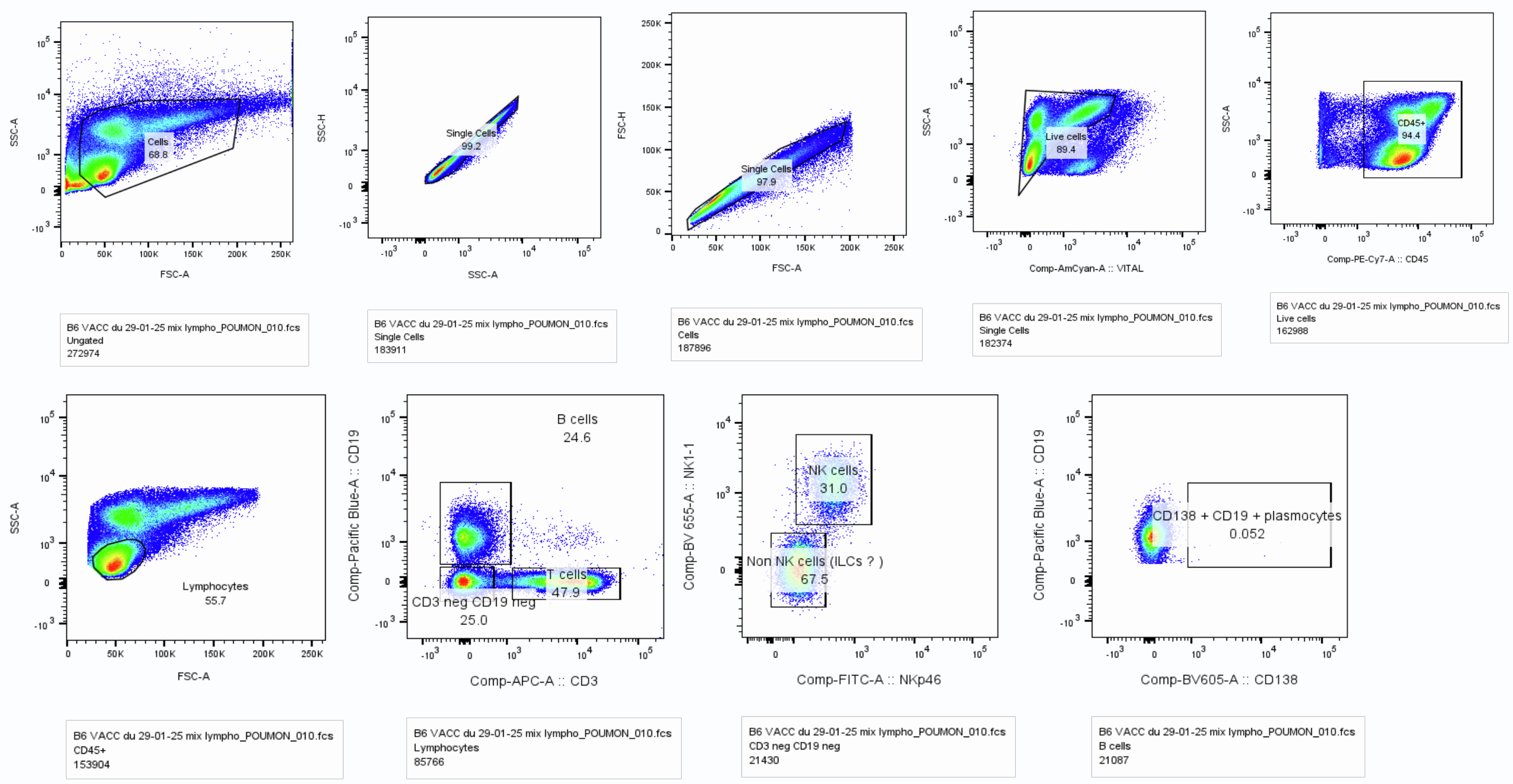

Fig S6

Fig S6 : LUNG – Mix LYMPHOID GATING STRATEGY

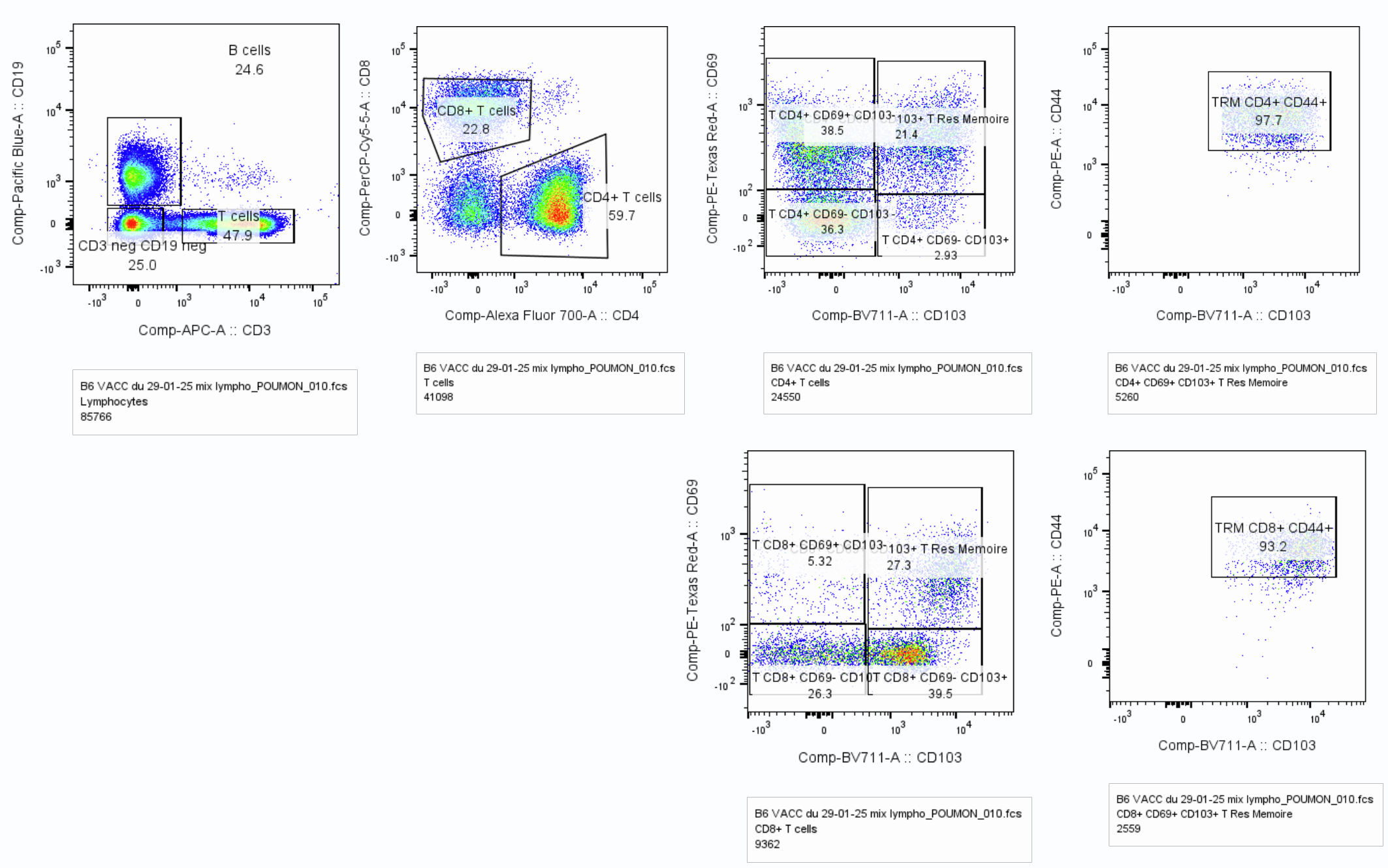

Fig S7

Fig S7 : LUNG – Mix LYMPHOID TRM GATING STRATEGY

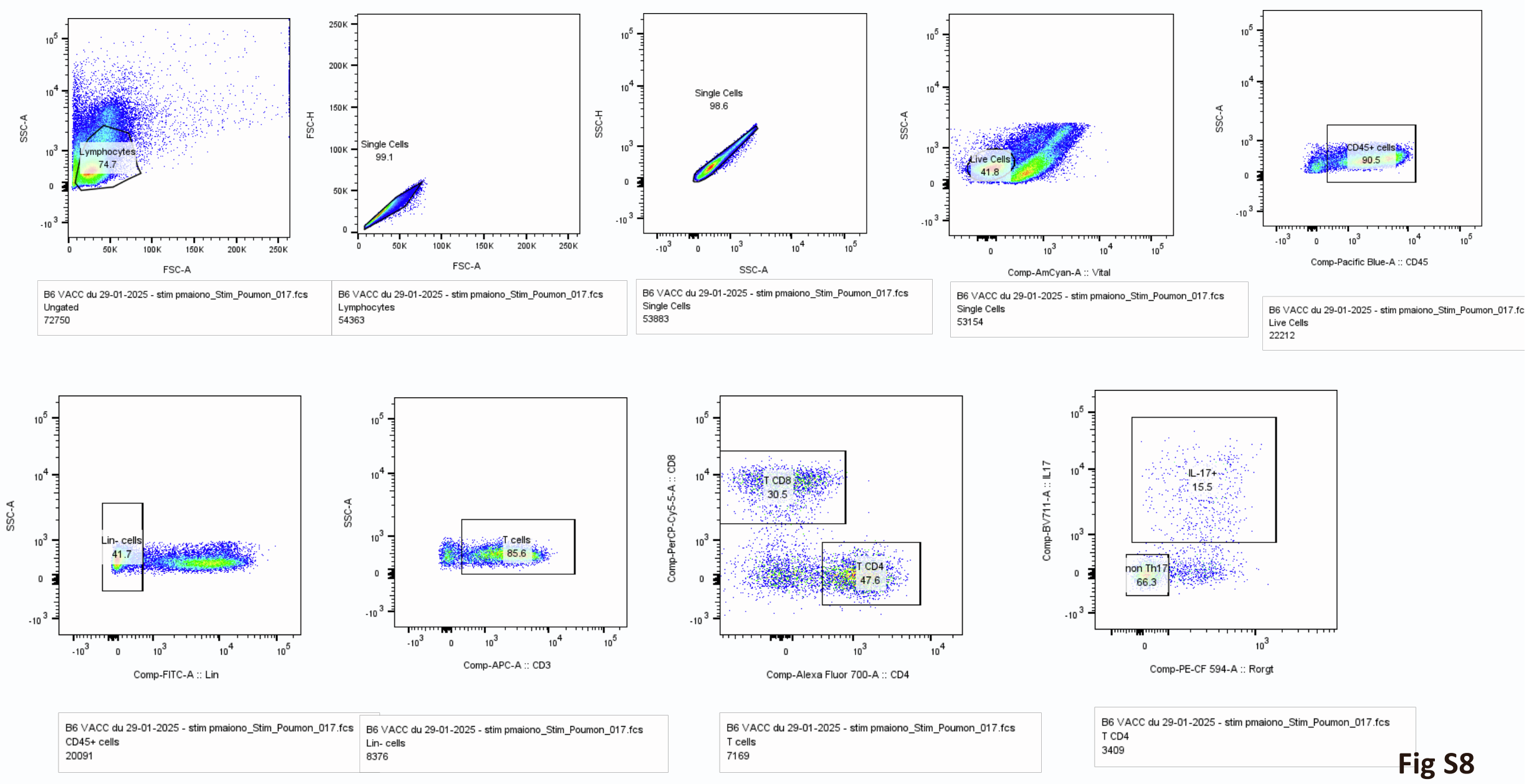

Fig S8

Fig S8 : LUNG – MIX INTRA-CELL – STIM PMA/IONO

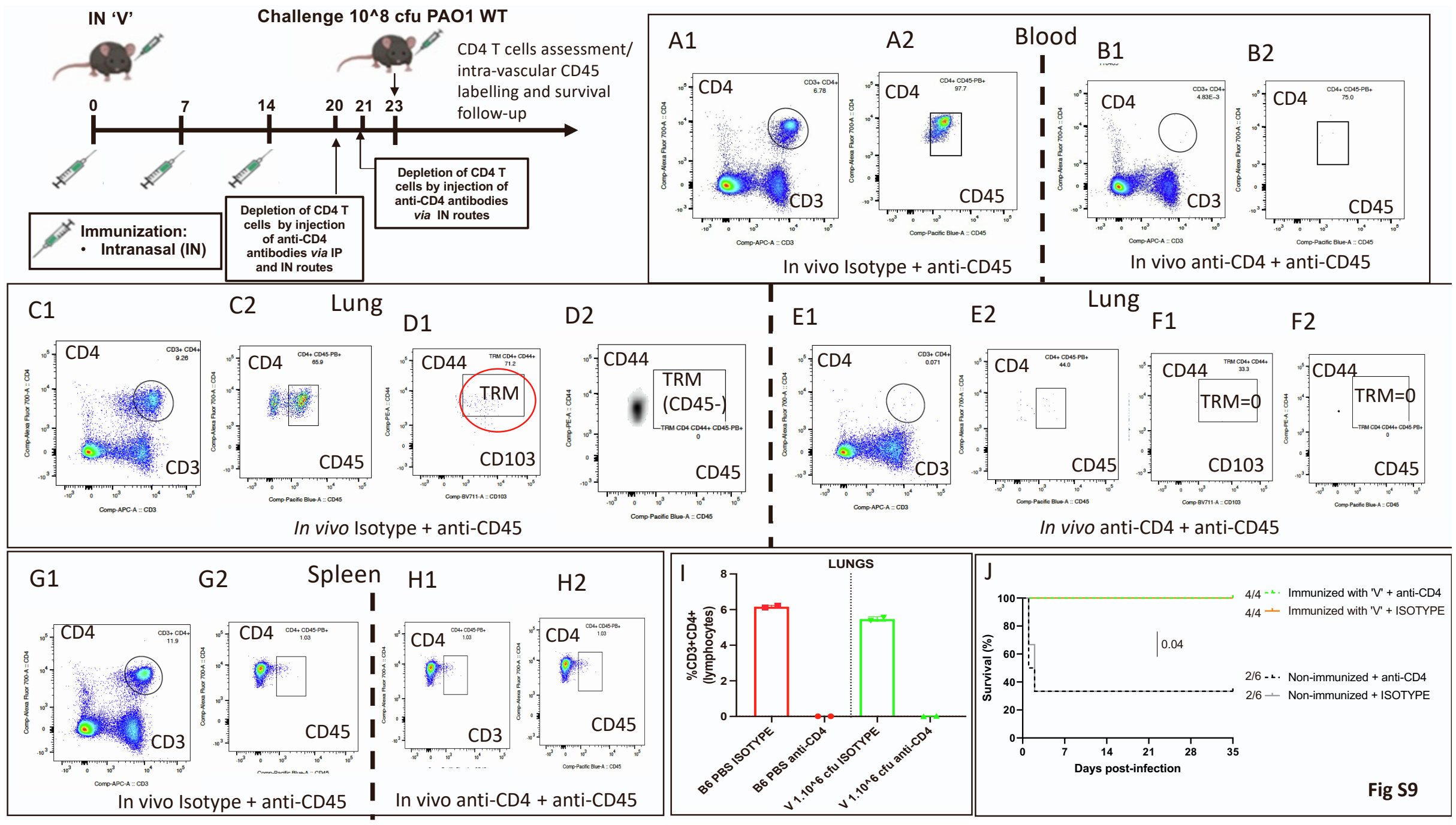

Fig S9 : Depletion of CD4 T cells, vaccination with 'V' and challenge with PAO1-WT

Mice were mock-vaccinated with PBS or with 'V' as in Fig 6/Fig S13 until day 14. At day 20, they were injected i.p with 250 ug of anti-CD4 antibody (or isotype) and i.n with 30 ugs of the same antibodies. At day 21, this was repeated i.n with 30 ugs of antibodies.

At day 23, 2 mice from each group were anaesthetized, injected retro-orbitally with 2 ugs of anti-CD45-PB for intra-vascular labelling. 3 min later, these mice were sacrificed, spleen and blood retrieved, as well as lungs (after perfusion) for assessment of CD4 T cells depletion and CD45 labelling (panels A-I)

The remainder mice in each group were challenged with a lethal dose of PAO1-WT (5.10^8 cfu) and survival was assessed (panel J). Survival (Log-Rank (Mantel-Cox) tests was used: * (p=0.04).

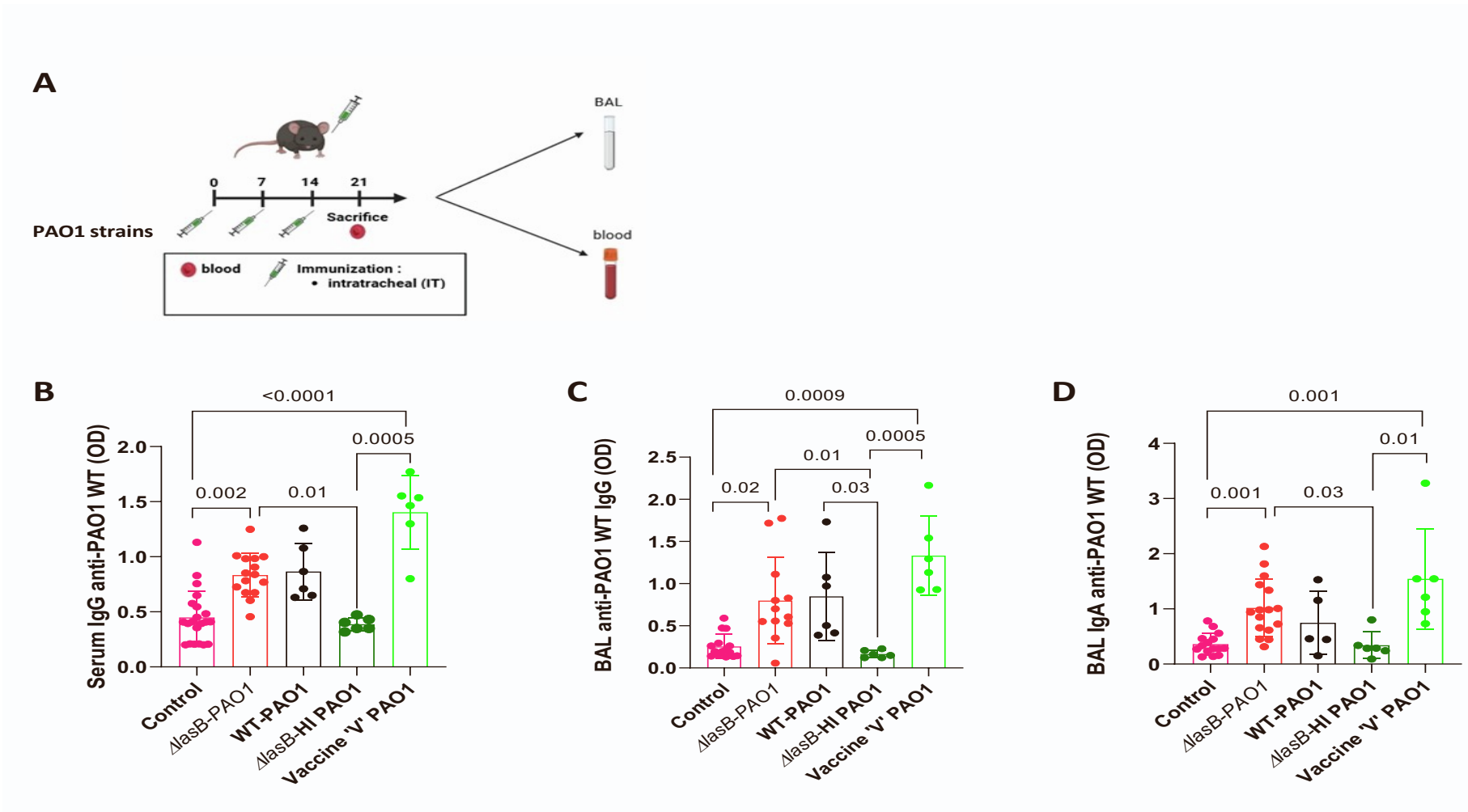

Fig S10

Fig. S10: **Comparative immunogenicity (humoral responses) of PAO1-WT-LB-24hrs, *ΔLasB*-PAO1-LB-24hrs, heat-inactivated (HI) *ΔLasB*-PAO1-24hrs (*ΔLasB*-PAO1-HI-24hrs), *ΔLasB*-PAO1-ASM-15days =‘V’**

A) C57BL/6 male mice were given IT (at days 0, 7, 14) either PBS (control) or were immunized (same time points) with either 10^6CFU of live *ΔLasB*-PAO1, live PAO1-WT, *ΔLasB*-HI PAO1or live vaccine ‘V’. At day 21, 7 days after the last boost, blood and BALs were collected and used for anti-PAO1-WT serum total IgG **(B)**, anti-PAO1-WT BAL total IgG **(C)**, anti-PAO1-WT BAL IgA **(D)**. Kruskal-Wallis Multiple comparison test was used for to determine significance p<0.05. Error bars represent standard deviations (SD).

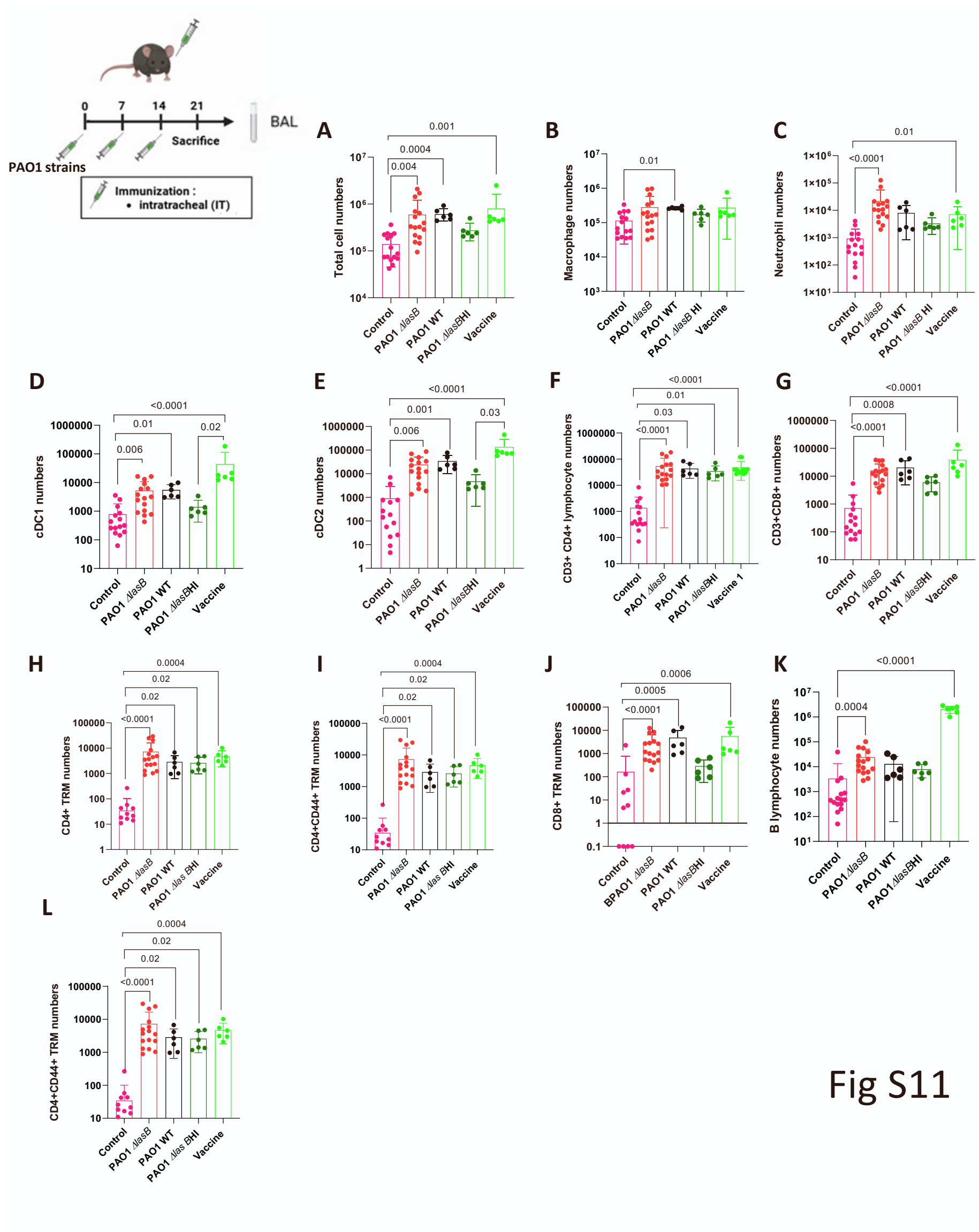

Fig S11: **Comparative immunogenicity (BAL cellular content) of PAO1-WT-LB-24hrs, *ΔlasB*-PAO1-LB-24hrs, heat-inactivated (HI) *ΔLasB*-PAO1-24hrs (*ΔLasB*-PAO1-HI-24hrs), *ΔLasB*-PAO1-ASM-15days ='V'**

BAL cells (see Fig S10) were recovered at d21 and analysed by FACS. Total cells, myeloid and lymphoid cells were quantified and levels plotted in panels A-L. Kruskal-Wallis Multiple comparison test was used to determine significance $p<0.05$. Error bars represent standard deviations (SD).

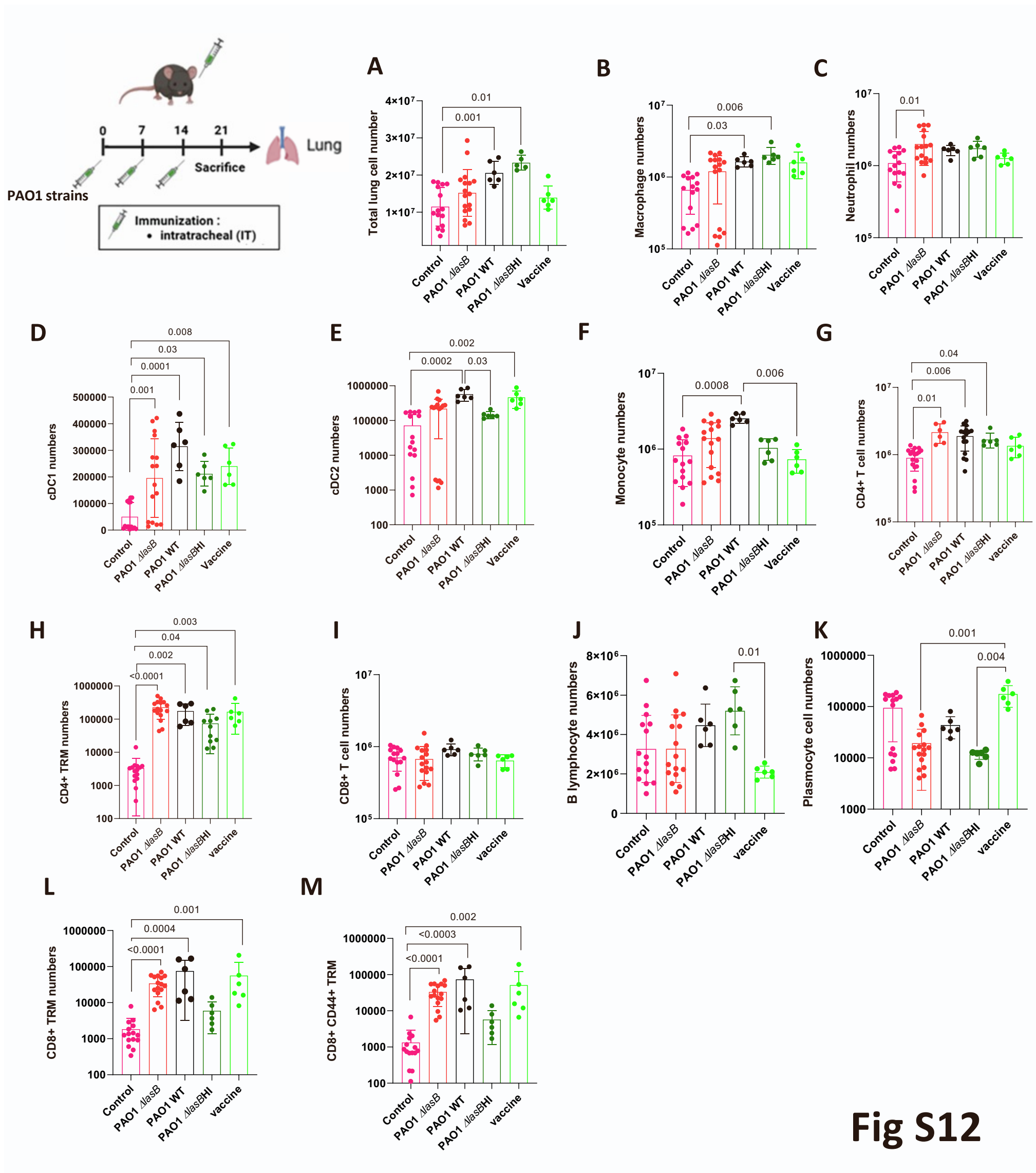

Fig S12: **Comparative immunogenicity (lung cellular content) of PAO1-WT-LB-24hrs, *ΔLasB*-PAO1-LB-24hrs, heat-inactivated (HI) *ΔLasB*-PAO1-24hrs (*ΔLasB*-PAO1-HI-24hrs), *ΔLasB*-PAO1-ASM-15days ='V'**

Lung cells **(see Fig S10)** were recovered at d21 and analysed by flow cytometry

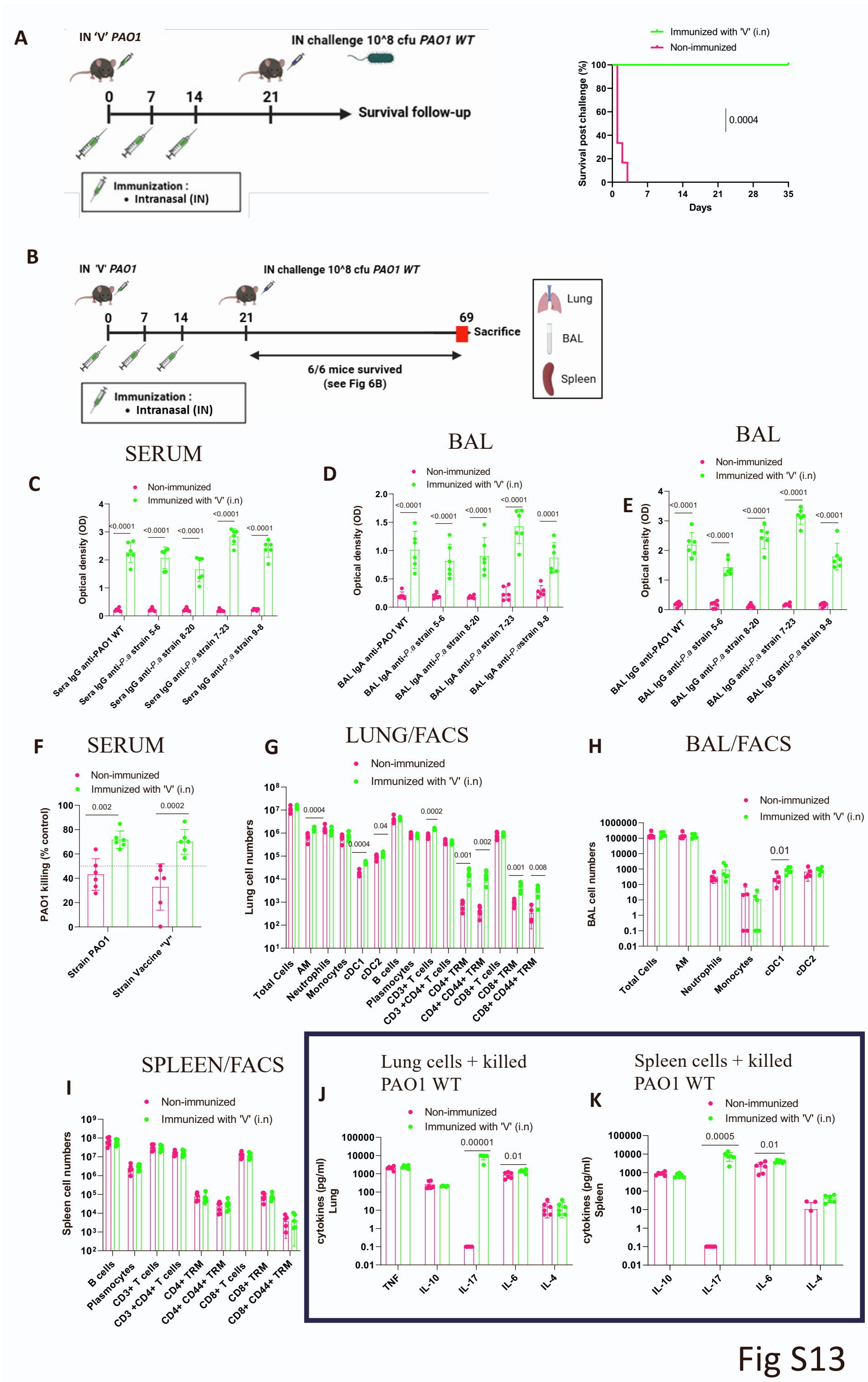

Fig S13: Challenge and assessment of 'intermediate' memory responses following i.n immunization with 'V' and re-challenge with PAO1-WT

A) Mice were immunized as above, but intra-nasally (i.n), instead of i.t. 7 days after the last boost, male C57BL/6 mice (n=6) were challenged with a lethal dose of 10^8 CFU of PAO1-WT and survival was monitored. The p-value indicates significant differences in a Mantel-Haenszel log-rank test.

(B) Vaccinated mice which survived the challenge in **A)** were observed until day 69 (i.e 48 days post-challenge) when antibodies levels were measured :

(**C, D** and **E**) : serum anti-PAO1-WT (and anti-P.a clinical strains mucoid/5-6; 8-20; and non-mucoid/7-23;9-8) total IgG ; BAL anti-PAO1-WT (and anti-P.a clinical strains) total IgG; BAL anti-PAO1-WT (and anti-P.a clinical strains) IgA. **(F)** Rabbit Complement-dependent killing of PAO1-WT and 'V' (with 1/2 dilutions of sera from 'V'-immunized and non-immunized mice (n = 6)). **(G)** Day 69 FACS analysis of lung B lymphocytes, plasmocytes, alveolar macrophages (AM), neutrophils, monocytes, dendritic cells (cDC1 and cDC2), CD3+ T, CD3+CD4+ T, CD3+CD8+ T, CD4+ TRM, CD8+ TRM, CD4+CD44+TRM, CD8+CD44+ TRM, CD8+TRM and CD8+CD44+TRM T cells. NB : TRM are defined as CD3+CD69+CD103+ T cells. **H-I)** : Day 69 BAL and spleen FACS analysis of myeloid and lymphoid cells (see markers above). **J-K) :** At day 69, following the same protocol as in Fig 5 and Fig 8, lung **(J)** and spleen (**K)** were harvested and 10^6 cells were incubated with gentamycin-killed bacteria (MOI =1) during 48hrs**.** TNF, IL-10, IL17, IL-6 and IL-4 cytokines levels were then measured (ELISA) in cell supernatants . Error bars represent standard deviations. Two way ANOVA comparaison was used to determine significance $p<0.05$ for (C, D, E and F). Multiple t tests with was used for (G, H, I, K) to determine significance $p<0.05$. Error bars represent standard deviations (SD).

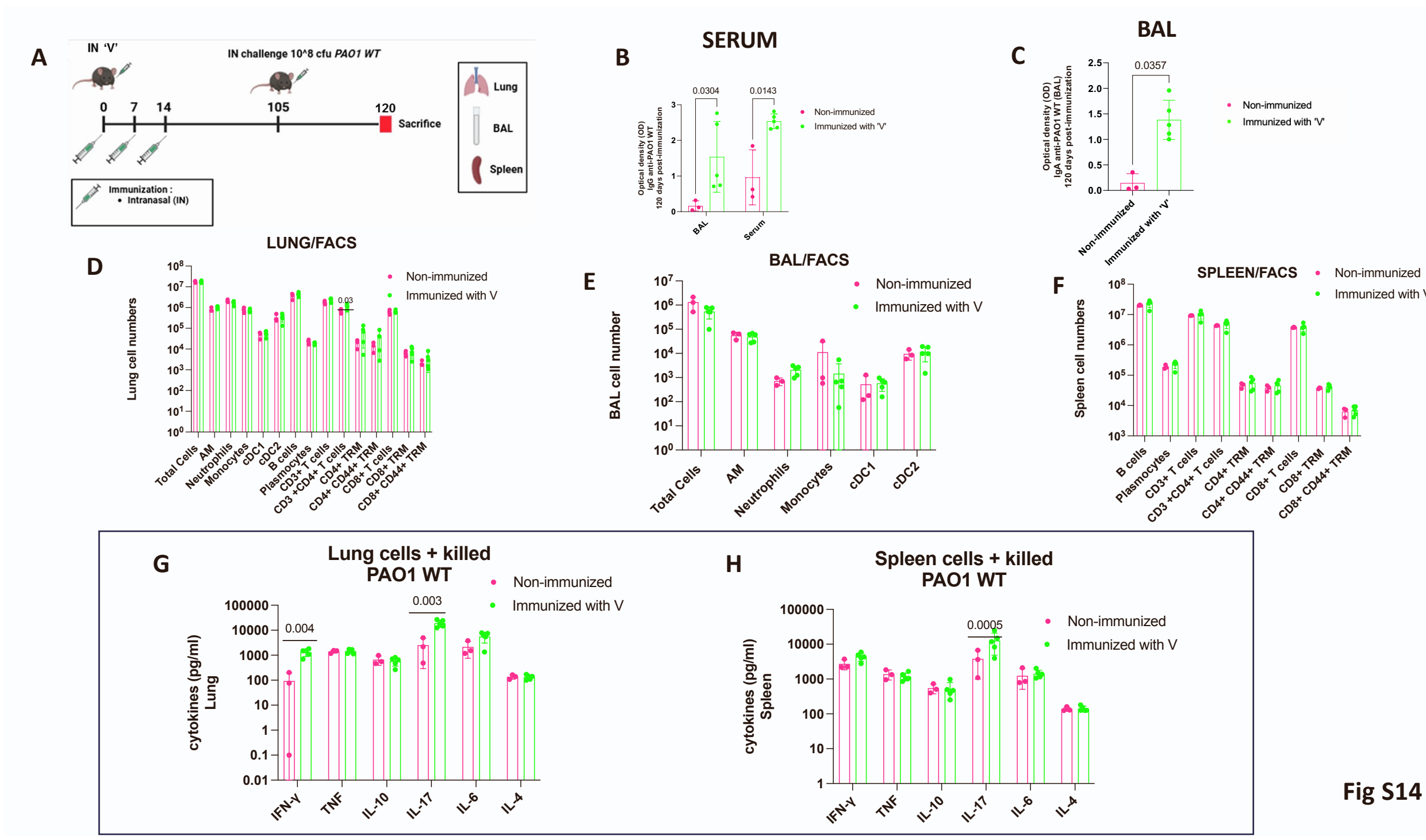

Fig S14 : Assessment of 'long term' memory responses following i.n immunization with 'V' and re-challenge with PAO1-WT 105 days after the last boost.

A) Mice (male C57BL/6, n=6) were immunized with 'V' or mock-immunised with PBS as above. 105 days after the last boost, mice were challenged with a sub-lethal dose of 10^7 CFU of PAO1-WT. Seven days later, antibodies levels anti-PAO1 WT were measured in serum and BAL (panels B-C). Lung, BAL and spleen flow cytometry analysis of lung B lymphocytes, plasmocytes, alveolar macrophages (AM), neutrophils, monocytes, dendritic cells (cDC1 and cDC2), CD3+ T, CD3+CD4+ T, CD3+CD8+ T, CD4+ TRM, CD8+ TRM, CD4+CD44+TRM, CD8+CD44+ TRM, CD8+TRM and CD8+CD44+TRM T cells is shown in panels D-F. NB: TRM are defined as CD3+CD69+CD103+ T cells. (panels D-F). At day 120, following the same protocol as in Fig 5 and Fig 8, lung (panel G) and spleen (panel H) were harvested and 10^6 cells were incubated

with gentamycin-killed bacteria (MOI =1) during 48hrs**.** TNF, IL-10, IL17, IL-6 and IL-4 cytokines levels were then measured (ELISA) in cell supernatants. Two way ANOVA (A), Mann-Whitney test (B) and t-tests multiple comparisons were performed (C, D, E, F, G and H) ($p<0.05$). Error bars represent standard deviations (SD).

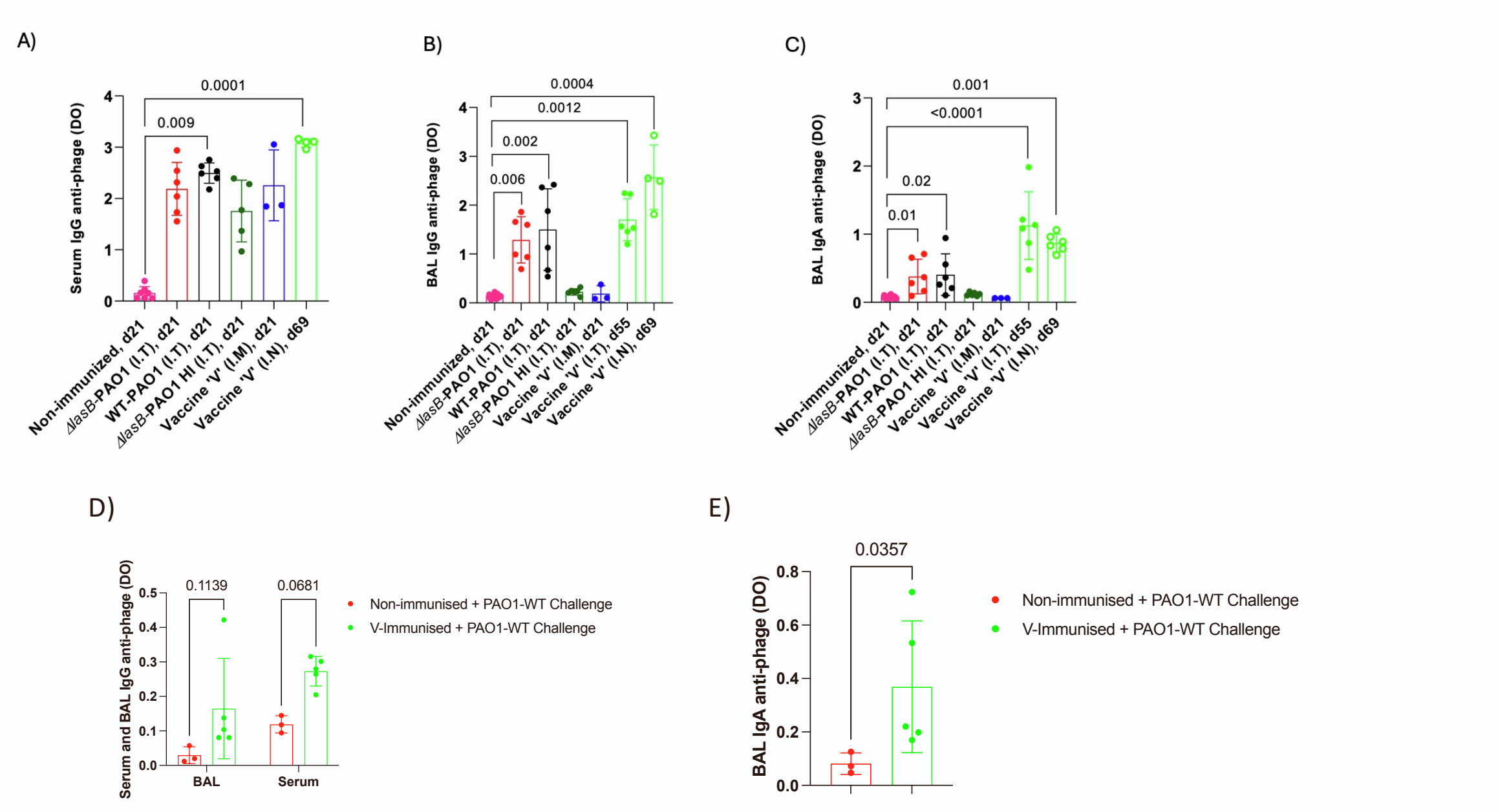

Fig S15

Fig S15: Detection of anti-phage antibodies in sera and BALs from immunized mice

A) C57BL/6 male were either non-immunized or immunized at days 0, 7, 14 either i.t, i.m or i.n, with either 10^6CFU of live *ΔLasB*-PAO1, live PAO1-WT, *ΔLasB*-PAO1-HI (heat-inactivated) or with the vaccine 'V' PAO1 strain. At either day 21, 55 or 69 (see corresponding Figures), blood and BALs were collected and used for assessment of anti-phage serum total IgG **(A)**, anti-phage BAL total IgG **(B)**, or anti-phage BAL IgA **(C)**. Kruskal-Wallis Multiple comparaison test was used to determine significance p<0.05. Error bars represent standard deviation.

D-E) C57BL/6 male were either mock-PBS-immunized or V-immunized i.n at days 0, 7, 14 with live 10^6 CFU. At day 105, all mice were further challenged with 10^7 cfu of PAO1 WT. 15 days later, mice were sacrificed, and anti-phage antibodies were assessed in sera (D) and BALs (E). Kruskal-Wallis Multiple comparaison test was used to determine significance (p<0.05). Error bars represent standard deviation.

Supplemental references

1. Born-Bony M, Cornu C, Villeret B, Gratio V, Voulhoux R, Sallenave JM. Intrapulmonary-administered myeloid derived suppressor cells rescue mice from Pseudomonas aeruginosa infection and promote a regulatory/repair phenotype. Mucosal Immunol. juin 2025;18(3):700‑16.

2. Holloway BW. Genetic Recombination in Pseudomonas aeruginosa. Microbiology. 1955;13(3):572‑81.

3. Kaniga K, Delor I, Cornelis GR. A wide-host-range suicide vector for improving reverse genetics in Gram-negative bacteria: inactivation of the *blaA* gene of *Yersinia enterocolitica*. Gene. 20 déc 1991;109(1):137‑41.

4. Patel H, Manning J, Ewels P, Garcia MU, Peltzer A, Hammarén R, et al. nf-core/rnaseq: nf-core/rnaseq v3.19.0 - Tungsten Turtle [Internet]. Zenodo; 2025 [cité 6 oct 2025]. Disponible sur: https://zenodo.org/records/15631172

5. Bray NL, Pimentel H, Melsted P, Pachter L. Near-optimal probabilistic RNA-seq quantification. Nat Biotechnol. mai 2016;34(5):525‑7.

6. Love MI, Huber W, Anders S. Moderated estimation of fold change and dispersion for RNA-seq data with DESeq2. Genome Biol. 5 déc 2014;15(12):550.

7. Wu W, Huang J, Duan B, Traficante DC, Hong H, Risech M, et al. Th17-stimulating protein vaccines confer protection against Pseudomonas aeruginosa pneumonia. Am J Respir Crit Care Med. 1 sept 2012;186(5):420‑7.

8. Wick RR, Judd LM, Gorrie CL, Holt KE. Completing bacterial genome assemblies with multiplex MinION sequencing. Microb Genomics. oct 2017;3(10):e000132.

9. De Coster W, D'Hert S, Schultz DT, Cruts M, Van Broeckhoven C. NanoPack: visualizing and processing long-read sequencing data. Bioinforma Oxf Engl. 1 août 2018;34(15):2666‑9.

10. Li H. Minimap2: pairwise alignment for nucleotide sequences. Bioinforma Oxf Engl. 15 sept 2018;34(18):3094‑100.

11. Kolmogorov M, Yuan J, Lin Y, Pevzner PA. Assembly of long, error-prone reads using repeat graphs. Nat Biotechnol. mai 2019;37(5):540‑6.

12. Marçais G, Delcher AL, Phillippy AM, Coston R, Salzberg SL, Zimin A. MUMmer4: A fast and versatile genome alignment system. PLoS Comput Biol. janv 2018;14(1):e1005944.

13. Robinson JT, Thorvaldsdóttir H, Winckler W, Guttman M, Lander ES, Getz G, et al. Integrative Genomics Viewer. Nat Biotechnol. janv 2011;29(1):24‑6.

14. Tatusova T, DiCuccio M, Badretdin A, Chetvernin V, Nawrocki EP, Zaslavsky L, et al. NCBI prokaryotic genome annotation pipeline. Nucleic Acids Res. 19 août 2016;44(14):6614‑24.

15. Sereme Y, Schrimp C, Faury H, Agapoff M, Lefebvre-Wloszczowski E, Chang Marchand Y, et al. A live attenuated vaccine to prevent severe neonatal Escherichia coli K1 infections. Nat Commun. 8 avr 2024;15(1):3021.

16. O'Loughlin CT, Miller LC, Siryaporn A, Drescher K, Semmelhack MF, Bassler BL. A quorum-sensing inhibitor blocks Pseudomonas aeruginosa virulence and biofilm formation. Proc Natl Acad Sci U S A . 2013 Oct 29;110(44):17981-6.

17. Schlitzer A, Sivakamasundari V, Chen J, Sumatoh HRB, Schreuder J, Lum J, et al. Identification of cDC1- and cDC2-committed DC progenitors reveals early lineage priming at the common DC progenitor stage in the bone marrow. Nat Immunol. juill 2015;16(7):718‑28.

18. Fejer G, Wegner MD, Györy I, Cohen I, Engelhard P, Voronov E, et al. Nontransformed, GM-CSF-dependent macrophage lines are a unique model to study tissue macrophage functions. Proc Natl Acad Sci U S A. 11 juin 2013;110(24):E2191-2198.

19. Ramsay PL, Luo Z, Magdaleno SM, Whitbourne SK, Cao X, Park MS, et al. Transcriptional regulation of CCSP by interferon-γ in vitro and in vivo. Am J Physiol-Lung Cell Mol Physiol. 1 janv 2003;284(1):L108‑18.

20. Granoff DM. Relative importance of complement-mediated bactericidal and opsonic activity for protection against meningococcal disease. Vaccine. 24 juin 2009;27:B117‑25.

21. Sweere JM, Van Belleghem JD, Ishak H, Bach MS, Popescu M, Sunkari V, et al. Bacteriophage trigger antiviral immunity and prevent clearance of bacterial infection. Science. 29 mars 2019;363(6434):eaat9691.

22. Toder DS, Ferrell SJ, Nezezon JL, Rust L, Iglewski BH. lasA and lasB genes of Pseudomonas aeruginosa: analysis of transcription and gene product activity. Infect Immun. avr 1994;62(4):1320-7.

23. Llanes C, Pourcel C, Richardot C, Plésiat P, Fichant G, Cavallo JD, et al. Diversity of β-lactam resistance mechanisms in cystic fibrosis isolates of Pseudomonas aeruginosa: a French multicentre study. J Antimicrob Chemother. 1 août 2013;68(8):1763-71.
