# Supplementary material for: A novel cystic fibrosis-mimetic Pseudomonas auxotrophic vaccine is protective in vivo and is associated with IL-17/IgA mucosal Immunity
Source: Mol Ther. 2026 Jun 17;34(9):5302–18. doi: 10.1016/j.ymthe.2026.06.029 (PMC13555540; doi:10.1016/j.ymthe.2026.06.029)
Supplement: Supplementary file 2 [file mmc3.pdf]

**Molecular Therapy**
Original Article

# A novel cystic fibrosis-mimetic *Pseudomonas* auxotrophic vaccine is protective *in vivo* and is associated with IL-17/IgA mucosal Immunity

Youssouf Sereme,[1,2,8] Bérengère Villeret,[1,2,8] Ségolène Caboche,[3] Samuel Ikeh,[1,2] Hugo Leroy,[1] Delphine Beury,[3] Florence Maurier,[3] Christophe Desterke,[4] Maëlys Born-Bony,[1,2] Zhou Xing,[5,6] Romé Voulhoux,[7] and Jean-Michel Sallenave[1,2]

[1]Institut National de la Santé et de la Recherche Médicale, U1149 (Centre de Recherche sur l'Inflammation), Université Paris-Cité, 16 Rue Henri Huchard, 75018 Paris, France; [2]Institut National de la Santé et de la Recherche Médicale, U1152 (Physiopathologie et Epidémiologie des Maladies Respiratoires), Université Paris-Cité, 16 Rue Henri Huchard, 75018 Paris, France; [3]Université de Lille, CNRS, Inserm, CHU Lille, Institut Pasteur de Lille, US41-UAR 2014, 59000 Lille, France; [4]University Paris Saclay, Faculty of Medicine, INSERM UMRS-1310, Villejuif, France; [5]McMaster Immunology Research Centre, McMaster University, Hamilton, ON, Canada; [6]Department of Medicine, McMaster University, Hamilton, ON, Canada; [7]Laboratoire de Chimie Bactérienne UMR7283, Centre National de la Recherche Scientifique, Aix-Marseille Université, Marseille, France

***Pseudomonas aeruginosa*** **(P.a) is a Gram-negative opportunistic pathogen that poses a major global health threat, particularly in immunocompromised individuals, patients with cystic fibrosis, and those with burn injuries or ventilator-associated pneumonia. Despite intense efforts, no vaccine is available for human use. In this context, live attenuated vaccines represent a promising but underexplored approach, offering the potential to elicit robust, long-lasting, and multifaceted immune responses including inducing trained immunity. Here, we sub-cultured *ΔLasB*-PAO1 (a P.a strain we have previously shown to have reduced virulence) in artificial sputum medium, a culture medium mimicking cystic fibrosis (CF) sputum in which bacteria often show auxotrophy. We showed that such strain ("V" for vaccine) was auxotrophic, less virulent, and had characteristics of "CF-like strains." Crucially, "V" induced both local (IgA) and systemic humoral responses as well as memory Th17 immune responses, and could, when administered in the lung, but not intra-muscularly, protect mice against a lethal PAO1-WT infection. Overall, the present study demonstrates that our vaccine formulation, in addition to providing an advantageous auxotrophic phenotype adapted to the CF setting, was efficient, when given mucosally and was associated with a pathway involving Th17 responses and secretory IgA, a critical barrier that neutralizes pathogens before tissue invasion.**

## INTRODUCTION

*Pseudomonas aeruginosa* (P.a) is a Gram-negative opportunistic pathogen that poses a major global health threat, in immunocompromised individuals, patients with cystic fibrosis (CF), and those with burn injuries or ventilator-associated pneumonia, and it has been classified as a critical priority pathogen.[1] Despite therapeutic advances in CF,[2,3] the rise of multidrug-resistant (MDR) and extensively drug-resistant (XDR) strains requires more effective preventive strategies. Despite this, no vaccine is currently available for human use. Although various platforms—including subunit, conjugate, and outer membrane protein-based vaccines—have been explored, most candidates have failed to provide broad, durable protection in clinical trials.[4–7] In this context, live attenuated vaccines (LAVs) represent a promising underexplored approach, to elicit robust, long-lasting, and multifaceted immune responses including trained innate immunity.[4–8] These include strong humoral and cellular ($CD4^+$/$CD8^+$ T cell) immunity—which closely parallels natural exposure.[9] Additionally, certain live vaccines (e.g., oral polio, rotavirus) administered mucosally stimulate localized IgA and mucosal immunity, which can reduce transmission—a benefit rarely seen with inactivated or subunit formulations.[10] Their replicative nature enables dose sparing, reducing antigen requirements and facilitating cost-effective mass production and distribution.[11]

However, traditional LAVs carry inherent safety risks. In immunocompromised patients, even attenuated strains may revert to a virulent form or cause unintended systemic infection. Moreover, LAVs require careful dose control and rigorous safety testing to minimize the risk of persistent colonization or dissemination, which could be dangerous in hospital or CF settings. Despite these risks, LAVs

Received 19 December 2025; accepted 15 June 2026;
https://doi.org/10.1016/j.ymthe.2026.06.029.

[8]These authors contributed equally

**Correspondence:** Youssouf Sereme, Institut National de la Santé et de la Recherche Médicale, U1149 Centre de Recherche sur l'Inflammation, Université Paris-Cité, 16 Rue Henri Huchard, 75018 Paris, France.
**E-mail:** youssouf.sereme@inserm.fr
**Correspondence:** Jean-Michel Sallenave, Institut National de la Santé et de la Recherche Médicale, U1149 Centre de Recherche sur l'Inflammation, Université Paris-Cité, 16 Rue Henri Huchard, 75018 Paris, France.
**E-mail:** jean-michel.sallenave@inserm.fr

 © 2026 The Authors.
Published by Elsevier Inc. on behalf of The American Society of Gene and Cell Therapy.
This is an open access article under the CC BY license (http://creativecommons.org/licenses/by/4.0/).

remain attractive because they stimulate both systemic and mucosal immunity, producing long-lasting protective responses.[4–7] It was shown that by engineering auxotrophic bacterial strains that lack aromatic amino acids (*ΔaroA*) or D-glutamate, such LAVs were replication-impaired outside of a controlled environment, while retaining immunogenic components and being incapable of causing disease.[12–16] Another interesting approach consisted of using an unnatural amino acid, creating a conditional *Pseudomonas* vaccine preventing bacterial infection.[17]

Here, we develop, for the first time to our knowledge, an auxotrophic P.a strain phenotypically as close as possible to clinical strains present in sputa secretions from patients with CF (pwCF), with reduced virulence while being robustly immunogenic. This was done by sub-culturing a genetically modified P.a (*ΔLasB*-PAO1) in artificial sputum medium (ASM), a culture medium mimicking CF sputum.[18–26]

We show by RNA-seq and whole genome sequencing (WGS) that it was indeed auxotrophic, less virulent, had characteristics of "small colony" CF-like strain, and secreted high amounts of filamentous phages (mainly Pf4). Crucially, it was able to induce both local (IgA) and systemic humoral responses as well as memory Th17 immune responses, and it could, when administered via the respiratory mucosal (but not intramuscular) route, protect mice against a lethal PAO1 infection.

## RESULTS

### Genetic profiling of PAO1 strains cultured in LB or ASM medium

#### *RNA-seq and whole genome sequencing of PAO1 strains cultured in LB or ASM medium*

To genetically characterize the P.a vaccine, we first analyzed by bulk RNA-seq 6 different conditions of culture for PAO1-WT or *ΔLasB*-PAO1, varying the time of culture or the medium used (PAO1-WT-LB-24hrs, PAO1-WT-ASM-24hrs, PAO1-WT-ASM-15days, *ΔLasB*-PAO1-LB-24hrs, *ΔLasB*-PAO1-ASM-24hrs, *ΔLasB*-PAO1-ASM-15days = "V"), ASM being a culture medium mimicking CF sputum,[20–26] secretions in which bacteria often show auxotrophy.[26–29] A heatmap (Figure 1A), with each column representing a cultured sample, highlighted the distinct profile of the "V" condition (green rectangle), which exhibited a global upregulation of numerous genes relative to the PAO1-WT-LB control (blue rectangle). Indeed, the UpSet plot representation showed that the contrast "V" versus PAO1-WT-LB (24hrs) had most differentially expressed genes (set size = 599), and among these, most ($n$ = 309) only belonged to that contrast (Figure 1B). Regardless, we showed that among the 20 most highly expressed genes across all samples (all 6 conditions/groups analyzed together) (Figure 1C) were *oprH*, *fliC*, *oprF*, and *oprI*, all genes already tested as subunit vaccines against P.a[4] (Figure 1C). In addition, notably, 5 genes were Pf4 bacteriophage genes (*PA0720*, *PA0723*, *PA0718*, *PA0724*, and *PA0726*)[30,31] and P.a. Pf prophages are known to play important roles in virulence and CF biofilm formation.[32,33] The 12.4-kb Pf4 genome comprises 20 genes (*PA0715–PA0729*; Figure S1A), 12 of which form the core region shared with the Pf6 prophage. Both Pf4 and Pf6 are co-produced in PAO1 biofilms, where they contribute to host antibiotic resistance and virulence.[34] Intrigued by this finding, we analyzed these 5 Pf4 genes, as well as other Pf4 genes in the 6 groups and showed that the transcription levels of the Pf4 genes belonging to the core region, including *PA0718*, *PA0720*, *PA0723/gVIII*, *PA0724*, and *PA0726* were dramatically increased in the "V" strain compared with the other groups (Figures 1D and S1B, Log2FC values between −7 and −11). By contrast, the expression of three genes (*PA015*, *PAO16*, *PAO16.1/pf4r*) were downregulated in the "V" strain (Log2FC values of values of +9, +8, and +8, respectively (Figure S1B red oval), compared to the PAO1-WT-LB (24hrs) strain and to the other groups (not shown). Interestingly, Guo et al.[34] recently reported that superinfective (SI) Pf4 prophages released during CF biofilm growth undergo an approximate 27% reduction in genome size, corresponding to the so-called 3.3-Kb discarded region (DR) containing the three genes *PA015*, *PAO16*, and *PAO16.1/pf4r* (Figure S1A). By comparison, most genes from the Pf6 prophage did not show global overexpression in the "V" strain, compared with the PAO1-WT-LB (24 h) strain (Figure S1C). Among some exceptions, however, three Pf6-specific genes—*pfkA*, *pfkB*, and *pfkC*—were overexpressed in the "V" strain, with $Log_2FC$ values of −3.5, −5, and −5, respectively (Figure S1C, red oval). Interestingly, as previously reported,[34] Pf6 *pfkA*, *pfkB*, and *pfkC* genes form a regulatory module that controls virion production of the co-resident Pf4 prophage. Altogether, this prophage transcriptomics analysis showing that Pf4 core genes (and the excisionase *xisf4*) were strongly upregulated and that genes *PA015*, *PAO16*, and *PAO16.1* (present in the DR region) were downregulated in the "V" strain suggested that culturing it during 15 days in ASM medium may have downregulated (or deleted) the genes present in the DR region of the Pf4 prophage DNA. To determine whether the DR genes were "simply" downregulated at the transcriptional level or whether that portion of phage DNA was indeed missing in the Pf4 genome, as evident in some P.a strains present in biofilms,[31,34] WGS was then performed in PAO1-WT-LB-24hrs (henceforth named "PAO1-WT") and in *ΔLasB*-PAO1-ASM-15d ("V" strain), 2 groups shown to be well differentially clustered (blue and green rectangles, Figure 1A).

Assembly and manual inspection of sequencing data obtained with Oxford nanopore technology led to a complete circular genomic sequence of 6,315,638 bp for the PAO1-WT strain (JBRXYX000000000) and 6,310,806 bp for the "V" strain (JBRXZF000000000).

Comparing the two genomic sequences displayed minimal differences, except obviously the absence of the *LasB* gene in the "V" strain and only a single nucleotide variant, located in an intergenic region. Nevertheless, a notable difference was observed in the Pf4 prophage sequence: the complete 12.4-kb Pf4 genome was detected only in the PAO1-WT strain, whereas the "V" carried only the 9.1-kb form, lacking the 3.3-kb region. Furthermore, we showed that this genotype was maintained over at least 57 passages. Indeed, we showed by qPCR that the Pf4r gene, present in the DR (Figure S1A), was

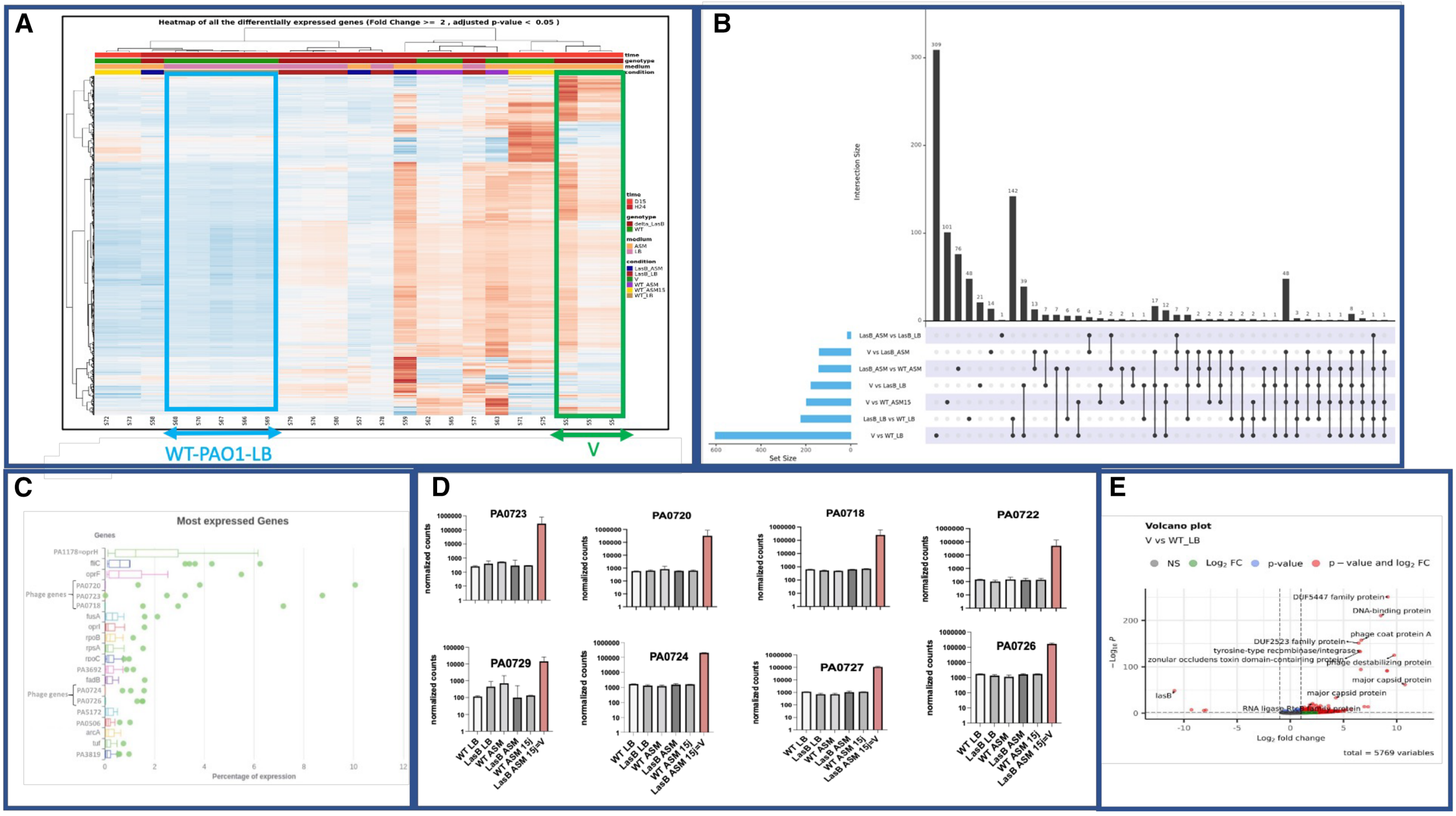


**Figure 1. RNA-seq analysis**

(A) Heatmap of differentially expressed genes (fold change > 2, adjusted $p < 0.05$). Heatmap representing all genes significantly differentially expressed between experimental conditions: PAO1-WT-LB-24hrs, PAO1-WT-ASM-24hrs, PAO1-WT-ASM-15days, *ΔLasB*-PAO1-LB-24hrs, *ΔLasB*-PAO1-ASM-24hrs, *ΔLasB*-PAO1-ASM-15days = "V": (fold change >2, adjusted $p$ value <0.05). Expression values are shown as $\log_2$-transformed, z score-normalized counts across samples. By applying row scaling, each row corresponds to a gene and each column to a sample. Colors indicate relative expression levels, with red representing higher and blue lower expression compared with the mean. Hierarchical clustering was applied to both genes and samples to highlight expression pattern similarities. (B) UpSet plot of intersecting differentially expressed genes across contrasts (see A). The plot displays the top 50 intersection sets, corresponding to genes exhibiting significant fold change variations (adjusted $p$ value <0.05, |fold change| > 2). Vertical bars represent the number of genes shared among specific combinations of contrasts, while horizontal bars indicate the total number of differentially expressed genes within each contrast. This visualization highlights the degree of overlap and specificity of transcriptional responses across experimental conditions. (C) Top 20 most highly expressed genes across all samples (see A). Displayed are the 20 genes with the highest expression levels across all RNA-seq samples, irrespective of experimental conditions. Expression is represented as a percentage of total transcriptomic reads per sample. (D) Top Pf4 bacteriophage normalized RNA counts are shown for the core region genes (see genomic diagram in Figure S1A). (E) Volcano plot showing differential gene expression between "V" and PAO1-WT-LB-24hrs. Volcano plot of all 5,769 genes showing $\log_2$ fold change versus $-\log_{10}$ adjusted $p$ value, with genes significantly upregulated in "V" relative to PAO1-WT-LB highlighted in red (adjusted $p \leq 0.05$, $|\log_2$ fold change$| \geq 1$).

virtually absent in the V strain, compared with the PAO1 strain (and that of several CF clinical strains, Figure S1D), in keeping with the fact that V consisted mainly of the secreted SI 9.1-kb form region. Conversely, the circularized form of the prophage (presence of the attP site), a marker of replication and secretion of Pf4, was present in much higher copies in V, compared with the other strains (Figure S1E).

Altogether, the WGS and qPCR analysis clearly confirmed the "prophage transcriptomics" data presented above, demonstrating that indeed, an SI 9.1-kb secreted form, lacking the 3.3-kb DR region of the full-size 12.4-kb Pf4,[31,34] was generated in the "V" strain. Furthermore, when comparing the "complete" PAO1 RNA-seq data (i.e., not restricted to prophage genes), from PAO1-WT and "V," it was striking that overall the top 12 mostly expressed genes in "V" (relative to PAO1-WT) were Pf4 genes and belonged to the Pf4 core region (Figure 1E; Table S1).

In addition to these phage genes, KEGG Pathway Enrichment analysis showed an induction of specific pathways in the "V" strain, i.e., "Biosynthesis of secondary metabolites," "Ribosomes," "Carbon metabolism," "Biosynthesis of amino acids," "Bacterial chemotaxis," "Flagellar assembly," "Other carbon fixation pathways, "Citrate cycle," "Biosynthesis of various nucleotide sugars," "Oxidative phosphorylation," "Phosphonate and phosphinate metabolism," "Xylene degradation," and "Fluorobenzoate degradation" (Figure S2). These pathways intersect at multiple levels, particularly in energy metabolism, redox reaction, cofactor biosynthesis, biosynthetic precursors, stress response, and virulence regulation in bacteria, closely mirroring the nutrient-restricted and redox-challenged environment

of CF. Indeed, the upregulation of secondary metabolite biosynthesis suggests that "V" is shifting metabolic resources from virulence toward a persistence mode of community behavior and survival (slow growth, persistence). Relatedly, the upregulation of the node "Biosynthesis of nucleotide sugars" suggests the generation of the "building blocks" used by glycosyltransferases to assemble a wide variety of extracellular polysaccharides, including exopolysaccharides and alginate, involved in biofilm matrix generation and structural stability (e.g., UTP-glucose-phosphate uridyltransferase GalU, alpha-1,3-rhamnosyltransferase, glycosyltransferase family 4 proteins) (Table S1). This is confirmed by the upregulation of genes involved in alginate biosynthesis regulators (e.g., *AlgR*, *MucA*, *MucB*, *MucC*, *AlgP*, *AlgZ)*, stress response σ factors (*RpoE*, *RphoH*, *SigX*, *AlgU*), and stress response and DNA repair (e.g., *RecA*, *LexA*, *OspR*, *KatA*, *AhpC*, *DsbA*, *peroxiredoxin*, *glutaredoxin*; Table S1). Also, iron metabolism genes (siderophore-like or redox proteins) were upregulated in "V" (bacterioferritin, bacterioferritin-associated ferredoxin, Bacteriohemerythrin, azurin, ferric iron uptake transcriptional regulator, *IscR*, *ErpA*, succinate dehydrogenase, cytochrome *b*556 subunit, cytochrome *c* oxidase subunits *CcoM/CcoQ-FixQ*). Interestingly, potentially feeding into that "stressed" phenotype, the upregulated KEGG nodes phosphonate and phosphinate metabolism, xylene degradation, and fluorobenzoate degradation, together with central nodes like carbon metabolism, the citrate cycle, and biosynthesis of amino acids, suggest a bacterial metabolic adjustment toward nutrient flexibility and survival in a "stressful" environment.

Together, these transcriptional and pathway changes suggest that strain "V" undergoes a broad metabolic and stress-adaptive reprogramming consistent with a transition toward persistence and survival under cystic-fibrosis-like environmental pressures.[18–26,35]

#### Δ*LasB*-PAO1 "V" strain cultured in ASM medium is auxotrophic and less virulent, with "small colony variant" characteristics

To examine the biologic property of the P.a vaccine (Δ*LasB-PAO1* "V"), we cultured the "V" strain (generated in ASM medium during a 15-day culture, as explained above) in the minimal medium M9 and found that only adding specific amino acids was able to restore its growth to a level comparable to that of PAO1-WT (Figure 2A), indicating an auxotrophic phenotype. In addition to auxotrophy, the phenotypic shape of the "V" colonies and their mobility behavior (twitching, swimming, swarming) were also akin to that of the small colony variants (scv) typically found in CF secretions[36,37] (Figures 2B and S3) that we also found in some of our clinical strains (Figure S3C). Importantly, the persistence of that phenotypic change was checked on multiple occasions, over more than 50 passages, and an example of a 7-day time course of sub-passaging PAO1 WT and V is shown in (Figure S3A). This shows, remarkably, a difference in pigmentation/color (from "greenish" to "yellowish") between the PAO1-WT and "V" strain, which may be caused by several factors, including partial bacterial lysis caused by prophage induction (see above), changes in metabolism including that of phenazines/pyocynins, pyoverdins, and flavin-related enzymes (6,7-dimethyl-8-ribityllumazine synthase, NADPH-dependent FMN reductase, ferredoxin FdxA, ferredoxin-NADP+ reductase, NO-inducible flavohemoprotein, FAD-binding oxidoreductase, quinolones; see Table S1). Indeed, we showed that pyoverdine and pyocyanin, two virulence factors giving a typical blue/greenish color to P.a secretomes, were downregulated in "V" (Figure 2A).

Functionally, the potential decreased virulence of "V" was demonstrated by its diminished capacity to adhere to a variety of eukaryotic cell lines (alveolar macrophages, epithelial cells, and dendritic cells; Figure 2C), and by its lesser cytotoxic properties on these cells (Figure 2D). We also compared the differential persistence of "V" and of PAO1-WT in primary bronchial epithelial cells from a healthy individual and from a CF patient and showed, while PAO1 was increasing in the 0- to 4-h period, V was, in contrast, cleared faster (Figures 2E and 2F). Also, by measuring the expression of a variety of inflammatory mediators (panels G and H), we showed, expectedly, that it was generally higher in CF cells, compared with "healthy/cells" (lower dCTs meaning higher expression), irrespective of whether cells were unstimulated ("Control") or infected with PAO1-WT or with V. In addition, although both strains were equally "inflammatory" in CF cells (panel G), probably reflecting a saturation effect in the latter, there was a trend, although not statistically significant, for a reduction, in healthy cells, in the levels of certain cytokines in V- versus PAO1 WT-infected conditions (panel H).

Crucially, we also demonstrated *in vivo* (Figure S4A), using an infectious dose ($5.10^7$ cfu) 50× higher than that used for our immunization protocols ($10^6$ cfu, see below), that compared with PAO1-WT, V bacterial load was 2–3 logs lower in bronchoalveolar lavages (BALs), lungs, and spleens, 24 h post infection (panel B). Furthermore, in keeping with the latter, the cytokine secretion was reduced in BALs and spleen extracts of V-infected animals (panels C-D).

In addition, using higher doses of bacteria, we performed dose-response infections and showed that, in accordance with the above data, V was much less virulent *in vivo* (100% and 0% mortality for $5/6.5.10^8$ cfu of PAO1 WT and V, respectively; panels E-F).

Altogether, the above *in vitro* and *in vivo* observations suggested the "V" strain to bear similarities with those adapted to "CF-live environments," being auxotrophic and less virulent than the PAO1 strain, thus supporting its utilization for immunization.[18–26,35]

#### Respiratory mucosal, but not intramuscular, immunization with Δ*LasB-PAO1* "V" strain induces robust mucosal immune responses

We then assessed the immunogenicity of the "V" strain in C57/Bl6 mice following vaccination via either a lung mucosal oro-pharyngeal route (referred indiscriminately to as "intra-tracheal" [i.t.]) or an intramuscular (i.m.) route. Thus, the mice were vaccinated at D0, D7, 14, and were sacrificed at D21 (Figure 3A). We showed that at

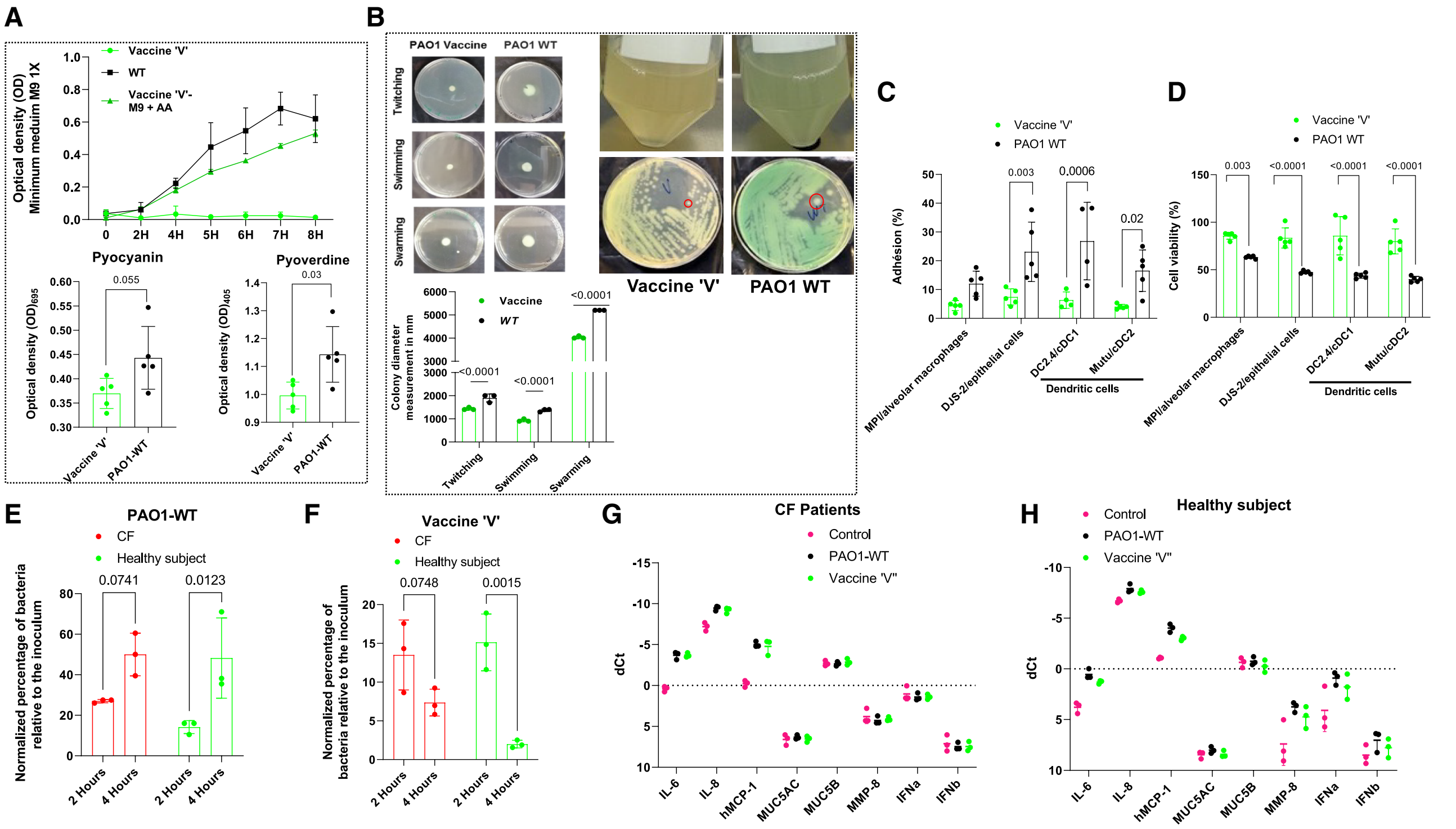


**Figure 2. Evaluation of the virulence of the PAO1 vaccine strain "V"**

(A) (1) Growth curves for P.a PAO1-WT-LB and the vaccine strain "V" in M9 minimal medium and M9 supplemented with amino acids (AA). (2) Pyocyanin and pyoverdin secretion in PAO1 WT and "V." (B) measurement of motility twitching, swimming, and swarming ($n = 3$) of the vaccine strain "V," compared with the PAO1-WT-LB strain ($p < 0.05$), and phenotypic appearance of colonies. Colony diameter measurement is presented as mean SD values. (C) Percentage of adhesion of PAO1-WT or "V" to alveolar macrophages (MPI cell line, $n = 5$), to lung club epithelial (DJS-2 cell line, $n = 5$), and to dendritic cells DC2.4/cDC1 ($n = 5$) and Mutu/cDC2 ($n = 5$). Two-way ANOVA with Tukey's multiple comparisons tests were used to determine statistical significance ($p < 0.05$). (D) Cell viability of MPI ($n = 5$), DJS-2 ($n = 5$), DC2.4 ($n = 5$), and Mutu/cDC2 ($n = 5$) was measured post infection with the bacterial strains (two-way ANOVA with Tukey's multiple comparisons, $p < 0.05$). (E and F) Primary bronchial epithelial cells from a healthy (H) or CF individual (CF) (Mucilair, Epithelix) were infected at a MOI of 1 with either PAO1-WT or V over 2 and 4 h. Bacterial load was then assessed by pooling supernatants and cell lysates. (G and H) An aliquot of cell lysates was used for RNA preparation, and cytokine/mediator levels were expressed as dCt/HPRT. NB: the lower the dCT, the higher the expression.

day 21 (1 week after the last boost), while both routes induced robust serum anti-PAO1 IgG production (Figures 3B and 3F), only i.t. immunization elicited high levels of specific anti-PAO1-IgG and -IgA in BALs (Figures 3D and 3E), when compared with i.m. vaccination (Figures 3G and 3H). In BALs, all the tested isotypes (IgG1, IgG2a, and IgG2b) were equally induced following i.t. immunization (Figure 3C).

Importantly, BAL IgG and IgA recognized both PAO1-WT and unrelated mucoid and non-mucoid clinical strains (respectively labeled 5–6; 8–20 and 7–23; 9–8; Figures 3D and 3E), and serum IgGs had the same cross-reactive properties (Figure 3F).

When cellular responses were assessed by flow cytometry at the same time point (Figure 4A, see Figures S5, S6, S7, and S8 for flow cytometry strategy) mucosal immunization with "V" was again more efficient at inducing immune responses in both lung tissues (Figure 4B) and BAL compartments (Figure 4D). Notably the numbers of neutrophils, dendritic cells (both cDC1 and cDC2), B cells, plasmocytes, and CD4 T cells (both classical and tissue-resident memory [TRM]) as well as those of CD8+ T cells were increased in lungs. Importantly, the proportion of IL-17+ cells among CD4+ T cells was increased in "V"-immunized animals after PMA/ionomycin stimulation (Figure 4C).

By comparison, none of the lung and BAL cell populations were increased in i.m-immunized animals (versus non-immunized animals) (Figures 4E and 4F).

After fully depleting CD4 T cells in blood, spleen, and lungs in PBS/V-immunized animals (Figure S9 panels B1-B2, E1-E2, F1-F2, H1-H2, I) and assessing for CD45 intra-vascular labeling (Figure S9 panels A2, B2, C2, D2, E2, F2, G2, H2), we showed that TRMs (initially present in the lungs of isotype-treated vaccinated mice, panel D1) were also efficiently removed (panel F1). Despite this, interestingly, intra-nasally (i.n) V-vaccinated CD4 T-depleted mice were equally protected against a lethal PAO1 challenge (panel J).

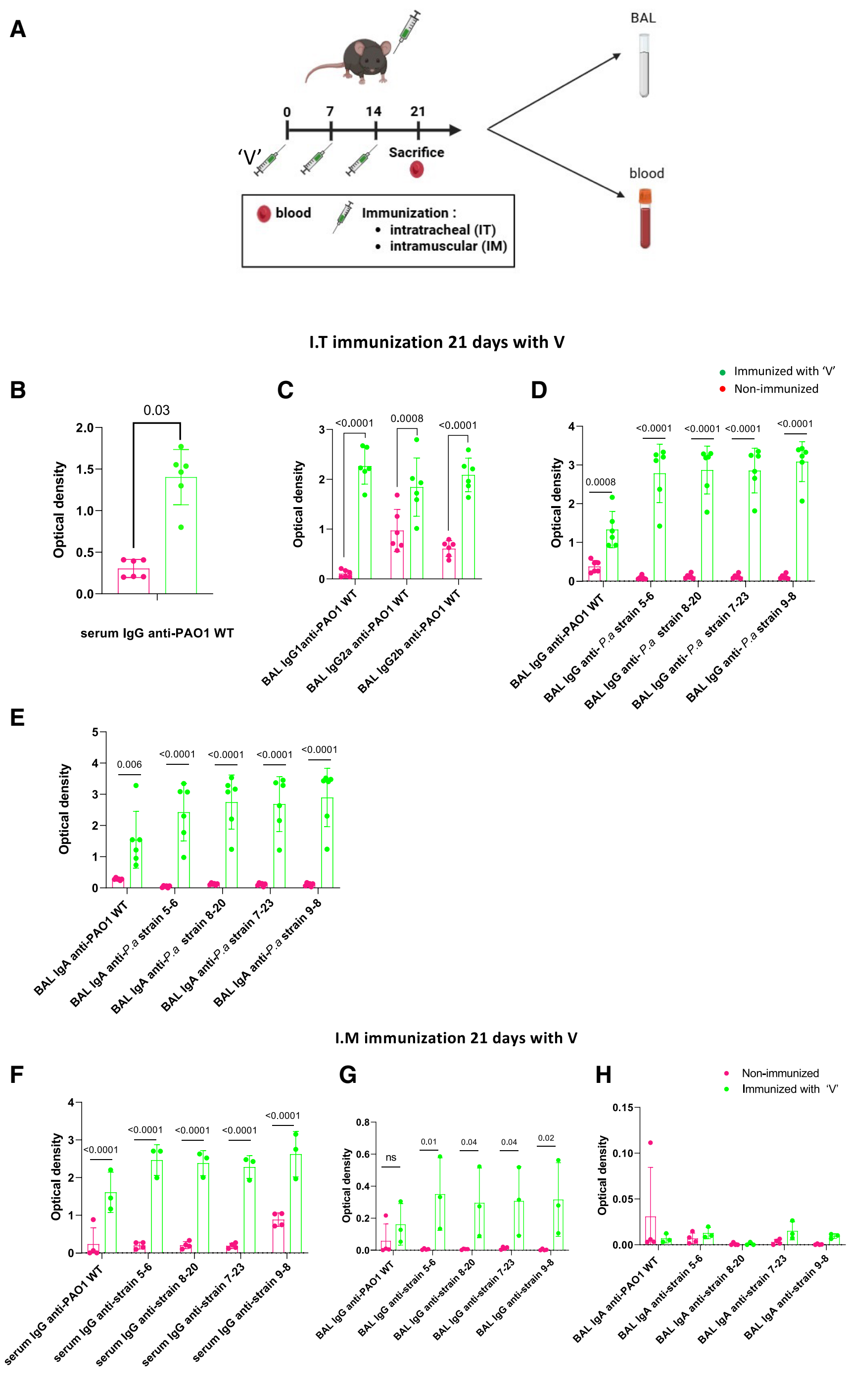


**Figure 3. Assessment of the immunogenicity of the "V" strain: Humoral response**

(A) Immunization protocol: at days 0, 7, and 14, C57BL/6 male mice were given (IT [$n = 6$] or IM [$n = 3$]) either PBS (control) or were immunized with $10^6$ CFU of the vaccine "V" strain. At day 21, 7 days after the last boost, BALs and blood were collected. (B and F) Measurement of anti-PAO1-WT, anti-P.a 5–6, 8–20 (clinical mucoid strains), and anti-P.a 7–23, 9–8 (clinical non-mucoid) serum IgG levels. (C) Measurement of anti-PAO1-WT IgG1, Ig2a, and IgG2b BAL levels. (D and G) Measurement of anti-PAO1-WT, anti-P.a 5–6, 8–20, 7–23, 9–8 BAL IgG levels. (E and H) Measurement of anti-PAO1-WT, anti-P.a 5–6, 8–20, 7–23, 9–8 BAL IgA levels. Statistics: Mann-Whitney non-parametric test for (A), and multiple *t* tests were used for (B–G), and error bars represent standard deviation (SD).

Then, by comparing the mucosal immunogenicity of the "V" strain to that of other PAO1 strains, i.e., PAO1-WT, *ΔLasB*-PAO1, and *ΔLasB*-PAO1 HI (heat-inactivated) (all strains cultured in LB), we showed that the specific anti-PAO1 antibody responses in serum and BALs were enhanced when the "V" strain was considered (Figure S10). When the cellular immune responses were assessed by flow cytometry, they were globally found to be equivalent, as assessed in BALs (Figure S11) and lungs (Figure S12), again demonstrating that the reduced virulence of the "V" strain (Figures 2 and S4) was not affecting its immune-stimulating potential (and was even beneficial in terms of antibody responses), compared with other PAO1 strains.

We therefore focused the rest of our study on the "V" strain and further showed by performing *ex vivo* tissue antigenic recall experiments (with killed PAO1-WT) that mucosal i.t. immunization was far superior at inducing a lung Th17 response (as measured by IL-17 levels) when compared with the i.m. vaccination protocol (Figures 5A and 5D). Interestingly, splenic responses were equivalent in terms of IL-17 recall responses, irrespective of the route of immunization (Figures 5C and 5F), and while spleen IFN-g levels were increased post i.m. immunization (Figure 5F), no splenic IFN-g was detectable post i.t. immunization (Figure 5C). When BAL cell antigenic recall was considered, IL-17 secretion was detectable in i.t.-immunized mice (Figure 5B) but not after i.m. vaccination (Figure 5E). IL-17 was likely produced by T cells, which accounted for roughly 20% of total cells in immunized mice (Figure 4D). On the other hand, BAL TNF (Figures 5B and 5E) was likely produced mainly by innate immune cells since these cells account for 95% of total cells in non-immunized mice (Figure 4D) and since the levels of this cytokine are roughly comparable in non-immunized and i.t. immunized mice (in the latter, resident AMs and dendritic cells account for to 40% and 20% of total cells, respectively, Figure 4D).

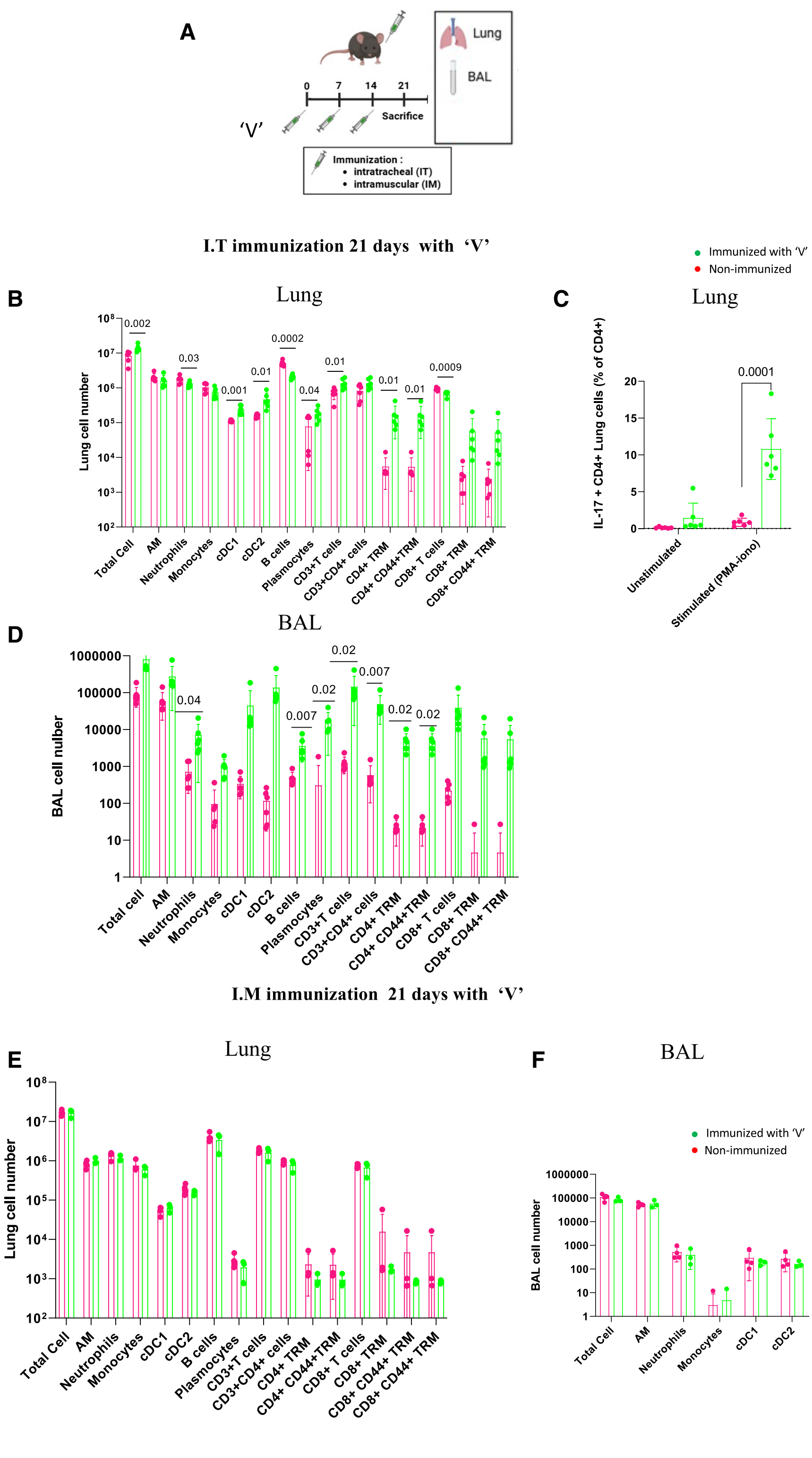


**Figure 4. Flow cytometry assessment of the immunogenicity of the "V" strain: Cellular response**
(A) Immunization protocol as in Figure 3. Counts of total cells, AMs, neutrophils, monocytes, dendritic cells (cDC1 and cDC2), B lymphocytes, plasma cells, CD3+ T cells, CD3+CD4+ T cells, CD4+CD44+TRM, CD8, CD8+TRM, and CD8+CD44+TRM T cells were assessed by flow cytometry in lungs (B and E) and BALs (D and F). (C) Cells were stimulated with PMA-ionomycin and percent of IL-17+CD4+ cells was assessed. NB: TRMs (tissue-resident memory cells) are defined here as CD3+CD69+CD103+. The flow cytometry strategies are depicted in Figures S5, S6, S7, and S8. Multiple *t* tests were used to determine significance, $p < 0.05$. Error bars represent standard deviation (SD).

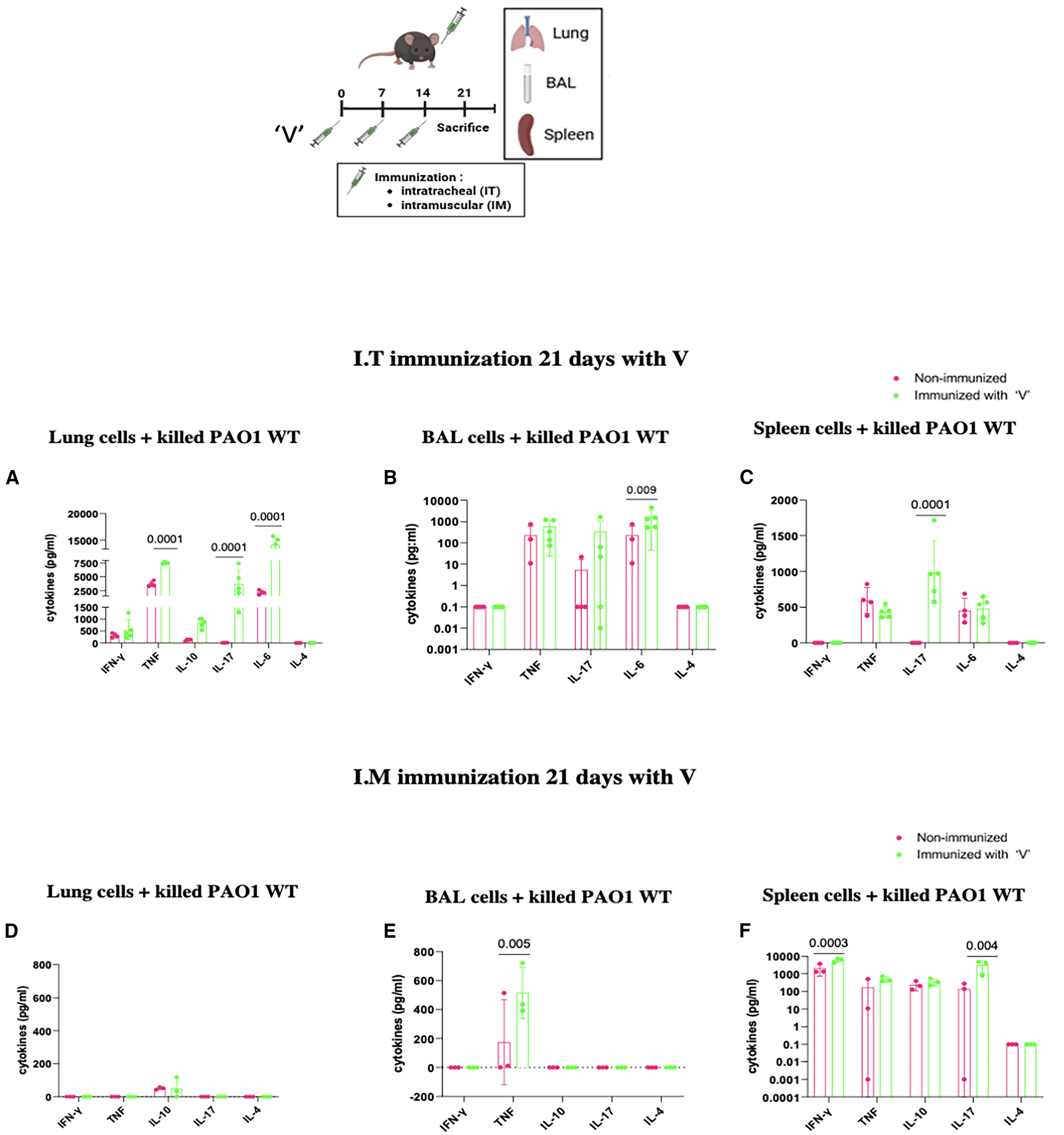


**Figure 5. Cytokine readout after *ex vivo* cell stimulation with killed PAO1-WT**

After i.t. or i.m. immunization (see Figures 3 and 4), lung (A and D), spleen (C and F), and BAL (B and E) were harvested, and cells were incubated ($10^6$, $10^6$, 20,000 cells, respectively) with gentamycin-killed bacteria (MOI = 1) during 48 h. IFN-g, TNF, IL-10, IL17, IL-6, and IL-4 cytokines levels were then measured (ELISA) in cell supernatants. Two-way ANOVA comparison tests were used for statistical analysis ($p < 0.05$). Error bars represent standard deviation (SD).

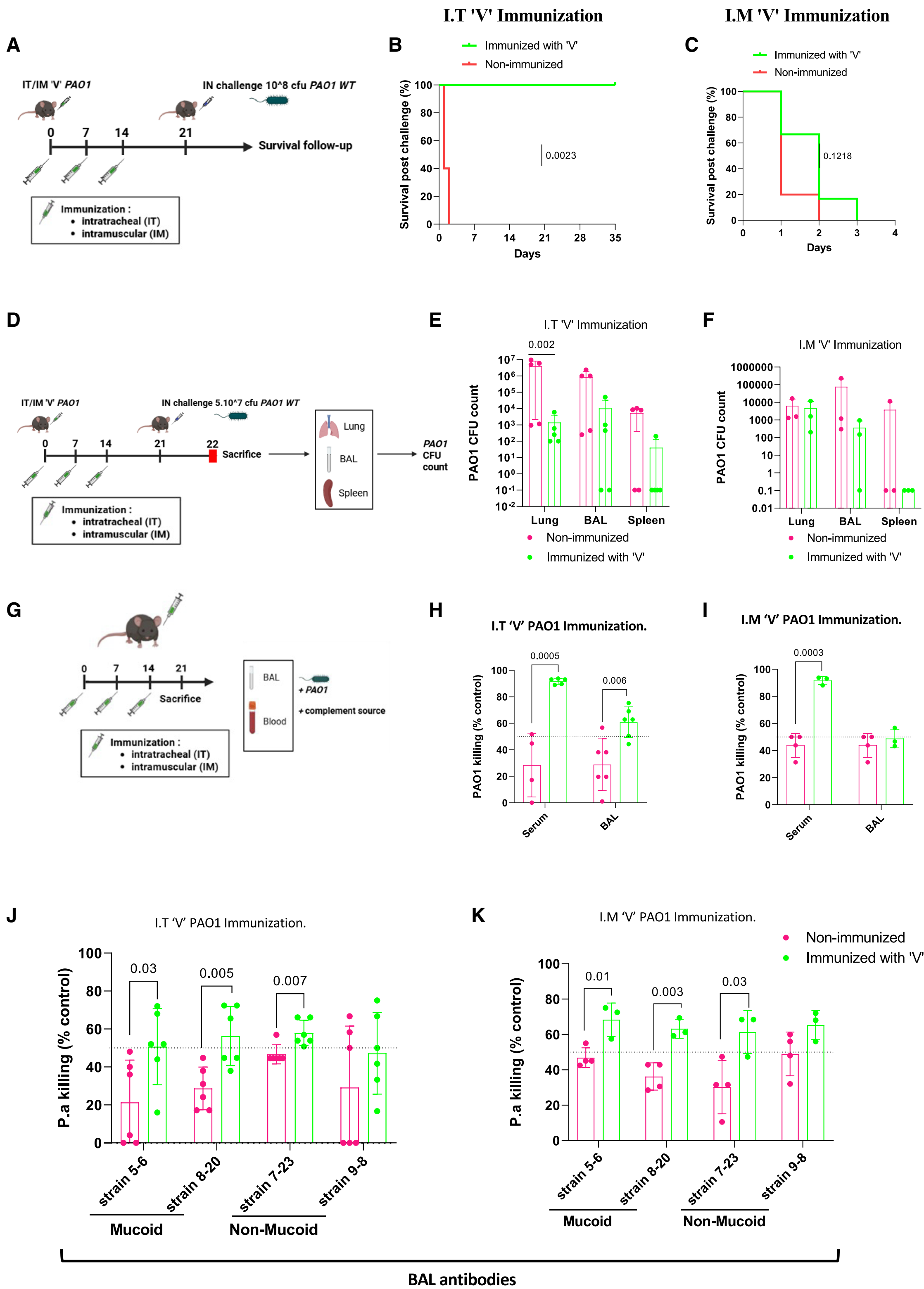


(legend on next page)

### Respiratory mucosal, but not intramuscular, immunization with Δ*LasB*-PAO1 "V" confers potent immune protection against a lethal PAO1-WT challenge

After demonstrating above the marked differences (and few similarities) between the i.t. and i.m. vaccination protocols in terms of immunogenicity, we then tested how this might affect protection against a lethal PAO1-WT ($10^8$ cfu) challenge, performed i.n. 7 days after the last boost (Figure 6A). In addition, to emphasize the importance of mucosal immunization over that of intramuscular vaccination, we followed the same protocol using also an i.n immunization procedure (Figure S13A) and showed that both i.t. and i.n. immunization were clearly more efficient (Figures 6B and S13A) than i.m. at protecting mice (Figure 6C).

To assess the magnitude of bacterial infection, we infected different cohorts of mice using a sub-lethal challenge dose of $5.10^7$ cfu of PAO1-WT (Figure 6D) and quantified bacterial counts at 24 h post challenge. While the bacterial load was very significantly diminished in the lungs of i.t. immunized (Figure 6E), it remained very high in i.m.-vaccinated mice (Figure 6F). In order to determine whether antibodies may have played a role in the clearing of bacteria noted above, we then tested *ex vivo* the complement-dependent bactericidal activity of serum and BAL antibodies against PAO1-WT and against the same P.a mucoid (5–6, 8–20) and non-mucoid (7–23, 9–8) clinical strains mentioned above (Figure 3). In keeping with data showing that both i.t. and i.m. immunization protocols generated strong antibody levels (Figure 3), we showed that these antibodies (from serum and BALs for i.t.-immunized animals, Figure 6H) were able to activate the complement and kill all P.a strains (PAO1-WT) and clinical strains 5–6, 8–20, 7–23, and 9–8 (BAL antibodies, Figures 6J and 6K). However, notably, the level of antibodies present in the BAL of mice i.m. immunized was not sufficient to kill P.a (Figure 6I).

### Respiratory mucosal immunization with Δ*LasB-PAO1* "V" induces long-lasting humoral and cellular immune responses

To determine the "intermediate" and "long-term" longevity of "V"-induced memory immune responses, we first assessed lungs, BALs, and spleens of mice at days 55 and 69 (Figures 7A and S13B), respectively 34 days and 48 days after the original i.t. or i.n. "V" immunization protocol and the ensuing i.n. PAO1-WT challenge (which resulted in 100% survival; see Figures 6B and S13A).

Importantly, we found that the levels of specific anti-PAO1-WT IgG and IgA were maintained in these BALs and sera at these "intermediate" time points (Figures 7B and S13C–S13E) to levels at least equivalent as those observed at day 21 following "simple immunization" (Figures 3C and 3D). Also, the serum antibodies were able, in a complement-dependent fashion, to induce the killing of both PAO1-WT and "V" strains (Figure S13F).

When lung immune cells were investigated by flow cytometry, it is noticeable that although there was a slight reduction at days 55 and 69 (approximately 1 log and ½ log for days 55 [i.t., Figure 7C] and 2 log and ½ log for 69 days [i.n., Figure S13G]) in the levels of lung CD4 and CD8 TRMs, respectively, when compared with day 21 (Figure 4B), they were still very significantly increased in "V"-immunized mice compared with their non-immunized counterparts. In keeping with this, histological observation of lungs at day 55 showed peribronchial and perivascular mononuclear cell aggregates in immunized mice (Figure 7F).

In BALs, expectedly, inflammation had almost completely subsided at that time point since alveolar macrophages (AM) levels returned to normal values (between 95% and 98%; Figures 7D and S13H). However, although representing very minor populations in absolute numbers, monocytes, neutrophils, and DC levels were still increased in vaccinated and challenged animals at days 55 and 69 compared with untreated mice, particularly in i.t.-immunized mice (Figure 7D). In contrast, no differences in lymphocyte numbers were observed in the spleens between treated and control animals (Figures 7E and S13I).

Upon *ex vivo* antigenic recall with killed PAO1-WT (using the same protocol applied post immunization on day 21; see Figure 5), lung parenchymal cells still secreted at these late time points (day 55 for i.t. immunization; Figure 8A; and day 69 for i.n. immunization; Figure S13B) impressively high levels of IL-17 (Figures 8B and S13J), respectively. Notably, lung cells also showed enhanced IL-6 secretion compared with non-immunized animals. Interestingly, BAL cells (and most likely AMs since they were the overwhelmingly predominant cell type in BALs at day 55 (Figure 7D) seemed to display a memory-like phenotype and responded differently to killed PAO1-WT in terms of TNF (statistical significance) and IL-6 production (trend) (Figure 8C). By contrast, surprisingly, after antigenic recall, splenic cell IL-17 levels from i.n. immunized animals were much higher than their i.t.-immunized counterparts (Figure S13K vs. Figure 8D), whereas splenic IL-6 levels were both increased in i.t. and i.n. protocols.

Finally, we analyzed the responses to a sub-lethal PAO1 WT challenge, 105 days ("long-term protocol") after the last immunization

**Figure 6. PAO1-WT challenge following either i.t. or i.m. immunization with the "V" strain**

C57BL/6 mice (A) were immunized i.t. (B) or i.m. (C) as in Figures 3 and 4. At day 21, 7 days after the last boost, mice ($n$ = 6 in each group) were challenged i.n. with $10^8$ cfu of PAO1-WT. Mice were observed for survival (Mantel-Haenszel log rank tests). (D) C57BL/6 mice were immunized i.t. or i.m. and were challenged i.n. with a sub-lethal dose ($5 \times 10^7$ cfu of PAO1-WT). 24 h later, mice were sacrificed, and bacterial cfu counts were determined in lungs, BALs, and spleens (E and F). Two-way ANOVA comparisons were used to determine statistical significance ($p < 0.05$). Error bars represent standard deviation (SD). (G) Sera and BALs were collected from sacrificed animals (D) and were assessed for complement-dependent bactericidal assays: bacterial counts were determined after incubating PAO1-WT (H and I) or P.a clinical strains (J and K) with either sera or BALs with an exogenous source of complement (see materials and methods for details). Results are expressed as percent of numbers of PAO1-WT input load. Two-way ANOVA comparisons were used to determine statistical significance ($p < 0.05$). Error bars represent standard deviation (SD).

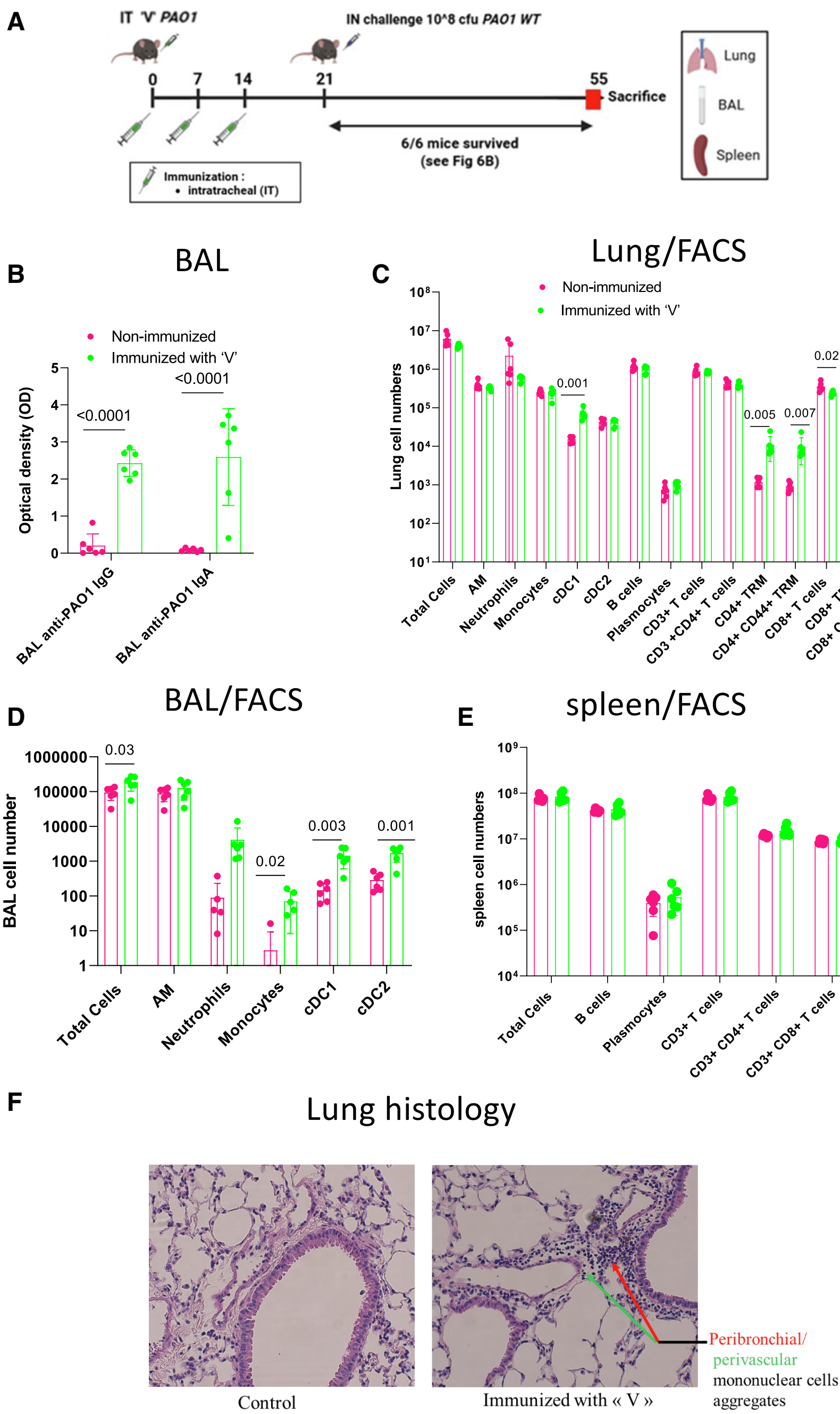


**Figure 7. Evaluation of "intermediate" memory immune responses at day 55, i.e., 41 days post "V" i.t. immunization and 34 days post challenge with PAO1-WT**

(A) Mice that survived the challenge with PAO1-WT (6/6, Figure 6) were observed until day 55 (i.e., 34 days post challenge). They were then sacrificed, and lungs, BALs, and spleens were collected. (B) Anti-PAO1-WT IgG and IgA were measured in BALs. (C–E) BAL, lungs, and spleen cellular immune responses were analyzed by flow cytometry as in Figure 5. Multiple *t* tests were used to determine statistical significance ($p < 0.05$). Error bars represent standard deviation (SD). (F) Lung histology was performed as described in materials and methods.

boost (Figure S14A) and showed that compared with non-immunized mice, long-term vaccinated mice, exhibited, 15 days post challenge, still impressive increased anti-PAO1 WT serum IgGs and BAL IgAs (Figures S14B and S14C).

When flow cytometry was performed on lung cells, we showed that CD4 T cells were still increased, and there was a trend toward increased levels of CD4 TRMs in "V"-immunized + PAO1-WT treated mice (Figure S14D). Similarly, in BAL cells, there was a trend toward increased neutrophils in the latter group, compared with the "non-immunized" + PAO1-WT treatment (panel E). In contrast, no differences in lymphocyte numbers were observed in the spleens between the 2 groups (panel F).

As above, upon *ex vivo* antigenic recall with killed PAO1-WT, the cytokine mostly upregulated was IL-17, both in lungs and the spleen, and yet again, the induction was more marked in cells from V-immunized animals (Figures S14G and S14H).

### Respiratory mucosal immunization with Δ*LasB*-PAO1 "V" induces high levels of anti-bacteriophage humoral responses

Given the very significant presence of the secreted SI form of Pf4 bacteriophages and the prevalence of the expression of their genes in the "V" strain (see above Figures 1 and S1; Table S1), we also specifically evaluated here anti-phage humoral responses. After showing that all protocols of immunization used throughout (with PAO1-WT, Δ*LasB*-PAO1, Δ*LasB*-PAO1-HI, "V") generated antibodies against PAO1-WT (Figure S10), we further demonstrated that all these protocols also induced, albeit at different levels, anti-phage antibodies in sera (IgG, Figure S15A) and in BALs (IgGs and IgAs) (Figures S15B and S15C). However, expectedly (since bacteriophages are strongly induced in the "V" strain), the levels of anti-phage antibodies were the highest when "V" was used as an immunogen, and as before, i.n./i.t. immunization was more effective than i.m. immunization.

In addition, to assess further the durability of the anti-phage antibodies, we analyzed the sera and BALs at day 120 as above

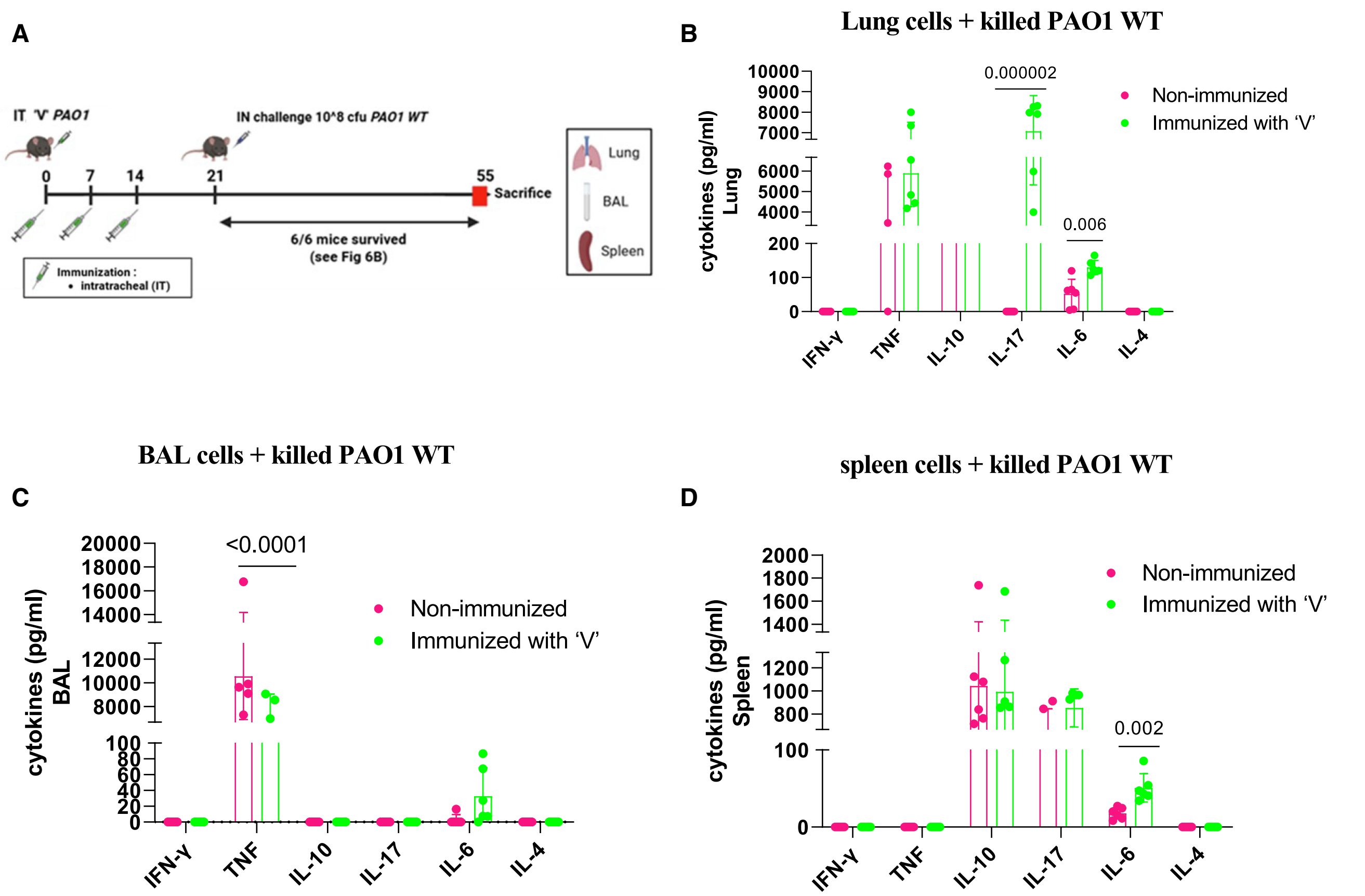


**Figure 8. Cytokine output following long-term immunization with "V" and challenge with killed PAO1-WT**

(A) Same as Figure 7. Lung (B), BAL (C), and spleen (D) cells (see Figure 7) were incubated with gentamycin-killed PAO1-WT (MOI = 1), as in Figure 5 legend. Cell supernatants were collected and IFN-g, TNF, IL-10, IL-17, IL-6, and IL-4 cytokine levels were measured. Two-way ANOVA comparisons were used to determine statistical significance ($p < 0.05$). Error bars represent standard deviation (SD).

(105 + 15). Indeed, we showed that "V"-immunized + PAO1-WT-treated mice had higher levels of serum anti-phage IgGs as well anti-phage IgGs and IgAs in BALs (Figures S15D and S15E).

## DISCUSSION

Despite decades of work, most historical *P. aeruginosa* vaccines failed to progress beyond encouraging murine data, partly because they predated the now well-recognized importance of respiratory mucosal route of immunization, IL-17-mediated mucosal immunity, and airway IgA.[4–7,9] Earlier vaccine efforts focused mainly on inducing systemic IgG through parenteral delivery, a strategy that offers limited protection at the airway surface where colonization and biofilm initiation occur. Moreover, few studies examined IL-17 responses or evaluated mucosal antibody induction.[13,38,39] Similarly, although some studies have explored auxotrophic P.a strain-based vaccines,[12,14,15,16,17] to the best of our knowledge, none have developed, critically, CF-adapted or "CF-like" vaccine strains, which differ markedly from acute laboratory isolates in virulence traits, biofilm behavior, and immune evasion. Although more recent preclinical work has begun to incorporate mucosal adjuvants, intranasal platforms, and Th17-skewing approaches, this historical lack of attention to mucosal immunity and to clinically relevant chronic strains likely contributed to earlier failures and underscores the need for strategies that generate local, IgA- and IL-17-competent immunity against the phenotypes dominating chronic lung disease. Indeed, respiratory delivery should allow for more precise targeting of both the upper and lower respiratory tract, ensuring consistent dosing and potentially enhancing immunogenicity at the primary site of pathogen entry. This is well illustrated—albeit outside the field of P.a vaccination—by efforts in viral immunization, such as FluMist/Fluenz Tetra for influenza and ChAdOx1 nCoV-19 for SARS-CoV-2 in phase I trials.[40–45] Relatedly, Jeyanathan et al. demonstrated in a recent phase I study that individuals who had received an mRNA COVID-19 vaccine (Pfizer and/or Moderna) showed no detectable lung mucosal immunity—neither humoral nor cellular—at least 3 months after their last dose. In contrast, inhaled aerosol delivery of a chimpanzee adenovirus platform (ChtriCoV/Mac) expressing three SARS-CoV-2 antigens effectively filled this gap in respiratory immunity, generating measurable CD8 T cell responses (both circulating and resident memory TRM) and protective antibodies up to 48 weeks post vaccination.[45]

In that context, we developed and characterized here a novel P.a vaccine strain ("V"), by maintaining a $\Delta LasB$-PAO1 strain in culture for 15 days in ASM (a culture medium mimicking CF sputum,[18–26] secretions in which bacteria often show auxotrophy.[26–29] As an attenuated *P. aeruginosa* strain, we selected this isolate based on our data showing that the LasB virulence factor is the most abundant protein in PAO1 secretomes,[46,47] and that the $\Delta LasB$-PAO1 strain—when cultured in standard LB medium—exhibited reduced virulence both *in vitro* and *in vivo*.[46,47] Furthermore, numerous studies have demonstrated that LasB is present in clinical samples either in early- or late-stage CF disease[48–50](for a recent review, see Everett and Davies[50]), and its presence has been associated with decreased antibiotic sensitivity and enhanced biofilm formation.[51,52]

A thorough WGS and RNA-seq analysis of our V strain (Figures 1, S1, and S2) demonstrated an increased expression of genes present in the core region of the filamentous prophage Pf4 and 3 genes of Pf6 (*pfkA*, *pfkB*, and *pfkC*), known to positively regulate Pf4. This induced the secretion of the SI form of the phage, known to be abundant in biofilms and to reduce, as demonstrated here, the twitching capacity of P.a.[31,32,53] In addition to the phage genes mentioned above, KEGG Pathway Enrichment analysis revealed upregulation of several metabolic pathways in the "V" strain (including "Biosynthesis of secondary metabolites, phosphonate and phosphinate, amino acid, and various nucleotide sugars"), all pointing toward activation of stress programs. Under these metabolically stressful conditions, the strain also exhibited an apparent auxotrophy for tryptophan, tyrosine, and phenylalanine. This functional auxotrophy, combined with the metabolic adaptations observed, reinforced the idea that the strain's physiological profile closely mirrored the nutrient-restricted and redox-challenged environment of the mucoid CF sputum.[18–26,35]

As a whole, this validated our hypothesis that the "V" strain may indeed be a highly relevant and promising vaccine candidate as a "CF-mimic" strain, including in patients with cystic fibrosis. Indeed, despite very significant advances in the field through the use of modulators and correctors,[2,3] significant side effects exist, and a sizable numbers of patients do not have access to these medicines, justifying the need for further approaches, including vaccinal ones.

Our data indicate that the engineered strain exhibited reduced *in vitro* and *in vivo* virulence (Figures 2 and S4) and was globally at least as immunogenic as other PAO1 strains (PAO1-WT, $\Delta LasB$-PAO1, $\Delta LasB$-PAO1-HI) cultured in classical LB medium, in terms of induction of antigen-specific cellular immune responses both in the airways and lung tissue (Figures S11 and S12), of antibody responses (notably IgAs) (Figures 3B–3E and S10), and in terms of lung IL-17 immunity (Figures 4B–4D, 5A–5C, S13J, and S13K).

Importantly, only respiratory immunization protocols significantly protected mice against a lethal challenge of PAO1-WT (Figures 6 and S13), and immune memory responses lasted for at least 120 days (Figure S14) in keeping with the increasingly recognized importance of mucosal immunization. Indeed, the antigen presentation and the associated immune responses including chemotactic and cell adhesion signals are expected to differ in totally different tissue environments following different routes of immunization. Ample preclinical[42,54–56] and clinical data[42] have shown that differently from i.t route, , the i.m. route of immunization results in immune responses sequestered within the peripheral blood and lymphoid tissues unless local lung immune signals are introduced.

Interestingly, we showed that the "V" strain, which greatly upregulated phage genes as discussed above (Figure 1) also induced increased anti-phage IgG and IgA antibodies up to 120 days (Figure S15), compared with the other PAO1 strains used.

This may be particularly relevant given the reported anti-phagocytic activity of these phages,[30] and consistent with this, Sweere et al. showed that anti-phage antibodies can significantly reduce wound infections caused by *P. aeruginosa*.[30]

Notably, the vaccine also conferred protective efficacy in mice lacking CD4 T cells during the effector phase of responses (Figure S9), suggesting that immunogenicity can be maintained even in a compromised immune setting. Mechanistically, although vaccine-induced protection was associated with increased IL-17 and IgA responses in CD4+ immunocompetent mice, this suggests the presence of redundant or compensatory protective mechanisms in CD4-mice, and that IL-17 may serve as correlate rather than direct mediator of immunity in this model. Also, even if directly implicated, IL-17 production may derive from $CD4^-$ cellular sources, including gamma delta T cells, natural killer T cells, and innate lymphoid cells, which could contribute to protection independently of conventional $CD4^+$ T cells.[57–62]

Beyond cystic-fibrosis-associated disease, significant unmet need also exists in non-CF respiratory infections, in COPD, bronchiectasis, or in hospital-acquired settings such as ventilator-associated pneumonia, where acute infection by opportunistic and often polymicrobial pathogens remains a major contributor to intensive care unit morbidity and mortality.[4–7,63,64]

In contrast to the chronic colonization characteristic of CF airways, these infections arise in the context of epithelial barrier disruption and critical illness, suggesting a complementary but distinct clinical niche for preventive and/or early therapeutic vaccination strategies. More broadly, similar considerations may apply to other high-risk populations with impaired barrier integrity, such as burn patients, in whom invasive bacterial infections continue to represent a major cause of adverse outcomes.

It should be noted that this study has several limitations, including the lack of in-depth mechanisms regarding the differential role of immune cells in the protective phenotype. In addition, even though it has been previously shown by others that experimental vaccines that were protective against PAO1 also offered enhanced protection against P.a strains of greater clinical relevance,[65–67] further studies

will be needed to confirm this with the V strain. Finally, to date, vaccine strategies against *P. aeruginosa* that have progressed into clinical evaluation have largely relied on killed whole-cell, polysaccharide, or protein-based formulations, including formaldehyde-inactivated vaccines, which demonstrated immunogenicity but limited clinical efficacy. In contrast, LAV approaches (e.g., auxotrophic or genetically attenuated strains) have shown promising protection in preclinical models but have not yet advanced into clinical trials.[4–7,9,11] Further preclinical and clinical evaluation of V will therefore be equally essential to balance the potential immunogenic benefits with the inherent risks of a live vaccine platform.

## MATERIALS AND METHODS

### Ethical statement

Animal experiments were carried out at the animal facility of the Paris-Cité Faculty of Medicine, Bichat site in Paris, in accordance with current regulations (Directive 2010/63/EU of September 22, 2010, and Decree no. 2013–118 of February 1, 2013, on the protection of animals used for scientific purposes). The protocol was approved by an animal experimentation ethics committee approved by the French Ministry of Higher Education and Research (APAFIS number: 27834).

Please see the supplemental information for the following: bacterial strains, culture, RNA-seq and whole genome sequencing; bacterial auxotrophy and motility tests; and cell line culture, maintenance, and bacterial infection.

### *In vivo* experiments

#### *Animals, anesthetizing procedures, and bacterial infection*

7- to 10-week-old male C57BL/6 mice were purchased from Janvier Labs. Animals were kept in a specific pathogen-free facility under 12-h light/dark cycles, with free access to food and water. Procedures were approved by our ethical committee and by the French Ministry of Education and Research (APAFIS number 27834). Prior to *in vivo* procedures, mice were anesthetized by intraperitoneal injection of 150 μL of a solution containing ketamine (10 mg/mL) and 2% Rompun (xylazine, 2 mg/mL) diluted in 0.9% NaCl. Bacteria were prepared from a frozen stock. After overnight culture, they were then diluted into fresh LB medium and incubated until reaching the exponential growth phase ($OD_{600}$ between 0.4 and 0.8). Bacterial concentration was estimated based on the relationship that an $OD_{600}$ of 1 corresponds to $10^9$ bacteria/mL. Immediately after administration, the exact number of administered bacteria were checked post hoc by diluting the vaccine dose (20 μL) on LB agar. Plates were incubated at 37°C overnight and colonies counted in order to determine that number.

#### In vivo *assessment of PAO1-WT and "V" differential clearance and virulence*

C57/Bl6 mice were infected i.n. with $5 \times 10^7$ cfu of either PAO1-WT or V. 24 h later, animals were sacrificed, and lungs, BALs, and spleen were recovered for CFU assessment. Cytokine levels were also measured by ELISA in BALs or spleen.

In independent experiments, to compare the virulence of PAO1-WT and V, mice were similarly infected with various doses of bacteria, and body weights and survival were followed over time.

#### In vivo *immune responses analysis*

C57/Bl6 mice were either mock-immunized (PBS) or immunized with three doses of various PAO1-WT strains ($10^6$ cfu of vaccine "V" PAO1 strain, PAO1-WT, *ΔLasB*-PAO1, *ΔLasB*-PAO1-HI, a dose chosen based on our historical experience on "benign," sub-lethal, and lethal doses) or with PBS, i.m. or mucosally (i.n./i.t.). Immunizations were performed via either the oropharyngeal/i.t. (the two terms are used indiscriminately throughout the manuscript) or i.n. at weekly intervals. This short interval was chosen to promote a rapid induction of the adaptive immune response, in line with previously described murine models using short intervals for the evaluation of highly immunogenic vaccines. This was followed by collection of blood, lungs, spleen, and BAL either at day 21, i.e., 7 days after the last boost, or at days 55 or 69, i.e., 34 or 48 days post PAO1-WT challenge ("intermediate memory experiments"). Alternatively, the responses to a sub-lethal PAO1 WT challenge, 105 days after the last immunization boost (3 immunizations, 1 week apart, as above) were analyzed 15 days after ("long-term memory experiments"). Organs and body fluids were then analyzed (see below and the supplemental information) with a variety of *ex vivo* readouts (flow cytometry, ELISAs, bacterial counts and killing assays, *ex vivo* antigenic recall, histology).

#### *Mouse survival after immunization and challenge*

After immunization with the "V" strain, either i.t., i.n. or i.m, (or mock-immunized with PBS) as explained above, different cohorts of mice were challenged at day 21 i.n. with a lethal dose of PAO1-WT ($10^8$ cfu) and were monitored for survival.

#### *Role of CD4 T cells in the protective phenotype*

Mice were mock vaccinated with PBS or with "V" as in Figures 6 and S13. At day 20, they were injected intraperitoneally with 250 μg of anti-CD4 antibody (or isotype) and i.n. with 30 μg of the same antibodies. At day 21, this was repeated i.n. with 30 ugs of antibodies. At day 23, 2 mice from each group were anesthetized and injected retro-orbitally with 2 ugs of anti-CD45-PB for intra-vascular labeling. 3 min later, these mice were sacrificed, and spleen and blood were retrieved, as well as lungs (after perfusion), for assessment of CD4 T cell depletion and CD45 labeling. The reminder mice in each group were challenged with a lethal dose of PAO1-WT ($10^8$ cfu), and survival was assessed.

#### *Measurement of anti-P.a-specific antibodies: Whole-cell ELISA*

Blood and bronchoalveolar lavage fluid samples were taken at day 21 after mock immunization or after days 21, 55, 69, and 120 (see materials and methods). PAO1-WT bacteria were inactivated at 90°C for 60 min after overnight culture, and centrifuged at $20{,}000 \times g$ for 10 min. Resuspended pellets were diluted to a $DO_{600}$ of 0.2, and 100 μL of the different bacterial suspensions was transferred to 96-well ELISA plates, which were incubated overnight

at 37°C. After blocking with 1% bovine serum albumin and washing with PBS containing Tween 20, 100 μL of mouse samples (serum and BAL, diluted 1/2) was added to each well and incubated for 2 h at 37°C. Next, after further washing, 100 μL of a 1:10,000 dilution of secondary anti-mouse antibodies (IgG [for serum and BAL], anti-H&L horse [for BAL], IgA anti-H&L goat, Abcam, Paris, France) coupled to horseradish peroxidase was added. After further washing as above, 100 μL of TMB substrate (Sigma T0440-1L, France) was then added for 30 min. In the absence of a standardized reference curve using purified antigen-specific antibodies, OD readings were performed at 450 nm using a TECAN reader.

### Data analysis

For most of our experiments, our group sizes ($N$ = 5–8) were calculated to provide >80% power and a significance level (α) of 0.05 to detect a 1-log difference in most of our readouts. Mice were randomized upon arrival from the vendor to ensure equal distribution of weights and age across cohorts. While investigators were not blinded during the vaccination phase, downstream experiments were performed by researchers blinded to the treatment groups to eliminate observer bias. Statistical analyses were performed using GraphPad Prism v10. Results are expressed as mean ± SD. Significance was determined using two-tailed Mann-Whitney tests, one-way ANOVA with multiple comparisons, or Mantel-Haenszel log rank tests, with $p < 0.05$ considered significant.

## DATA AND CODE AVAILABILITY

- All reagents will be available upon reasonable request, and all data are available in the main text or the supplemental information.
- The complete genomic and annotated sequences were deposited to NCBI GenBank: JBRXYX000000000 and JBRXZF000000000 (for PAO1-WT and "V" strains, respectively).

## ACKNOWLEDGMENTS

We wish to thank Mses Clémentine Cornu, Christelle Muñoz, and Blandine Arleri (JMS' laboratory) for their help with some of the experiments and Dr. Brice Barbat (LCB-UMR7283) for generating the *ΔLasB*-PAO1 mutant. We also thank Drs. Loredana Saveanu (CRI/INSERM U1149, Paris) and Imane El Meouche (INSERM, IAME, Paris) for useful discussions. We are also indebted to Prs. Catherine Llanes and Patrick Plésiat (Université Marie et Louis Pasteur, CNRS, Chrono-environnement [UMR 6249], Besançon, France) for their gift of the P.a clinical strains used here, to *Vaincre la Mucoviscidose* (grant RF20230503231), to a COPOC funding from Inserm (MAT-API-00990-A-02), and to the association *Gregory Lemarchal*. Thanks also go to Pr. E. Tartour (Hôpital Européen Georges-Pompidou, Paris) for reviewing a draft of this manuscript.

## AUTHOR CONTRIBUTIONS

Conceptualization: Y.S., J.-M.S.; investigation: Y.S., B.V., S.G., S.I., H.L., D.B., F.M., M.B.B., J.-M.S.; formal analysis: Y.S., B.V., S.C., F.M., J.-M.S.; resources: J.-M.S., R.V.; writing – original draft: Y.S., J.-M.S., S.C., F.M.; writing - review and editing: Y.S., B.V., S.C., S.I., F.M., C.D., M.B.B., Z.X., R.V., J.-M.S.; project administration: J.-M.S.; supervision: J.-M.S.; and funding acquisition: J.-M.S.

## DECLARATION OF INTERESTS

The authors declare that they have filed a patent application related to the technology described in this manuscript.

## SUPPLEMENTAL INFORMATION

Supplemental information can be found online at https://doi.org/10.1016/j.ymthe.2026.06.029.

## REFERENCES

1. Willyard, C. (2017). The drug-resistant bacteria that pose the greatest health threats. Nature *543*, 15. https://doi.org/10.1038/nature.2017.21550.
2. Flume, P.A., Biner, R.F., Downey, D.G., Brown, C., Jain, M., Fischer, R., De Boeck, K., Sawicki, G.S., Chang, P., Paz-Diaz, H., et al. (2021). Long-term safety and efficacy of tezacaftor-ivacaftor in individuals with cystic fibrosis aged 12 years or older who are homozygous or heterozygous for Phe508del CFTR (EXTEND): an open-label extension study. Lancet Respir. Med. *9*, 733–746. https://doi.org/10.1016/S2213-2600(20)30510-5.
3. Middleton, P.G., Mall, M.A., Dřevínek, P., Lands, L.C., McKone, E.F., Polineni, D., Ramsey, B.W., Taylor-Cousar, J.L., Tullis, E., Vermeulen, F., et al. (2019). Elexacaftor-Tezacaftor-Ivacaftor for Cystic Fibrosis with a Single Phe508del Allele. N. Engl. J. Med. *381*, 1809–1819. https://doi.org/10.1056/NEJMoa1908639.
4. Santamarina-Fernández, R., Fuentes-Valverde, V., Silva-Rodríguez, A., García, P., Moscoso, M., and Bou, G. (2025). Pseudomonas aeruginosa Vaccine Development: Lessons, Challenges, and Future Innovations. Int. J. Mol. Sci. *26*, 2012. https://doi.org/10.3390/ijms26052012.
5. Killough, M., Rodgers, A.M., and Ingram, R.J. (2022). Pseudomonas aeruginosa: Recent Advances in Vaccine Development. Vaccines (Basel) *10*, 1100. https://doi.org/10.3390/vaccines10071100.
6. Sharma, A., Krause, A., and Worgall, S. (2011). Recent developments for Pseudomonas vaccines. Hum. Vaccin. *7*, 999–1011. https://doi.org/10.4161/hv.7.10.16369.
7. Sainz-Mejías, M., Jurado-Martín, I., and McClean, S. (2020). Understanding Pseudomonas aeruginosa–Host Interactions: The Ongoing Quest for an Efficacious Vaccine. Cells *9*, 2617. https://doi.org/10.3390/cells9122617.
8. Benn, C.S., Amenyogbe, N., Björkman, A., Domínguez-Andrés, J., Fish, E.N., Flanagan, K.L., Klein, S.L., Kollmann, T.R., Kyvik, K.O., Netea, M.G., et al. (2023). Implications of Non-Specific Effects for Testing, Approving, and Regulating Vaccines. Drug Saf. *46*, 439–448. https://doi.org/10.1007/s40264-023-01295-3.
9. Minor, P.D. (2015). Live attenuated vaccines: Historical successes and current challenges. Virology *479–480*, 379–392. https://doi.org/10.1016/j.virol.2015.03.032.
10. Holmgren, J., and Czerkinsky, C. (2005). Mucosal immunity and vaccines. Nat. Med. *11*, S45–S53. https://doi.org/10.1038/nm1213.
11. Tang, Y.-D., Li, Y., Cai, X.-H., and Yin, X. (2025). Viral Live-Attenuated Vaccines (LAVs): Past and Future Directions. Adv. Sci. *12*, 2407241. https://doi.org/10.1002/advs.202407241.
12. Cabral, M.P., Correia, A., Vilanova, M., Gärtner, F., Moscoso, M., García, P., Vallejo, J.A., Pérez, A., Francisco-Tomé, M., Fuentes-Valverde, V., and Bou, G. (2020). A live auxotrophic vaccine confers mucosal immunity and protection against lethal pneumonia caused by Pseudomonas aeruginosa. Plos Pathog. *16*, e1008311. https://doi.org/10.1371/journal.ppat.1008311.
13. Wu, W., Huang, J., Duan, B., Traficante, D.C., Hong, H., Risech, M., Lory, S., and Priebe, G.P. (2012). Th17-stimulating protein vaccines confer protection against Pseudomonas aeruginosa pneumonia. Am. J. Respir. Crit. Care Med. *186*, 420–427. https://doi.org/10.1164/rccm.201202-0182OC.
14. Cabral, M.P., García, P., Beceiro, A., Rumbo, C., Pérez, A., Moscoso, M., and Bou, G. (2017). Design of live attenuated bacterial vaccines based on D-glutamate auxotrophy. Nat. Commun. *8*, 15480. https://doi.org/10.1038/ncomms15480.
15. Priebe, G.P., Meluleni, G.J., Coleman, F.T., Goldberg, J.B., and Pier, G.B. (2003). Protection against fatal Pseudomonas aeruginosa pneumonia in mice after nasal immunization with a live, attenuated aroA deletion mutant. Infect. Immun. *71*, 1453–1461. https://doi.org/10.1128/IAI.71.3.1453-1461.2003.
16. Priebe, G.P., Brinig, M.M., Hatano, K., Grout, M., Coleman, F.T., Pier, G.B., and Goldberg, J.B. (2002). Construction and characterization of a live, attenuated aroA deletion mutant of Pseudomonas aeruginosa as a candidate intranasal vaccine. Infect. Immun. *70*, 1507–1517. https://doi.org/10.1128/IAI.70.3.1507-1517.2002.
17. Pigula, M., Lai, Y.-C., Koh, M., Diercks, C.S., Rogers, T.F., Dik, D.A., and Schultz, P.G. (2024). An unnatural amino acid dependent, conditional Pseudomonas vaccine prevents bacterial infection. Nat. Commun. *15*, 6766. https://doi.org/10.1038/s41467-024-50843-7.

18. Kirchner, S., Fothergill, J.L., Wright, E.A., James, C.E., Mowat, E., and Winstanley, C. (2012). Use of artificial sputum medium to test antibiotic efficacy against Pseudomonas aeruginosa in conditions more relevant to the cystic fibrosis lung. J. Vis. Exp. e3857. https://doi.org/10.3791/3857.

19. Behrends, V., Geier, B., Williams, H.D., and Bundy, J.G. (2013). Direct assessment of metabolite utilization by Pseudomonas aeruginosa during growth on artificial sputum medium. Appl. Environ. Microbiol. *79*, 2467–2470. https://doi.org/10.1128/AEM.03609-12.

20. Sriramulu, D.D., Nimtz, M., and Romling, U. (2005). Proteome analysis reveals adaptation of Pseudomonas aeruginosa to the cystic fibrosis lung environment. Proteomics *5*, 3712–3721. https://doi.org/10.1002/pmic.200401227.

21. Duncan, R.P., Moustafa, D.A., Lewin, G.R., Diggle, F.L., Bomberger, J.M., Whiteley, M., and Goldberg, J.B. (2024). Improvement of a mouse infection model to capture Pseudomonas aeruginosa chronic physiology in cystic fibrosis. Proc. Natl. Acad. Sci. USA *121*, e2406234121. https://doi.org/10.1073/pnas.2406234121.

22. Maxwell, D.N., Kim, J., Pybus, C.A., White, L., Medford, R.J., Filkins, L.M., Monogue, M.L., Rae, M.M., Desai, D., Clark, A.E., et al. (2022). Clinically undetected polyclonal heteroresistance among Pseudomonas aeruginosa isolated from cystic fibrosis respiratory specimens. J. Antimicrob. Chemother. *77*, 3321–3330. https://doi.org/10.1093/jac/dkac320.

23. Aiyer, A., and Manos, J. (2022). The Use of Artificial Sputum Media to Enhance Investigation and Subsequent Treatment of Cystic Fibrosis Bacterial Infections. Microorganisms *10*, 1269. https://doi.org/10.3390/microorganisms10071269.

24. Neve, R.L., Carrillo, B.D., and Phelan, V.V. (2021). Impact of Artificial Sputum Medium Formulation on Pseudomonas aeruginosa Secondary Metabolite Production. J. Bacteriol. *203*, e0025021. https://doi.org/10.1128/jb.00250-21.

25. Turner, K.H., Wessel, A.K., Palmer, G.C., Murray, J.L., and Whiteley, M. (2015). Essential genome of Pseudomonas aeruginosa in cystic fibrosis sputum. Proc. Natl. Acad. Sci. USA *112*, 4110–4115. https://doi.org/10.1073/pnas.1419677112.

26. Winstanley, C., O'Brien, S., and Brockhurst, M.A. (2016). Pseudomonas aeruginosa Evolutionary Adaptation and Diversification in Cystic Fibrosis Chronic Lung Infections. Trends Microbiol. *24*, 327–337. https://doi.org/10.1016/j.tim.2016.01.008.

27. Barth, A.L., and Pitt, T.L. (1995). Auxotrophic variants of Pseudomonas aeruginosa are selected from prototrophic wild-type strains in respiratory infections in patients with cystic fibrosis. J. Clin. Microbiol. *33*, 37–40. https://doi.org/10.1128/jcm.33.1.37-40.1995.

28. Taylor, R.F., Hodson, M.E., and Pitt, T.L. (1992). Auxotrophy of Pseudomonas aeruginosa in cystic fibrosis. FEMS Microbiol. Lett. *71*, 243–246. https://doi.org/10.1016/0378-1097(92)90716-2.

29. Agarwal, G., Kapil, A., Kabra, S.K., Das, B.K., and Dwivedi, S.N. (2005). Characterization of Pseudomonas aeruginosa isolated from chronically infected children with cystic fibrosis in India. BMC Microbiol. *5*, 43. https://doi.org/10.1186/1471-2180-5-43.

30. Sweere, J.M., Van Belleghem, J.D., Ishak, H., Bach, M.S., Popescu, M., Sunkari, V., Kaber, G., Manasherob, R., Suh, G.A., Cao, X., et al. (2019). Bacteriophage trigger antiviral immunity and prevent clearance of bacterial infection. Science *363*, eaat9691. https://doi.org/10.1126/science.aat9691.

31. Secor, P.R., Burgener, E.B., Kinnersley, M., Jennings, L.K., Roman-Cruz, V., Popescu, M., Van Belleghem, J.D., Haddock, N., Copeland, C., Michaels, L.A., et al. (2020). Pf Bacteriophage and Their Impact on Pseudomonas Virulence, Mammalian Immunity, and Chronic Infections. Front. Immunol. *11*, 244. https://doi.org/10.3389/fimmu.2020.00244.

32. Rice, S.A., Tan, C.H., Mikkelsen, P.J., Kung, V., Woo, J., Tay, M., Hauser, A., McDougald, D., Webb, J.S., and Kjelleberg, S. (2009). The biofilm life cycle and virulence of Pseudomonas aeruginosa are dependent on a filamentous prophage. ISME J. *3*, 271–282. https://doi.org/10.1038/ismej.2008.109.

33. Whiteley, M., Bangera, M.G., Bumgarner, R.E., Parsek, M.R., Teitzel, G.M., Lory, S., and Greenberg, E.P. (2001). Gene expression in Pseudomonas aeruginosa biofilms. Nature *413*, 860–864. https://doi.org/10.1038/35101627.

34. Guo, Y., Tang, K., Sit, B., Gu, J., Chen, R., Shao, X., Lin, S., Huang, Z., Nie, Z., Lin, J., et al. (2024). Control of lysogeny and antiphage defense by a prophage-encoded kinase-phosphatase module. Nat. Commun. *15*, 7244. https://doi.org/10.1038/s41467-024-51617-x.

35. Marvig, R.L., Sommer, L.M., Molin, S., and Johansen, H.K. (2015). Convergent evolution and adaptation of Pseudomonas aeruginosa within patients with cystic fibrosis. Nat. Genet. *47*, 57–64. https://doi.org/10.1038/ng.3148.

36. Häußler, S., Ziegler, I., Löttel, A., Götz, F.V., Rohde, M., Wehmhöhner, D., Saravanamuthu, S., Tümmler, B., and Steinmetz, I. (2003). Highly adherent small-colony variants of Pseudomonas aeruginosa in cystic fibrosis lung infection. J. Med. Microbiol. *52*, 295–301. https://doi.org/10.1099/jmm.0.05069-0.

37. Häußler, S., Tümmler, B., Weißbrodt, H., Rohde, M., and Steinmetz, I. (1999). Small-Colony Variants of Pseudomonas aeruginosa in Cystic Fibrosis. Clin. Infect Dis. *29*, 621–625. https://doi.org/10.1086/598644.

38. Meynet, E., Laurin, D., Lenormand, J.L., Camara, B., Toussaint, B., and Le Gouëllec, A. (2018). Killed but metabolically active Pseudomonas aeruginosa -based vaccine induces protective humoral- and cell-mediated immunity against Pseudomonas aeruginosa pulmonary infections. Vaccine *36*, 1893–1900. https://doi.org/10.1016/j.vaccine.2018.02.040.

39. Priebe, G.P., Walsh, R.L., Cederroth, T.A., Kamei, A., Coutinho-Sledge, Y.S., Goldberg, J.B., and Pier, G.B. (2008). IL-17 is a critical component of vaccine-induced protection against lung infection by lipopolysaccharide-heterologous strains of Pseudomonas aeruginosa. J. Immunol. *181*, 4965–4975. https://doi.org/10.4049/jimmunol.181.7.4965.

40. Knisely, J.M., Buyon, L.E., Mandt, R., Farkas, R., Balasingam, S., Bok, K., Buchholz, U.J., D'Souza, M.P., Gordon, J.L., King, D.F.L., et al. (2023). Mucosal vaccines for SARS-CoV-2: scientific gaps and opportunities—workshop report. npj Vaccin. *8*, 53. https://doi.org/10.1038/s41541-023-00654-6.

41. Pilapitiya, D., Wheatley, A.K., and Tan, H.-X. (2023). Mucosal vaccines for SARS-CoV-2: triumph of hope over experience. eBioMedicine *92*, 104585. https://doi.org/10.1016/j.ebiom.2023.104585.

42. Jeyanathan, M., Fritz, D.K., Afkhami, S., Aguirre, E., Howie, K.J., Zganiacz, A., Dvorkin-Gheva, A., Thompson, M.R., Silver, R.F., Cusack, R.P., et al. (2022). Aerosol delivery, but not intramuscular injection, of adenovirus-vectored tuberculosis vaccine induces respiratory-mucosal immunity in humans. JCI Insight *7*, e155655. https://doi.org/10.1172/jci.insight.155655.

43. McMahan, K., Wegmann, F., Aid, M., Sciacca, M., Liu, J., Hachmann, N.P., Miller, J., Jacob-Dolan, C., Powers, O., Hope, D., et al. (2024). Mucosal boosting enhances vaccine protection against SARS-CoV-2 in macaques. Nature *626*, 385–391. https://doi.org/10.1038/s41586-023-06951-3.

44. Wang, S., Qin, M., Xu, L., Mu, T., Zhao, P., Sun, B., Wu, Y., Song, L., Wu, H., Wang, W., et al. (2023). Aerosol Inhalation of Chimpanzee Adenovirus Vectors (ChAd68) Expressing Ancestral or Omicron BA.1 Stabilized Pre–Fusion Spike Glycoproteins Protects Non–Human Primates against SARS-CoV-2 Infection. Vaccines *11*, 1427. https://doi.org/10.3390/vaccines11091427.

45. Jeyanathan, M., Afkhami, S., D'Agostino, M.R., Satia, I., Fritz, D.K., Miyasaki, K., Ang, J.C., Zganiacz, A., Howie, K.J., Swinton, M., et al. (2025). Induction of lung mucosal immunity by a next-generation inhaled aerosol COVID-19 vaccine: an open-label, multi-arm phase 1 clinical trial. Nat. Commun. *16*, 6000. https://doi.org/10.1038/s41467-025-60726-0.

46. Saint-Criq, V., Villeret, B., Bastaert, F., Kheir, S., Hatton, A., Cazes, A., Xing, Z., Sermet-Gaudelus, I., Garcia-Verdugo, I., Edelman, A., and Sallenave, J.M. (2018). Pseudomonas aeruginosa LasB protease impairs innate immunity in mice and humans by targeting a lung epithelial cystic fibrosis transmembrane regulator-IL-6-antimicrobial-repair pathway. Thorax *73*, 49–61. https://doi.org/10.1136/thoraxjnl-2017-210298.

47. Bastaert, F., Kheir, S., Saint-Criq, V., Villeret, B., Dang, P.M.-C., El-Benna, J., Sirard, J.-C., Voulhoux, R., and Sallenave, J.-M. (2018). Pseudomonas aeruginosa LasB Subverts Alveolar Macrophage Activity by Interfering With Bacterial Killing Through Downregulation of Innate Immune Defense, Reactive Oxygen Species Generation, and Complement Activation. Front. Immunol. *9*, 1675. https://doi.org/10.3389/fimmu.2018.01675.

48. Cigana, C., Castandet, J., Sprynski, N., Melessike, M., Beyria, L., Ranucci, S., Alcalá-Franco, B., Rossi, A., Bragonzi, A., Zalacain, M., and Everett, M. (2020). Pseudomonas aeruginosa Elastase Contributes to the Establishment of Chronic

Lung Colonization and Modulates the Immune Response in a Murine Model. Front. Microbiol. *11*, 620819. https://doi.org/10.3389/fmicb.2020.620819.

49. Wauters, M., Van den Bossche, S., Grassi, L., Fernandes Gonçalves, M., Van Haver, D., Dufour, S., Devos, S., Meuris, L., Impens, F., Saelens, X., and Crabbé, A. (2025). Unraveling the immunosuppressive role of Elastase B produced by cystic fibrosis isolates of Pseudomonas aeruginosa in an organotypic 3D lung epithelial cell model. Virulence *16*, 2581889. https://doi.org/10.1080/21505594.2025.2581889.

50. Everett, M.J., and Davies, D.T. (2021). Pseudomonas aeruginosa elastase (LasB) as a therapeutic target. Drug Discov. Today *26*, 2108–2123. https://doi.org/10.1016/j.drudis.2021.02.026.

51. Yu, H., He, X., Xie, W., Xiong, J., Sheng, H., Guo, S., Huang, C., Zhang, D., and Zhang, K. (2014). Elastase LasB of Pseudomonas aeruginosa promotes biofilm formation partly through rhamnolipid-mediated regulation. Can. J. Microbiol. *60*, 227–235. https://doi.org/10.1139/cjm-2013-0667.

52. Roshani-Asl, P., Rashidi, N., Shokoohizadeh, L., and Zarei, J. (2018). Relationship Among Antibiotic Resistance, Biofilm Formation and lasB Gene in Pseudomonas Aeruginosa Isolated from Burn Patients. Clin. Lab. *64*, 1477–1484. https://doi.org/10.7754/Clin.Lab.2018.180331.

53. Secor, P.R., Sweere, J.M., Michaels, L.A., Malkovskiy, A.V., Lazzareschi, D., Katznelson, E., Rajadas, J., Birnbaum, M.E., Arrigoni, A., Braun, K.R., et al. (2015). Filamentous Bacteriophage Promote Biofilm Assembly and Function. Cell Host Microbe *18*, 549–559. https://doi.org/10.1016/j.chom.2015.10.013.

54. Santosuosso, M., Zhang, X., McCormick, S., Wang, J., Hitt, M., and Xing, Z. (2005). Mechanisms of Mucosal and Parenteral Tuberculosis Vaccinations: Adenoviral-Based Mucosal Immunization Preferentially Elicits Sustained Accumulation of Immune Protective CD4 and CD8 T Cells within the Airway Lumen1. J. Immunol. *174*, 7986–7994. https://doi.org/10.4049/jimmunol.174.12.7986.

55. Santosuosso, M., McCormick, S., Roediger, E., Zhang, X., Zganiacz, A., Lichty, B.D., and Xing, Z. (2007). Mucosal luminal manipulation of T cell geography switches on protective efficacy by otherwise ineffective parenteral genetic immunization. J. Immunol. *178*, 2387–2395. https://doi.org/10.4049/jimmunol.178.4.2387.

56. Afkhami, S., D'Agostino, M.R., Zhang, A., Stacey, H.D., Marzok, A., Kang, A., Singh, R., Bavananthasivam, J., Ye, G., Luo, X., et al. (2022). Respiratory mucosal delivery of next-generation COVID-19 vaccine provides robust protection against both ancestral and variant strains of SARS-CoV-2. Cell *185*, 896–915.e19. https://doi.org/10.1016/j.cell.2022.02.005.

57. Bayes, H.K., Ritchie, N.D., and Evans, T.J. (2016). Interleukin-17 Is Required for Control of Chronic Lung Infection Caused by Pseudomonas aeruginosa. Infect. Immun. *84*, 3507–3516. https://doi.org/10.1128/IAI.00717-16.

58. Liu, J., Qu, H., Li, Q., Ye, L., Ma, G., and Wan, H. (2013). The responses of γδ T-cells against acute Pseudomonas aeruginosa pulmonary infection in mice via interleukin-17. Pathog. Dis. *68*, 44–51. https://doi.org/10.1111/2049-632X.12043.

59. Villeret, B., Ghinnagow, R., Kheir, S., Born-Bony, M., Kolls, J.K., Garcia-Verdugo, I., and Sallenave, J.-M. (2022). Pseudomonas Aeruginosa Lung Infection Subverts Lymphocytic Responses through IL-23 and IL-22 Post-Transcriptional Regulation. Int. J. Mol. Sci. *23*, 8427. https://doi.org/10.3390/ijms23158427.

60. Kumar, P., Chen, K., and Kolls, J.K. (2013). Th17 cell based vaccines in mucosal immunity. Curr. Opin. Immunol. *25*, 373–380. https://doi.org/10.1016/j.coi.2013.03.011.

61. Korn, T., Bettelli, E., Oukka, M., and Kuchroo, V.K. (2009). IL-17 and Th17 Cells. Annu. Rev. Immunol. *27*, 485–517. https://doi.org/10.1146/annurev.immunol.021908.132710.

62. Kheir, S., Villeret, B., Garcia-Verdugo, I., and Sallenave, J.-M. (2022). IL-6-elafin genetically modified macrophages as a lung immunotherapeutic strategy against Pseudomonas aeruginosa infections. Mol. Ther. *30*, 355–369. https://doi.org/10.1016/j.ymthe.2021.08.007.

63. Elmassry, M.M., Colmer-Hamood, J.A., Kopel, J., San Francisco, M.J., and Hamood, A.N. (2023). Anti-Pseudomonas aeruginosa Vaccines and Therapies: An Assessment of Clinical Trials. Microorganisms *11*, 916. https://doi.org/10.3390/microorganisms11040916.

64. Smith, D., Gill, A., Hall, L., and Turner, A.M. (2021). Prevalence, Pattern, Risks Factors and Consequences of Antibiotic Resistance in COPD: A Systematic Review. COPD *18*, 672–682. https://doi.org/10.1080/15412555.2021.2000957.

65. Wang, X., Liu, C., Rcheulishvili, N., Papukashvili, D., Xie, F., Zhao, J., Hu, X., Yu, K., Yang, N., Pan, X., et al. (2023). Strong immune responses and protection of PcrV and OprF-I mRNA vaccine candidates against Pseudomonas aeruginosa. npj Vaccin. *8*, 76. https://doi.org/10.1038/s41541-023-00672-4.

66. Kikuchi, T., Hackett, N.R., and Crystal, R.G. (2001). Cross-strain protection against clinical and laboratory strains of Pseudomonas aeruginosa mediated by dendritic cells genetically modified to express CD40 ligand and pulsed with specific strains of Pseudomonas aeruginosa. Hum. Gene Ther. *12*, 1251–1263. https://doi.org/10.1089/104303401750270913.

67. Peng, L., Wang, X., Sun, X., Cimino, J., Guan, Z., and Sun, W. (2021). Recombinant Pseudomonas Bionanoparticles Induce Protection against Pneumonic Pseudomonas aeruginosa Infection. Infect. Immun. *89*, e0039621. https://doi.org/10.1128/iai.00396-21.

**YMTHE, Volume *34***

## Supplemental Information

# A novel cystic fibrosis-mimetic *Pseudomonas* auxotrophic vaccine is protective *in vivo* and is associated with IL-17/IgA mucosal Immunity

**Youssouf Sereme, Bérengère Villeret, Ségolène Caboche, Samuel Ikeh, Hugo Leroy, Delphine Beury, Florence Maurier, Christophe Desterke, Maëlys Born-Bony, Zhou Xing, Romé Voulhoux, and Jean-Michel Sallenave**

# Supplemental information

## Supplemental Materials and Methods

### Bacterial strains and culture

PAO1-WT and clinical strains are described in Born-Bony M et al. (1) (**Table S3**). Briefly, PAO1 WT belongs to the serogroup 5 whereas the clinical strains 8-20 and 7-23 belong to the serogroups 2 and 16, respectively (the clinical strain 9-8 was non-typable).

Bacterial strains were initially recovered from frozen stocks (–80 °C) and streaked onto LB agar plates, followed by overnight incubation at 37 °C. A single isolated colony was then inoculated into liquid LB medium to prepare a preculture, which was incubated overnight at 37 °C under agitation (180–200 rpm). This preculture was used to inoculate a main bacterial culture, grown under the same conditions until reaching the exponential growth phase (OD600 ≈ 0.6–0.8).

*ΔLasB*- PAO1 was constructed from PAO1-WT strain(2) according to the protocol of Kaniga et al.(3) Briefly, to construct the pKNG101*ΔLasB* plasmid, two DNA fragments corresponding to the ~500-bp upstream and ~500-bp downstream regions of *LasB* gene (PA3724) were respectively amplified from PAO1 chromosomal DNA by PCR using primer couples A (5'CAGGTCGACGGATCCCCGGGTTGCCTTCAACATCGACCGC3') - B (5'ACCGAGCTTACTTCATCTTGTTCAGTTCTCC3') and C (5'AACAAGATGAAGTAAGCTCGGTGGTCCC3') – D 5'TATGCATCCGCGGGCCCGGGGGATTCGATGAAACGGGTGATG3') and ligated by overlapping PCR using primer couple A-D and AB & CD PCR products as DNA template.

The overlapping PCR product was cloned into linearized pKNG101 by the SLIC method. The resulting constructs were transformed into *E. coli* CC118λpir and introduced into P.a PAO1-WT by conjugation. The strains in which the chromosomal integration event occurred were selected on P.a isolation agar gentamycin (Gm) plates. Excision of the plasmid, resulting in the deletion of the chromosomal target gene was performed after selection on Luria-Bertani (LB) plates containing 6% sucrose. Clones that became sucrose resistant and Gm sensitive were confirmed to be deleted for the *LasB* gene by PCR analysis using primer couple E (5'GAGCCGATGTTCCACGGGG3' - F 5'ATCCAGCCGCCGTCGCCGA3'). The vaccine strain *ΔLasB*-PAO1 ('V' strain) was then obtained by sequential growth over 15 days in artificial sputum medium (ASM, complete composition given in[18]), by changing the ASM medium every four days. Before experiments, this 'V' strain and other P.a strains (PAO1-WT, *ΔlasB*-PAO1, *ΔlasB*-PAO1-HI (heat-inactivated), mucoïd and non mucoïd clinical strains, all cultured in LB medium) were grown overnight at 37 °C on LB agar plates. Single colonies were then inoculated into 10 mL of LB broth and incubated overnight at 37 °C with shaking (180 rpm) to prepare a pre-culture. The optical density (OD) at 600 nm was measured, and the bacteria were then diluted in fresh LB to reach an OD of 0.08. This culture was incubated at 37 °C with shaking for 2–3 hours until it reached exponential growth, corresponding to an OD between 0.4 and 0.8. The OD was measured again before further use.

To assess the impact of both genotype and culture conditions (24hrs or 15 days, under continuous selective pressure), six groups were defined at the onset of the study: PAO1-WT-LB-24hrs, PAO1-WT-ASM-24hrs, PAO1-WT-ASM 15days, *ΔLasB*-PAO1-LB-24hrs, *ΔLasB*-PAO1-ASM-24hrs, and *ΔLasB*-PAO1ASM-15days ('V' strain).

The phenotype of the V strain has been analysed through various experiments (see below) over 70 sequential passages.

**RNA-Seq and Whole genome sequencing analysis**

P.a PAO1-WT and *ΔLasB*-PAO1 strains were grown as indicated above, either in LB or in ASM, during 24hrs or 15 days. All conditions were performed in biological replicates, resulting in 23 RNA samples in total. Total RNA was extracted using the NucleoSpin RNA Kit (Macherey-Nagel, ref. 740955.50) according to the manufacturer's instructions. Ribosomal RNA was depleted using the QIAseq FastSelect -5S/16S/23S kit (Qiagen), and stranded libraries were prepared using the QIAseq Stranded RNA Library Kit (Qiagen). Sequencing was performed on an Illumina NovaSeq 6000 platform in paired-end mode (2 × 150 bp). Raw RNA-seq data have been deposited in the NCBI Sequence Read Archive (SRA) under BioProject accession number PRJNA1293891.

Reads were pseudo-aligned to a custom P.a transcriptome, based on GenBank: JBRXYX000000000 sequence. Transcript abundance was quantified using the nf-core/rnaseq v3.18.0 pipeline(4) and kallisto v0.51.1(5). Processed count data are available in the Gene Expression Omnibus (GEO) under accession number GSE303184. Differential expression analysis was performed using DESeq2 v1.38.3(6) and R v4.2.2 . Gene Set Enrichment Analysis (GSEA) was performed using clusterProfiler v4.6.2(7) and the Kyoto Encyclopedia of Genes and Genomes (KEGG) database to identify significantly enriched biological pathways. For q-PCR analysis, q-PCR of the Pf4 r gene (Pf4r-qF : AGTTTGGCCCGACTTCTGGG ; Pf4r-qR : CGTCAGCCCGATAGAGCAAGA) amplified a 206pb fragment in the the 3.3 kb DR (discarded region), absent in the excised SI Pf4 phage. Also, a q-PCR analysis of the circularization site of Pf4 (attP), located between the integrase gene and the repressor genes was performed (Pf4 FWD : TAGGCATTTTCAGGGGGCTTGG ; Pf4 REV : GAGCTACGGAGTAAGACGCC), amplifiying a 135 bp fragment. This allowed for the discrimination between the circularized Pf4 DNA and the linear Pf4 prophage sequence.

## Whole genome sequencing (WGS)

The bacterial strains were cultured in LB medium overnight at 37°C. PAO1-WT-LB-24hrs and *ΔLasB*-PAO1-ASM-15d 'V' strain genomic DNA was extracted with the QIAamp DNA mini kit (Qiagen). gDNA quality was evaluated using TapeStation 4200 (Agilent Technologies), and quantified by spectrophotometry (Nanodrop One, Thermo Fisher), and fluorimetry (Qubit, ThermoFisher). The libraries were prepared with the Native barcoding kit v14 (SQK-NBD 114.24, Oxford Nanopore Technologies), and following the Ligation Sequencing gDNA Native Barcoding for PromethION protocol (Oxford Nanopore Technologies). The sequencing was done with a PromethION flowcell on a PromethION 2 solo sequencer (Oxford Nanopore Technologies), with the super accuracy live basecalling mode, during 72 hours.

Adapters were trimmed from the raw reads using Porechop v0.2.4(8), and reads shorter than 500 bp were filtered out using NanoFilt v2.7.1(9). Sequences corresponding to the ONT internal positive control (DCS—DNA Control Strand) were removed by mapping reads to the DCS sequence with Minimap2 v2.28-r1209(10), using the -ax map-ont option, and retaining only unmapped reads. A total of 387,000 reads were retained for the PAO1-WT strain and 353,000 reads for the 'V' strain. Reads have been deposited in SRA under BioProject accession number PRJNA1353604. De novo assembly was performed using Flye v2.9.3-b1797(11) with the options --nano-hq --scaffold --asm-coverage 50 --genome-size 6.2m. Assembled sequences were compared using DNAdiff v1.3 from the MUMmer package(12). Manual curation was carried out by visualizing read mappings in the Integrative Genomics Viewer (IGV)(13).

Genome annotation was performed using NCBI prokaryotic genome annotation pipeline (PGAP)(14). Complete genomic and annotated sequences were deposited to NCBI GenBank under JBRXYX000000000 and JBRXZF000000000 IDs, respectively, for the PAO1-WT and 'V' strains.

**Bacterial auxotrophy test, pyocyanin and pyoverdine measurement.**

The various PAO1-WT strains were cultured until they reached the exponential growth phase (DO between 0.4 and 0.8), and the optical density (OD) was measured at 600 nm. The bacteria were then resuspended in 10 mL of laboratory-made M9 minimal medium (Gibco by Life Technologies) to obtain an OD of 0.05. Incubation was carried out at 37°C with agitation (210 rpm). DO was measured every two hours for bacteria grown in M9 medium and the *ΔlasB*-PAO1-ASM-15j-vaccine 'V' strain was also, when needed, grown in M9 medium supplemented with aromatic amino acids (40 µg/mL tryptophan, tyrosine and phenylalanine)(15). Pyoverdine and pyocyanin secretion was measured in PAO1 WT and V secretomes, at 405 nm and 695 nm, respectively, as in (16).

**Bacterial motility assays**

PAO1-WT strains were grown to exponential phase ($OD_{600}$ = 0.4–0.8). Swimming and swarming were assessed by spotting 5 µL of culture at $OD_{600}$ = 0.1 on 0.3 % agar plates. Plates were incubated at 37 °C for 8 h (swimming) or 24 h (swarming), and motility zones were measured. Contraction motility was evaluated by spotting 5 µL at $OD_{600}$ = 0.1 on 1 % agar overlying plastic. After 24 h, the agar was removed and bacterial spread on the plastic was measured.

**Cell line culture and maintenance**

DC2.4 dendritic cells (17) were cultured in IMDM supplemented with L-glutamine, HEPES, 10 % FBS, and penicillin/streptomycin. Cells were seeded at 2-3 × $10^4$ cells/mL in 100 mm dishes and passed when they are confluent after observation under a microscope. Adherent cells were detached using PBS with 10 mM EDTA. MUTU (DC1*,* MPI alveolar macrophages(18) and DJS-2 epithelial cells(19) were cultured similarly, using IMDM (MUTU), RPMI (MPI with 30 ng/mL mGM-CSF), or DMEM media (DJS-2). Adherent cells were recovered by EDTA or trypsin treatment, counted, and replated to 1 to 2.10^6 cells in a T150 flask in 30 ml of medium. Healthy and CF primary bronchial epithelial cells (Mucilair$^{TM}$) were obtained from Epithelix (Geneva, Switzerland), and cultured according to the manufacturer's instructions.

**Cell line culture and *in vitro* bacterial infection**

Cells (5 × $10^4$) from the indicated cell lines (see above) were seeded into 96-well plates in the appropriate culture medium (see above) and incubated overnight at 37 °C. Following bacterial culture, the different strains were resuspended in the corresponding cell culture media supplemented with 10% fetal bovine serum (FBS) without antibiotics, and infected with bacteria at a multiplicity of infection (MOI) of 1 (corresponding to 5 × $10^4$ bacteria per well).

After appropriate times (3-4 hours for MPIs, 4 hours for Epithelix cells and 24 hours for the other cell lines (DC2.4, MUTU, and DJS-2), and after centrifugation, cell culture supernatants were then removed and stored for subsequent cytokine quantification assays (ELISA RnD Diagnostics).

### *In vitro* PAO1 adhesion assay

Cells ($3 \times 10^5$ per well) were seeded in 16-well plates and incubated overnight at 37 °C in appropriate medium with 10 % FBS and antibiotics. PAO1 bacterial strains were prepared in media (see section above) with 10 % FBS and used to infect cells at a multiplicity of infection (MOI) = 1 during 1h. After washing thrice with PBS, cells were lysed with 500 µL PBS containing 0.02 % Triton on ice for 10 min. Released bacteria were plated on LB agar and incubated overnight at 37 °C for CFU enumeration.

### *In vitro* viability assay

Cells were plated in 96-well plates at $5 \times 10^4$ per well and incubated overnight. Cells (MPI, DC2.4, Mutus) were infected (MOI = 1) and incubated for 3 h (MPI) or 24 h (other cell lines, see above). Ten microliters of MTT labeling reagent (4,5-dimethylthiazol-2-yl)-2,5-diphenyltetrazolium bromide; Cell Proliferation Kit I (MTT), Sigma-Aldrich) was added to each well, followed by incubation for 4 hours at 37 °C. After incubation, the supernatant was removed and 50 µL of solubilization buffer (Cell Proliferation Kit I (MTT), Sigma-Aldrich) was added to each well. Plates were then incubated for an additional 30 minutes at 37 °C to allow complete solubilization of formazan crystals. Absorbance was measured at 540 nm using a TECAN microplate reader

## *Ex-vivo* tissue analysis

*a)Flow cytometry analysis*

BAL cells were pelleted, resuspended in DMEM with 10 % FBS and counted. After perfusion, lung tissue was digested with collagenase (1mg/mL) (Sigma C2139-500MG, France)/DNase (50 µg/ml) (Signa DN25-10 MG, France), filtered with 70 µm filters and then 40 µm filters (Gigma, France), centrifuged, treated with ACK lysis buffer, washed, and suspended (2.10^6 cells) in a final volume 100 µl PBS1X. Spleen cells were homogenized in a single-cell suspension through mechanical disruption. Briefly, the spleens were grinded, filtered on a 40µm sterile filter and 2.10^6 cells were resuspended in 100 µl PBS1X. All cells were first incubated (10 min, 4ºC) with a mixture of a viability dye and Fc Block antibody, washed (2,000 rpm, 5min) with FACS buffer (PBS, 1% BSA, 0.5Mm EDTA) and then incubated (30 min, 4ºC) with a cocktail of cell surface conjugated antibodies in FACS buffer with either a myeloid or lymphoid antibody panels (see **Table S2** for a list of antibodies). For intra-cellular IL-17 labelling, $10^6$ lung cells were stimulated with PMA/Ionomycin in the presence of monensin and brefeldin A for 4 h. Data were acquired the same day with a LSR Fortessa cytometer (BD Biosciences) with BD FACSDiva software and analysed with FlowJo (Tree Star, Ashland, OR, USA).

*b) Lung histology*

Perfused and fixed lungs (4%PFA in PBS, overnight at 4°C) were embedded in paraffin, sectioned in 4um slides and then stained with Hematoxylin-eosin by the INSERM U1149 'Plateforme de Morphologie'. Observations and microscopic photographs were taken at a 20X magnification.

*c) Ex-vivo antigenic recall with killed* PAO1-WT *and cytokine measurements*

Lung ($10^6$), spleen ($10^6$), and BAL ($2 \times 10^4$) cells were plated (96-well plate) and stimulated with gentamicin-killed PAO1-WT for 48 h in RPMI with 10 % FBS in a total volume of 0.2 ml. After centrifugation at 500g, supernatants were collected and analyzed for TNF- α, IFN-γ, IFN-α, IL-6, IL-4, and IL-17 by sandwich ELISA (Duoset Bio-Techne, France).

*d) Complement-dependent bactericidal activity assay*

Complement-dependent bactericidal activity was evaluated in serum and bronchoalveolar lavage (BAL) fluid from immunized and non-immunized mice against the PAO1 strain and four clinical isolates (5-6, 8-20 [mucoid], 7-23, and 9-8 [non-mucoid]), using a previously described method (20). Briefly, bacteria were grown overnight at 37°C in liquid LB medium and then diluted to an optical density at 600 nm (OD600) of 0.05. Cultures were incubated at 37°C until reaching the exponential growth phase (OD600 between 0.4 and 0.6). Bacteria were then washed twice and resuspended in PBS. An appropriate volume of the bacterial suspension was adjusted to obtain a final concentration of $10^4$ colony-forming units (CFU).

For the assay, 10 µL of bacterial suspension containing $10^4$ bacteria were mixed with 10 µL of heat-inactivated serum (65°C for 45 minutes), 10 µL of rabbit complement (3–4 weeks old; Pel-Freez® Biologicals, AR, USA, 15,20), and 10 µL of PBS in a 96-well plate, and the mixture was incubated at 37°C for 1 hour. After incubation, serial dilutions in PBS were performed, followed by plating on solid LB medium and overnight incubation at 37°C. Bacterial clearance was calculated by comparing the number of viable PAO1 and clinical strains at the beginning and end of the assay. Heat-inactivated sera from non-immunized mice were used as negative controls. Bactericidal activity greater than 50% in at least two dilutions was considered as a positive result.

**Purification and quantification of bacteriophages**

Bacteriophages were purified according to the protocol described by Sweere et al.[21] Briefly, the 'V' strain was cultured in 75 ml of LB broth for 48 hours at 37 °C with agitation. Bacteria were removed by centrifugation at 6,000 × g for 5 minutes, and the supernatant was treated with 1 µg/mL DNase I (Roche, ref. 4716728001) for 2 hours at 37°C before being sterilised by vacuum filtration through a 0.22 µm filter. Pf phages were precipitated from the supernatant by adding 0.5 M NaCl and 4% polyethylene glycol (PEG) 8000 (Sigma ref. P5413). All phage solutions were incubated overnight at 4°C. Then, they were centrifuged at 13,000 × g for 20 minutes, and the pellet was resuspended in sterile TE buffer (pH 8.0). The suspensions were again centrifuged at 15,000 × g for 20 minutes, and the supernatants were subjected to another cycle of PEG precipitation. The purified phage pellets were then resuspended in sterile PBS and concentrated on an Amicon UltraCell 100 (100 kDa molecular weight cut-off), and their DNA concentration checked by PCR

(not shown). The final solution was sterilized by filtration with a 0.2 µm filter (Millex™ polyethersulfone syringe filter, reference: 83.1826.001, Germany).

**Measurement of anti-phage specific antibodies (whole cell ELISA).**

Blood and bronchoalveolar lavage fluid samples were collected as indicated above (in the 'In *vivo experiments'* section e of the Main M&M). Purified phages were diluted in PBS and 100 µl of different dilutions were transferred to 96-well ELISA plates, which were incubated overnight at 37°C. After blocking with 1% bovine serum albumin (BSA) and washing with PBS, 100 µL of mouse samples (serum and BAL, diluted 1/2) were added to each well and incubated for 2 h at 37°C. Next, 100 µL of a 1:10000 dilution of secondary anti-mouse antibodies were added and readings performed as above (M&M, section E).

**Supplemental Table legends**

**Table S1 : Genes up-regulated in 'V' versus PAO1-WT (log2fold change >1 and p<0.05), see Excel file.**

**Table S2 :** List of antibodies used for flow cytometry experiments

| Antibodies | Fluorochrome | Isotype | Clone | Provider | Cat number | Dilution |
|---|---|---|---|---|---|---|
| CD45 | PE/Cyanine7 | Rat IgG2b, k | 30-F11 | Biolegend | 103113 | 1/200 |
| CD11b | PerCP/Cyanine5.5 | Rat IgG2b, κ | M1/70 | Biolegend | 101227 | 1/200 |
| CD11c | Pacific Blue | Armenian Hamster IgG | N418 | Biolegend | 117321 | 1/200 |
| Siglec F | BV711 | Rat LOU, also known as Louvain, LOU/C, LOU/M IgG2a, κ | E50-2440 | BD Biosciences | 740764 | 1/200 |
| I-A/I-E | APC | Rat IgG2b, κ | M5/114.15.2 | Biolegend | 107613 | 1/200 |
| Ly6G | Alexa Fluor 700 | Rat IgG2a, κ | 1A8 | Biolegend | 127621 | 1/200 |
| | | | | | | |
| Ly6C | PE | Rat IgM, κ | AL-21 | BD Biosciences | 560592 | 1/400 |
| Fixable Viability Dye | eFluor 506 | | | eBioscience | 65-0866-14 | 1/1000 |
| TruStain FcX ™ (anti-mouse CD16/32) | | Rat IgG2a, λ | 93 | Biolegend | 101320 | 1/200 |
| Mixed extra-myeloid cell marking | | | | | | |
| | | | | | | |
| CD45 | PE/Cyanine7 | Rat IgG2b, k | 30-F11 | Biolegend | 103113 | 1/200 |
| CD19 | Pacific Blue | Rat IgG2a, κ | 6D5 | Biolegend | 115526 | 1/200 |
| CD3ε | APC | Armenian Hamster IgG | 145-2C11 | Biolegend | 100311 | 1/100 |
| NK1.1 | Brilliant Violet 650 | Mouse IgG2a, κ | PK136 | Biolegend | 108735 | 1/100 |
| CD335 (NKp46) | FITC | Rat IgG2a, κ | 29A1.4 | Biolegend | 137606 | 1/200 |

| **Antibodies** | **Fluorochrome** | **Isotype** | **Clone** | **Provider** | **Cat number** | **Dilution** |
|---|---|---|---|---|---|---|
| CD4 | Alexa Fluor 700 | Rat / IgG2a, kappa | RM4-5 | eBiosciences | 56-0042-82 | 1/200 |
| CD8 | PerCP/Cyanine5.5 | Rat IgG2a, κ | 53-6.7 | Biolegend | 100733 | 1/200 |
| CD69 | PE-Dazzle | Armenian Hamster IgG | H1.2F3 | Biolegend | 104536 | 1/200 |
| | | | | | | |
| CD44 | PE | Rat IgG2b, κ | IM7 | Biolegend | 103024 | 1/200 |
| CD138 | Brilliant Violet 605 | Rat IgG2a, κ | 281-2 | Biolegend | 142515 | 1/200 |
| CD62L | APC/Cyanine7 | Rat IgG2a, κ | MEL-14 | Biolegend | 104428 | 1/200 |
| Fixable Viability Dye | eFluor 506 | | | eBioscience | 65-0866-14 | 1/1000 |
| TruStain FcX ™ (anti-mouse CD16/32) | | Rat IgG2a, λ | 93 | Biolegend | 101320 | 1/200 |

| Antibodies | Fluorochrome | Isotype | Clone | Provider | Cat number | Dilution |
|---|---|---|---|---|---|---|
| Mixed marking Mixed extra-cell lymphoid | | | | | | |
| | | | | | | |
| CD45 | Pacific Blue | Rat IgG2b, κ | S18009F | Biolegend | 157212 | 1/200 |
| CD11b | FITC | Rat IgG2b, κ | M1/70 | Biolegend | 101205 | 1/200 |
| IA-IE | FITC | Rat IgG2b, κ | M5/114.1 5.2 | Biolegend | 107605 | 1/200 |
| CD3ε | APC | Armenian Hamster IgG | 145-2C11 | Biolegend | 100311 | 1/100 |
| CD4 | Alexa Fluor 700 | Rat / IgG2a, kappa | RM4-5 | eBioscience s | 56-0042-82 | 1/200 |
| CD8 | PerCP/Cyanine5.5 | Rat IgG2a, κ | 53-6.7 | Biolegend | 100733 | 1/200 |
| CD25 | Brilliant Violet 650 | Rat IgG1, λ | PC61 | Biolegend | 102037 | 1/200 |
| Fixable Viability Dye | eFluor 506 | | | eBioscience | 65-0866-14 | 1/1000 |
| TruStain FcX ™ (anti-mouse CD16/32) | | Rat IgG2a, λ | 93 | Biolegend | 101320 | 1/200 |
| Mixed extracellular marking - Stimulation with PMA/Ionomycin | | | | | | |
| | | | | | | |
| FOXP3 | PE | Mouse IgG1, κ | 150D | Biolegend | 320008 | 1/40 |
| IL-17A | Brilliant Violet 711 | Rat IgG1, κ | TC11-18H10.1 | Biolegend | 506941 | 1/40 |
| RORgt | PE-CF594 | Mouse IgG2a, κ | Q31-378 | BD Biosciences | 562684 | 1/40 |

**Table S3 : Bacterial strains used in the study**

<table>
<tr><th>Strain</th><th>Type</th><th>Mucoid</th><th>Reference</th></tr>
<tr><td>PAO1-WT strain</td><td>Reference strain</td><td>No</td><td>(ATCC 15692)</td></tr>
<tr><td>ΔLasB-PAO1</td><td>Laboratory strain</td><td>No</td><td>(LCB-UMR7283, CNRS, Aix Marseille Université)[22]</td></tr>
<tr><td></td><td rowspan="7">Strains isolated from a cystic fibrosis (CF) patient</td><td></td><td rowspan="7">Ref [23]</td></tr>
<tr><td>5-6</td><td>Yes</td></tr>
<tr><td>8-20</td><td>Yes</td></tr>
<tr><td></td><td></td></tr>
<tr><td></td><td></td></tr>
<tr><td>7-23</td><td>No</td></tr>
<tr><td>9-8</td><td>No</td></tr>
</table>

**Supplemental Fig legends**

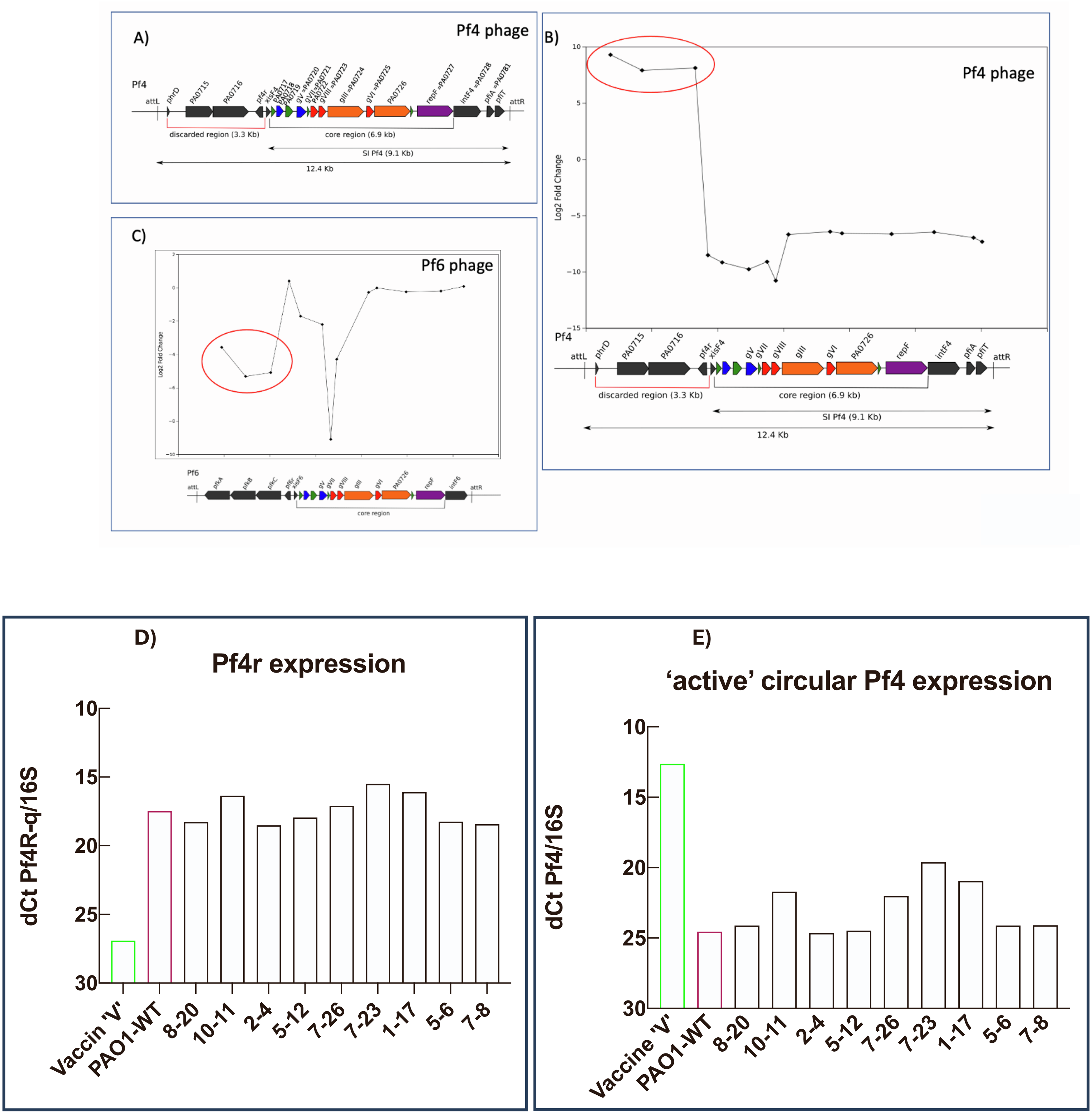


Fig S1

**Fig S1:**

A)Genomic organization of bacteriophage Pf4. B) RNA expression levels (RNAseq) of Pf4 genes expressed in Log2FC fold change ('V' = *ΔLasB*-PAO1-ASM-15d versus PAO1-WT-LB-24hrs). Genes circled (red) represent genes highly down-regulated in 'V', i.e *PA0715, PA0716, pf4r* from the discarded region (DR). C) Genomic organization of bacteriophage Pf6 and RNA expression (RNAseq) of Pf6 genes expressed in fold change as above ('V' = *ΔLasB*-PAO1-ASM-15d versus PAO1-WT-LB-24hrs). Genes circled in red represent genes up-regulated in 'V', i.e *pfkA, pfkB, pfkC,* genes forming a regulatory module controlling virion production of the co-resident Pf4 prophage[34]. **D)** The expression of the repressor r gene of Pf4 was analysed in PAO1 WT, 'V', and several CF clinical strains, by q-PCR amplifying a 206pb fragment in the R2 region of DR (discarded region). NB : the highest the dCT, the lower the level of expression (Pf4r-qF : AGTTTGGCCCGACTTCTGGG ; Pf4r-qR : CGTCAGCCCGATAGAGCAAGA). **E)** q-PCR analysis of the circularization site of Pf4 (attP), located between the integrase gene and the repressor genes was performed (Pf4 FWD : TAGGCATTTTCAGGGGGCTTGG ; Pf4 REV : GAGCTACGGAGTAAGACGCC), amplifiying a 135 bp fragment. This allowed for the detection of only the circularized Pf4 DNA and not the linear Pf4 prophage sequence. NB : the highest the dCT, the lower the level of expression.

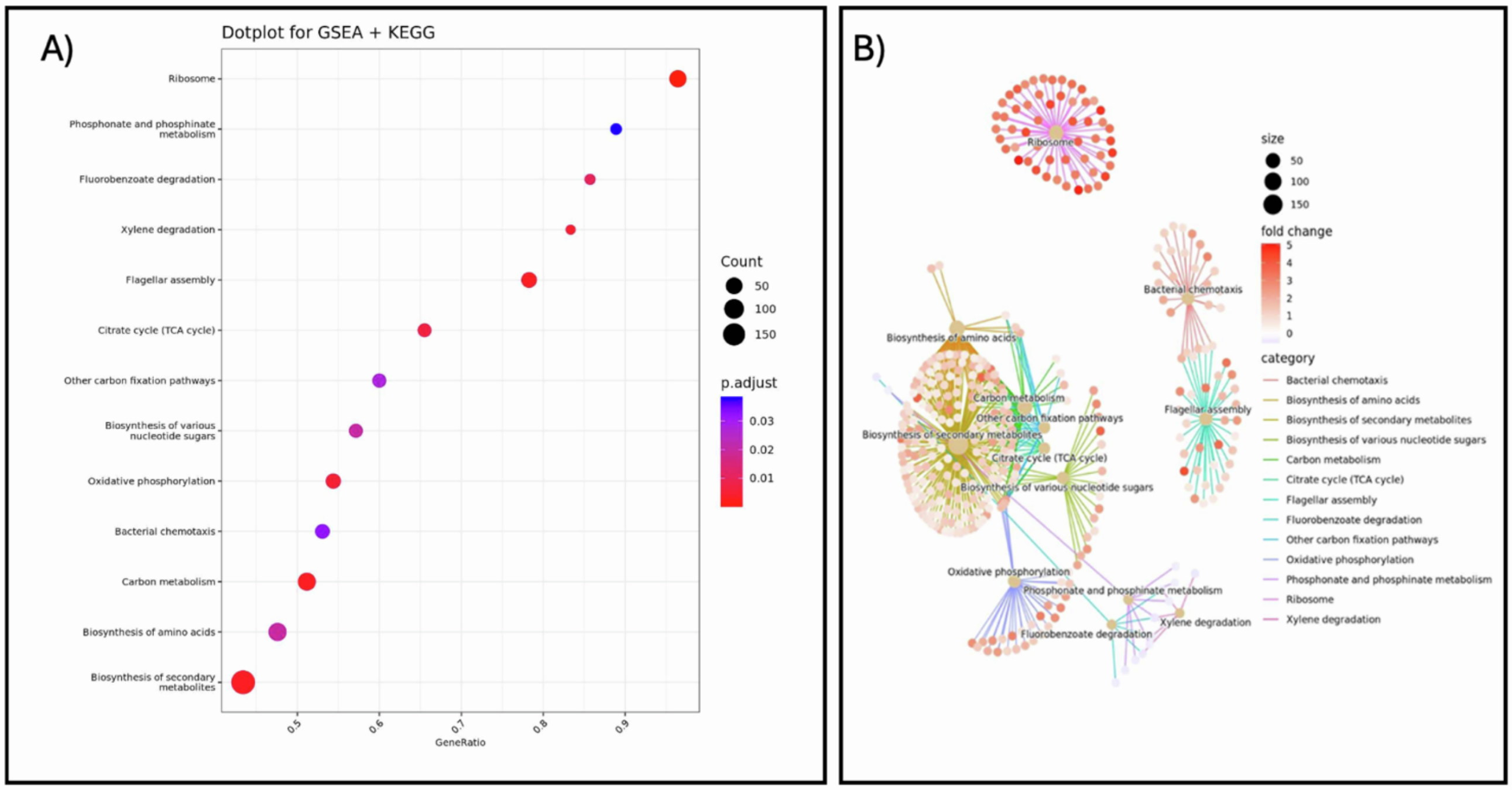


Fig S2

FigS2: A) KEGG pathway enrichment analysis of differentially expressed genes. Gene Set Enrichment Analysis (GSEA) was performed using the Kyoto Encyclopedia of Genes and Genomes (KEGG) database to identify significantly enriched biological pathways. The dot plot displays the 13 KEGG terms with the highest enrichment scores. Each dot represents a pathway, with the term names shown on the left of the plot. The gene ratio (proportion of input genes associated with each pathway) is indicated on the x-axis, ranging from 0.4 to 1.0**.** Dot size reflects the number of genes contributing to each pathway, and color intensity corresponds to the adjusted p-value (FDR)**,** with darker colors indicating higher statistical significance. B) : KEGG Gene-concept network

Shown are associations between differentially expressed genes (DEGs) and significantly enriched KEGG pathways identified in the RNA-seq dataset. Each large node represents an enriched KEGG term (e.g., *Ribosome*, *Flagellar assembly…*), with node size proportional to the number of DEGs mapped to that pathway. Small nodes correspond to individual DEGs, colour-coded according to

their $log_2$ fold change (red, upregulated; blue, downregulated). Edges connect genes to their associated KEGG pathways and indicate shared functional relationships.

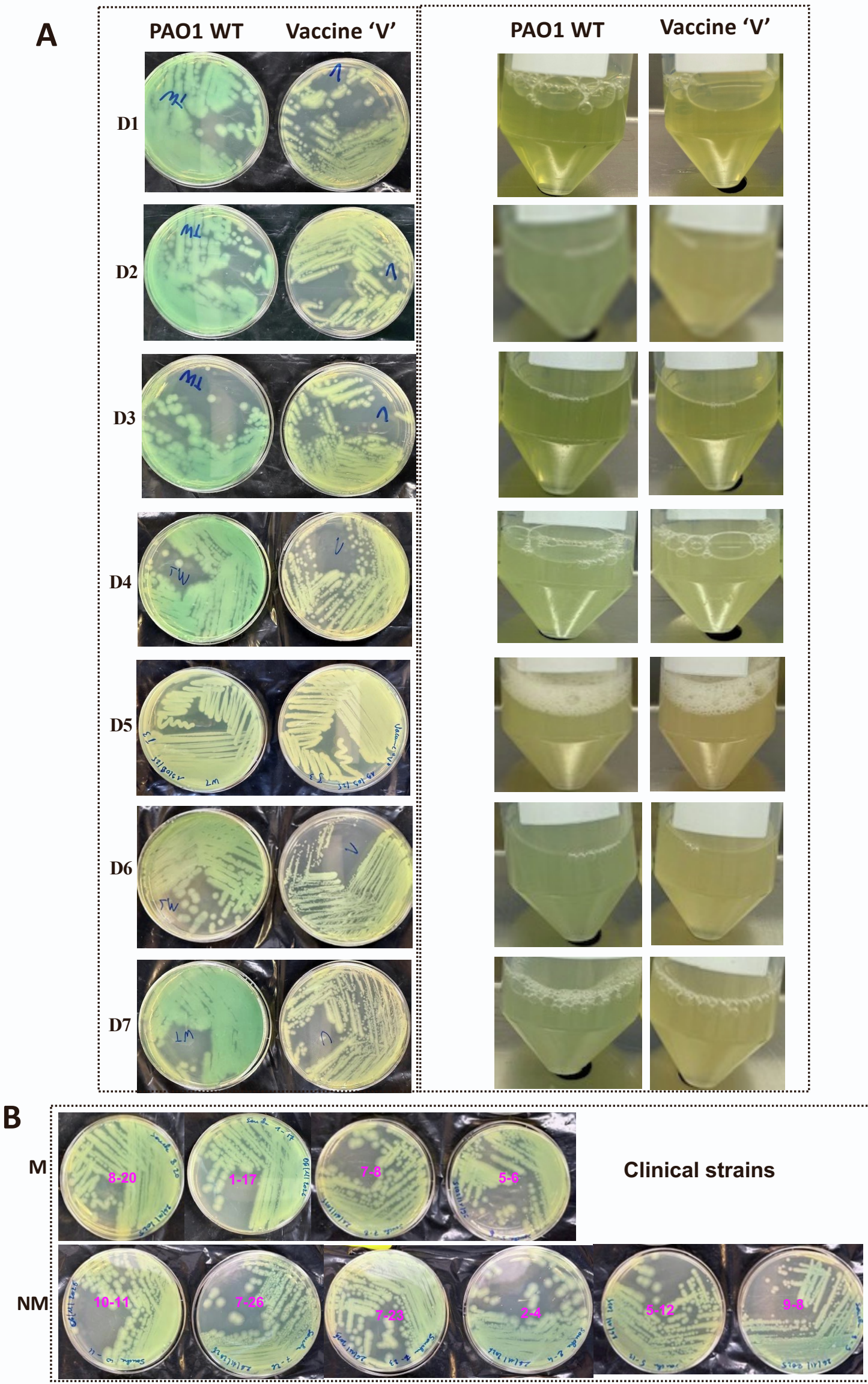


C

| Strains | PAO1 WT | Vaccine 'V' | 5-6 | 7-8 | 7-23 | 8-20 | 9-8 | 2-4 | 5-12 | 7-26 | 10-11 | 1-17 |
|---|---|---|---|---|---|---|---|---|---|---|---|---|
| Colony morphology | Large | Small | Large | Large | Large | Large | Small | Small | Small | Large | Small | Large |
| Auxotrophy | No | Yes | No | No | No | Yes | No | No | No | Yes | Yes | No |
| Mucoid | - | - | Yes | Yes | No | Yes | No | No | No | No | No | Yes |

Fig S3

**Fig S3** :
**(A)** Phenotypic characterization of WT and vaccine strains, colony appearance after successive subculturing for 7 days.
(**B**) Phenotypic characterization of 10 clinical strains (M=Mucoid, NM=Non-mucoid). (**C**) Microbiological characterization of strains (colony morphology, small/large ; auxotrophy ; mucoid status)

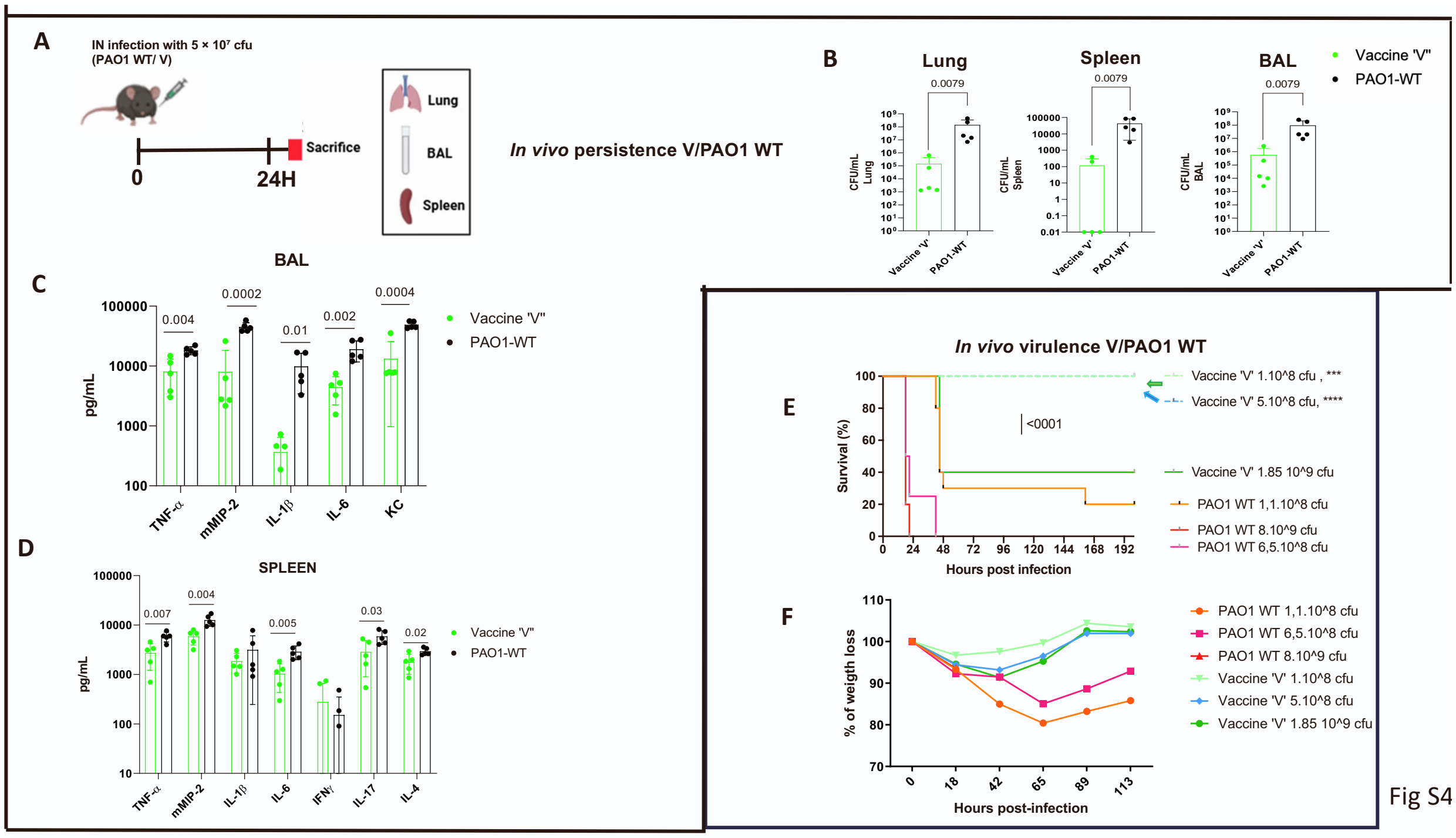


**Fig S4 *In vivo* persistence and virulence of PAO1-WT and V**

A-D) C57/Bl6 mice were infected i.n with 5 x 10^7 cfu of either PAO1-WT or V (A). 24 hrs later, animals were sacrificed, and lungs, BALs and spleen were recovered for CFU assessment (B). Cytokine levels were also measured by ELISA in BALs (C) or spleen (D)

E-F) In independent experiments, mice were similarly infected with various doses of PAO1-WT or V and survival (panel E) and weights (panel D) were followed over time. Survival (Kaplan-Meier representation was plotted and Log-Rank (Mantel-Cox) tests performed : *** (p=0.0005) : V 1.10^8 pfu/PAO1 WT ;  **** (p<0. 0001) : V 5.10^8 pfu/PAO1 WT. NB : in E), the survival of the 6 mice is reported ; in F), the weight of the 6 mice is reported for 0 to 18 hrs, but only that of 5 from 18 to 113 hrs (the mice infected with PAO1 WT 8.10^9 cfu were dead at 18hrs). Mann-Whitney test (A), multiple t-tests comparisons (B-C) were used (p<0.05). Error bars represent standard deviations (SD).

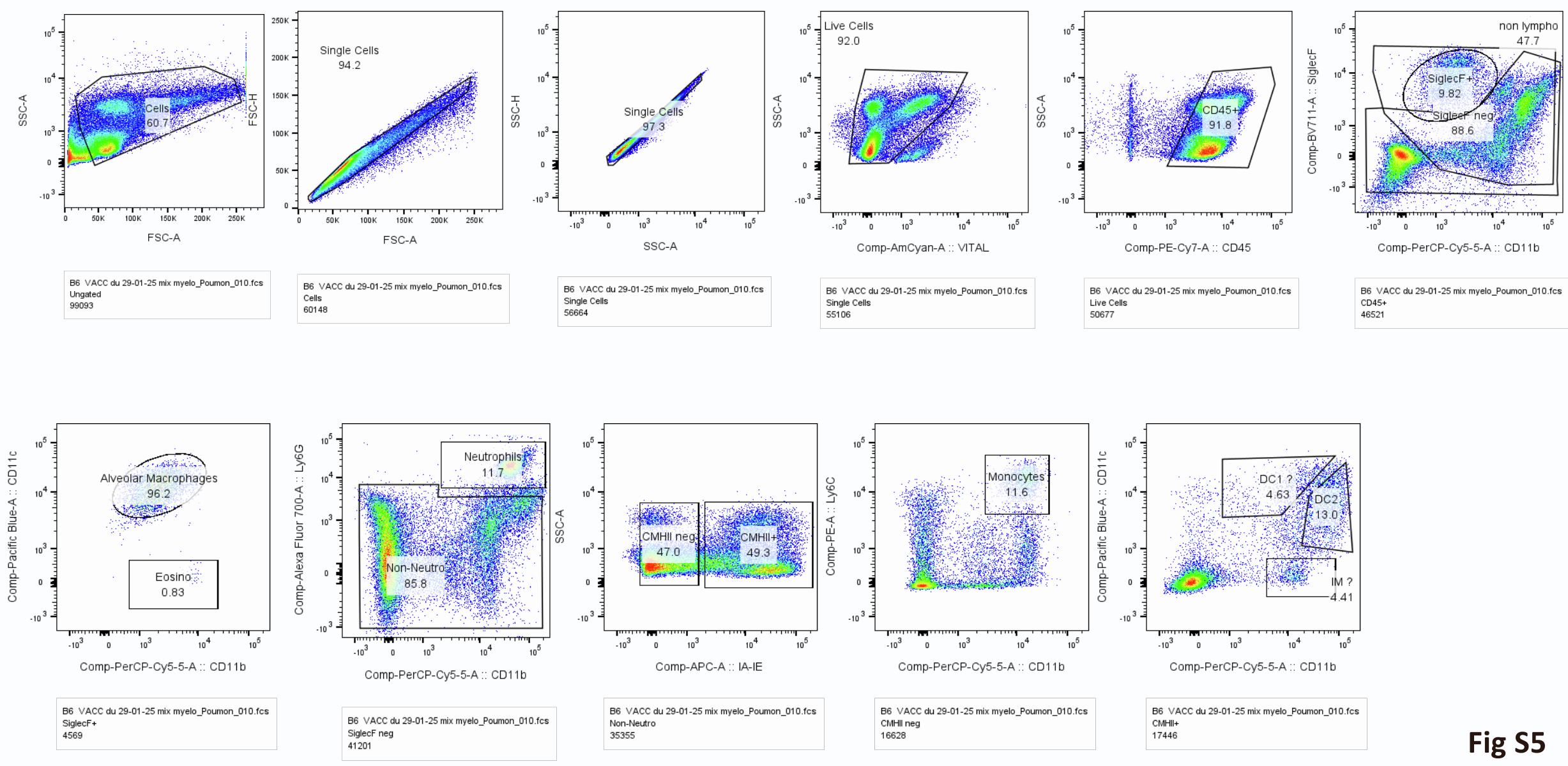


Fig S5

**Fig S5 : LUNG – MIX MYELOID GATING STRATEGY**

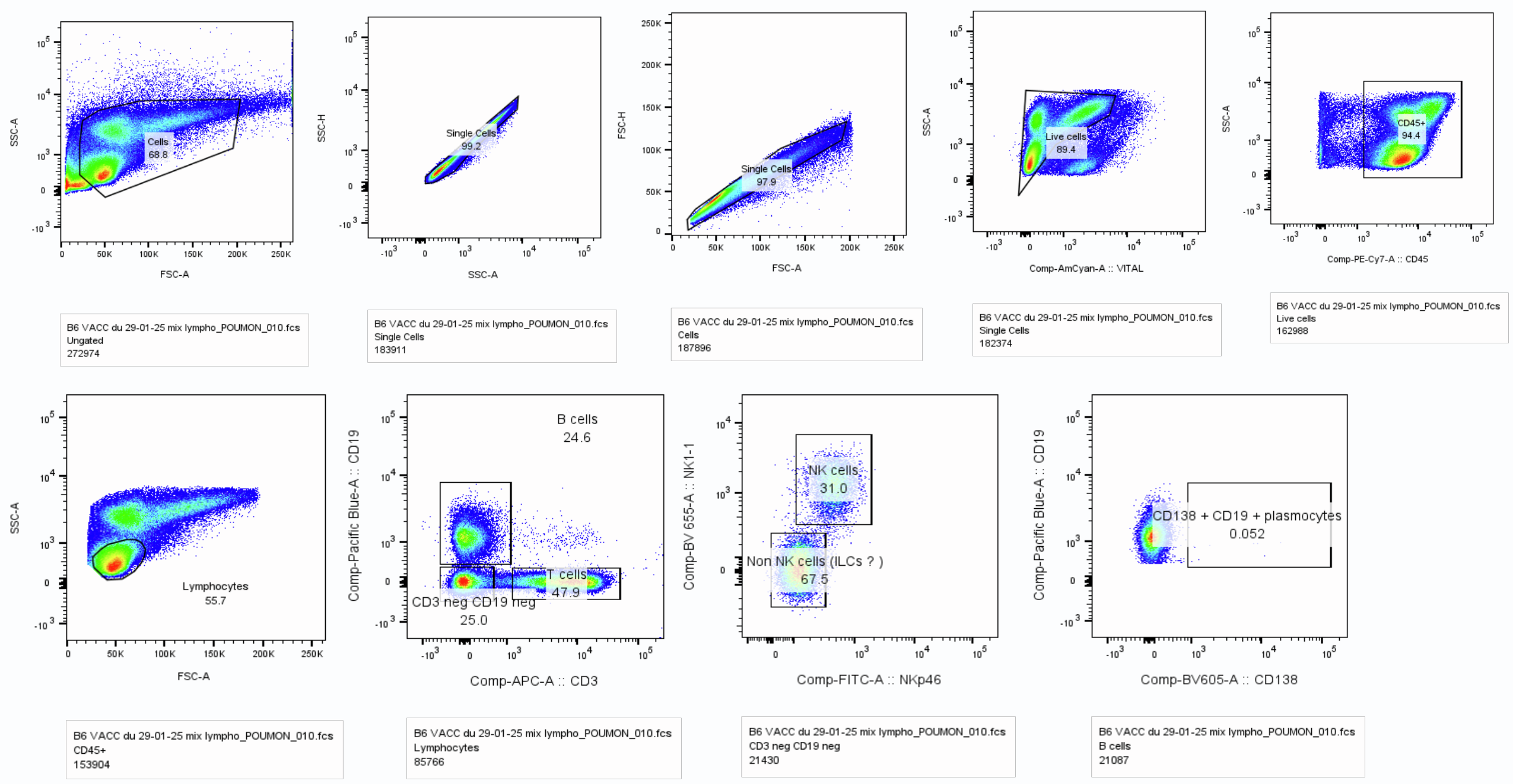


Fig S6

**Fig S6 : LUNG – Mix LYMPHOID GATING STRATEGY**

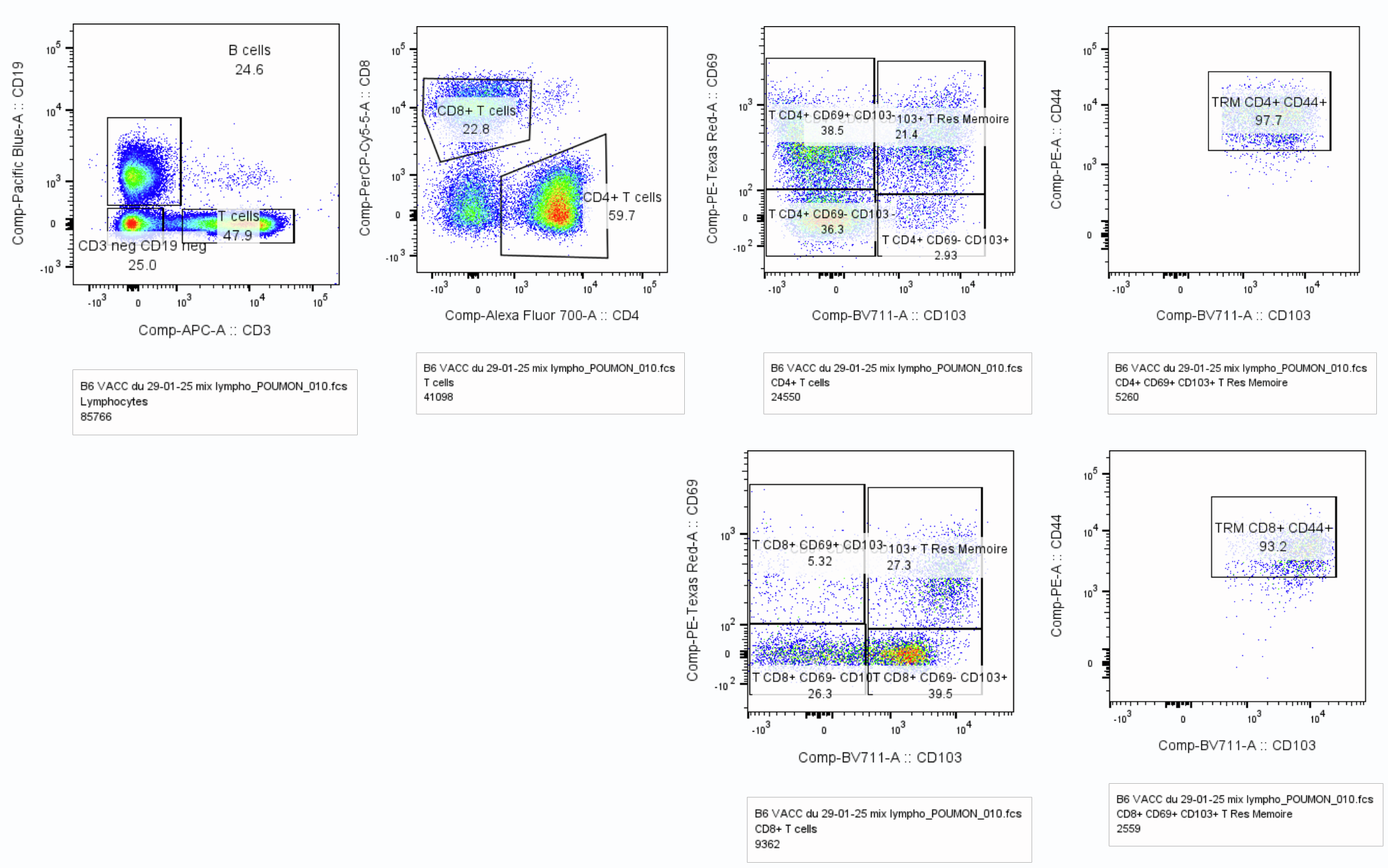


Fig S7

**Fig S7 : LUNG – Mix LYMPHOID TRM GATING STRATEGY**

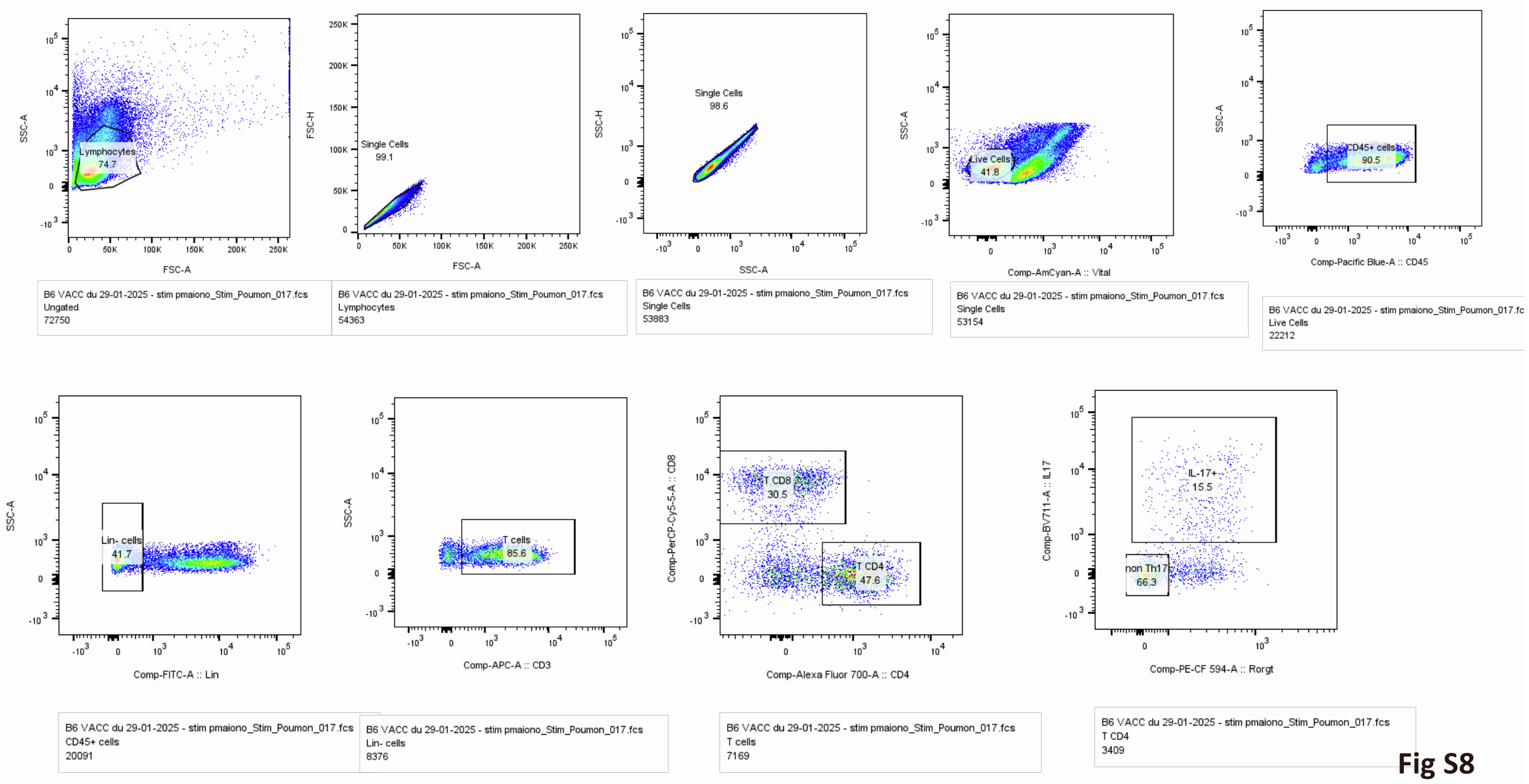


**Fig S8 : LUNG – MIX INTRA-CELL – STIM PMA/IONO**

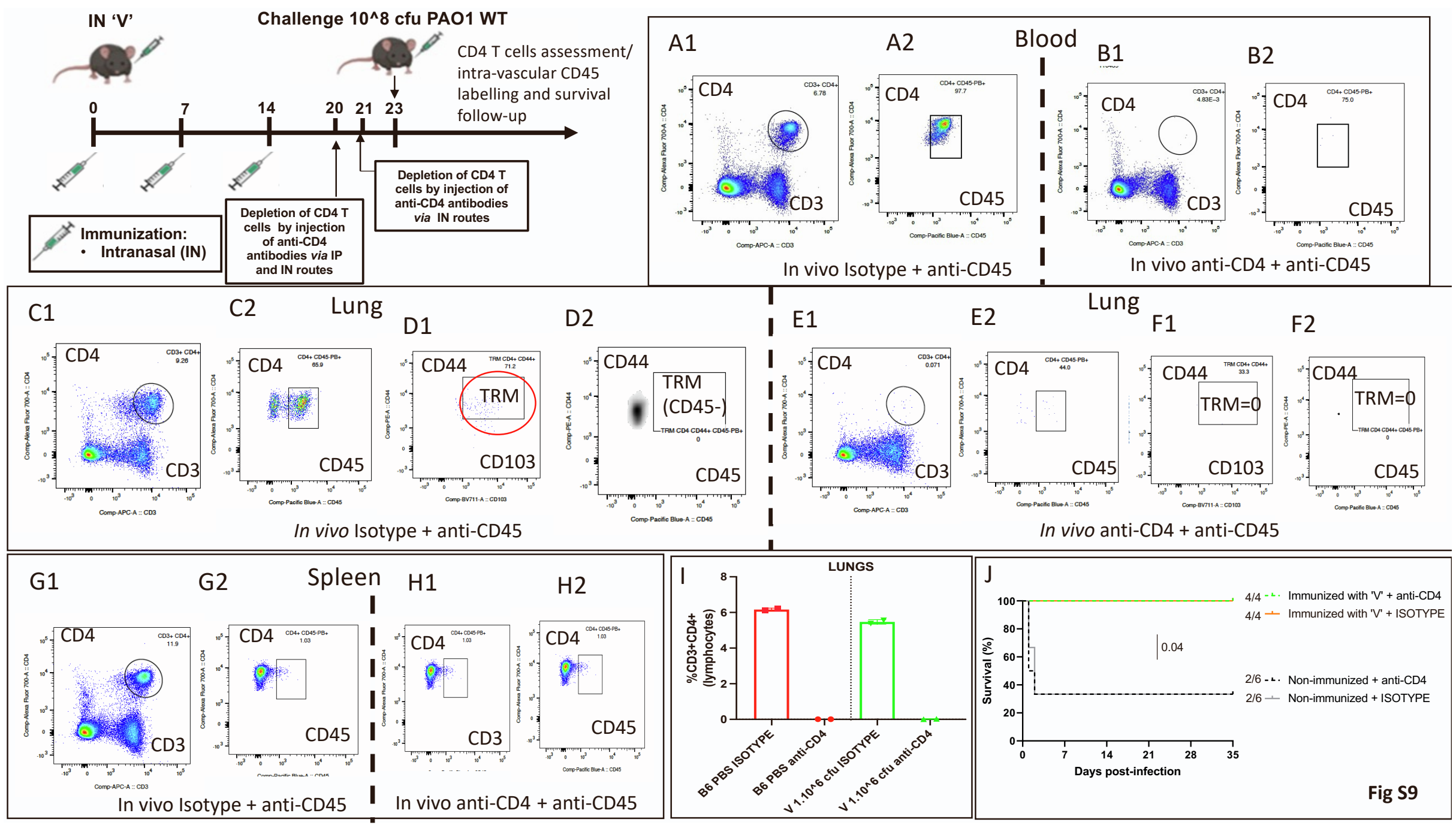


**Fig S9 : Depletion of CD4 T cells, vaccination with 'V' and challenge with PAO1-WT**

Mice were mock-vaccinated with PBS or with 'V' as in Fig 6/Fig S13 until day 14. At day 20, they were injected i.p with 250 ug of anti-CD4 antibody (or isotype) and i.n with 30 ugs of the same antibodies. At day 21, this was repeated i.n with 30 ugs of antibodies.

At day 23, 2 mice from each group were anaesthetized, injected retro-orbitally with 2 ugs of anti-CD45-PB for intra-vascular labelling. 3 min later, these mice were sacrificed, spleen and blood retrieved, as well as lungs (after perfusion) for assessment of CD4 T cells depletion and CD45 labelling (panels A-I)

The remainder mice in each group were challenged with a lethal dose of PAO1-WT (5.10^8 cfu) and survival was assessed (panel J). Survival (Log-Rank (Mantel-Cox) tests was used: * (p=0.04).

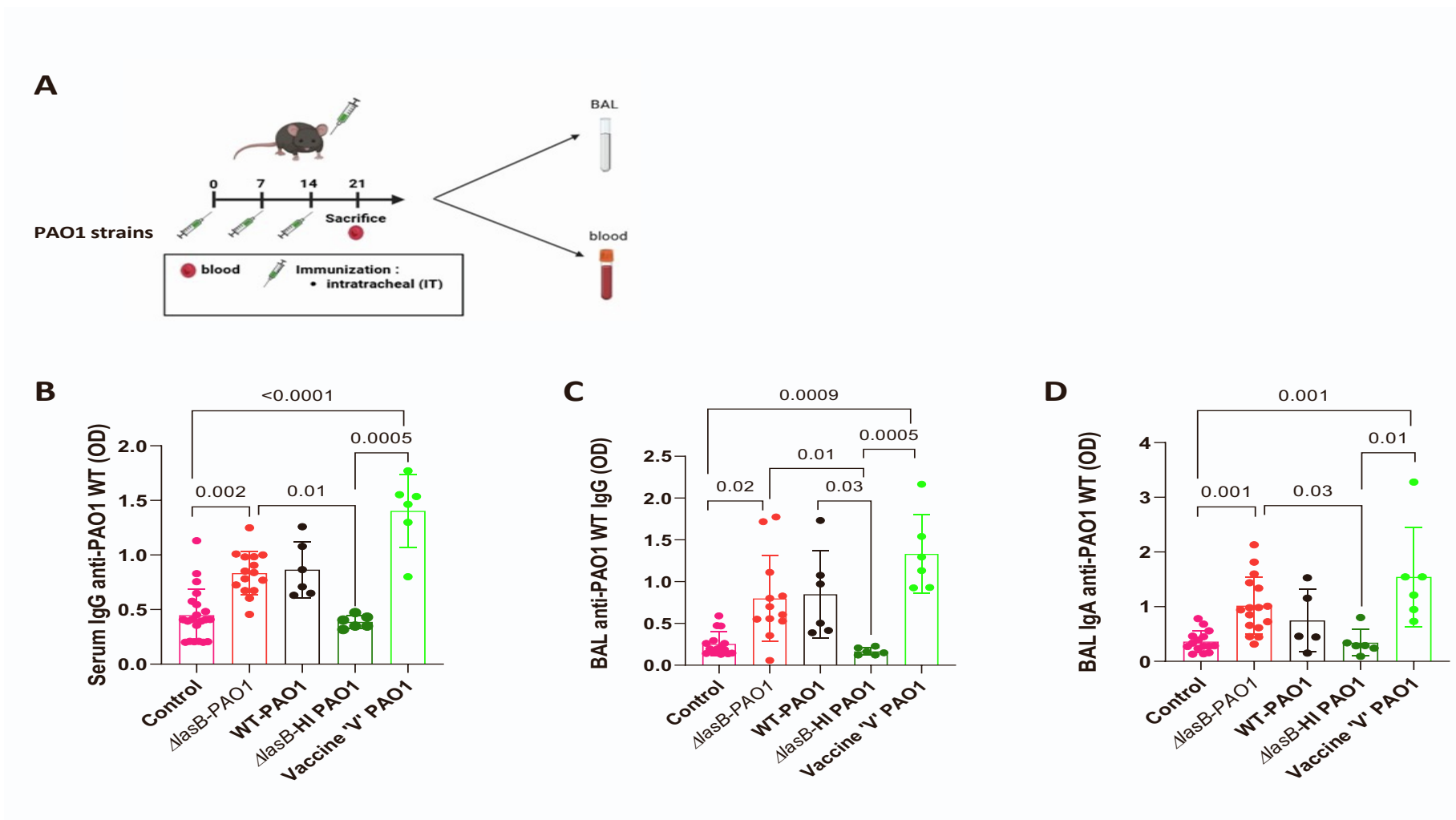


Fig S10

**Fig. S10**: **Comparative immunogenicity (humoral responses) of PAO1-WT-LB-24hrs, *ΔLasB*-PAO1-LB-24hrs, heat-inactivated (HI) *ΔLasB*-PAO1-24hrs (*ΔLasB*-PAO1-HI-24hrs), *ΔLasB*-PAO1-ASM-15days ='V'**

**A)** C57BL/6 male mice were given IT (at days 0, 7, 14) either PBS (control) or were immunized (same time points) with either 10^6CFU of live *ΔLasB*-PAO1, live PAO1-WT, *ΔLasB*-HI PAO1or live vaccine 'V'. At day 21, 7 days after the last boost, blood and BALs were collected and used for anti-PAO1-WT serum total IgG **(B)**, anti-PAO1-WT BAL total IgG **(C)**, anti-PAO1-WT BAL IgA **(D)**. Kruskal-Wallis Multiple comparison test was used for to determine significance p<0.05. Error bars represent standard deviations (SD).

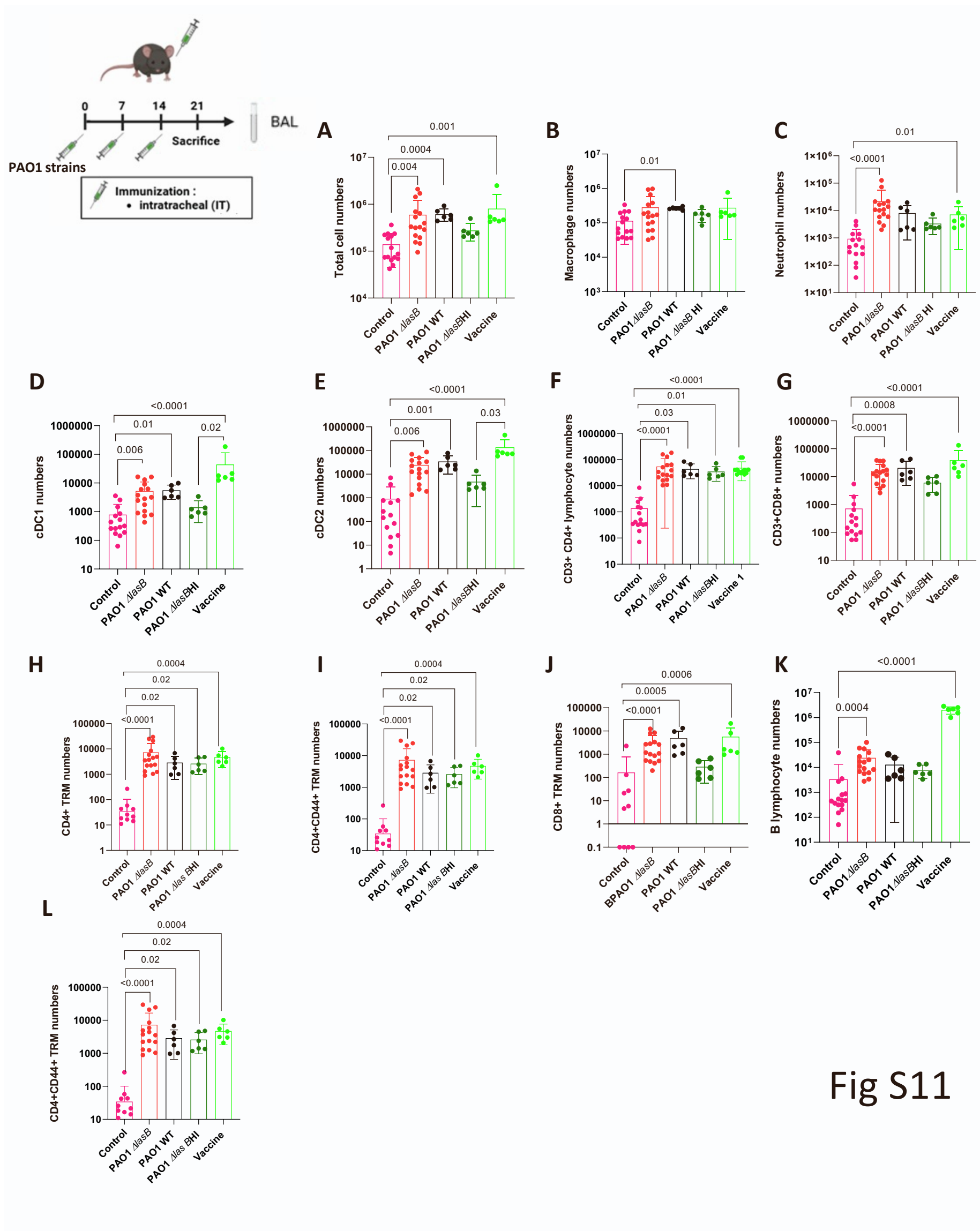


**Fig S11**: **Comparative immunogenicity (BAL cellular content) of PAO1-WT-LB-24hrs, *ΔlasB*-PAO1-LB-24hrs, heat-inactivated (HI) *ΔLasB*-PAO1-24hrs (*ΔLasB*-PAO1-HI-24hrs), *ΔLasB*-PAO1-ASM-15days ='V'**

BAL cells (see Fig S10) were recovered at d21 and analysed by FACS. Total cells, myeloid and lymphoid cells were quantified and levels plotted in panels A-L. Kruskal-Wallis Multiple comparison test was used to determine significance $p<0.05$. Error bars represent standard deviations (SD).

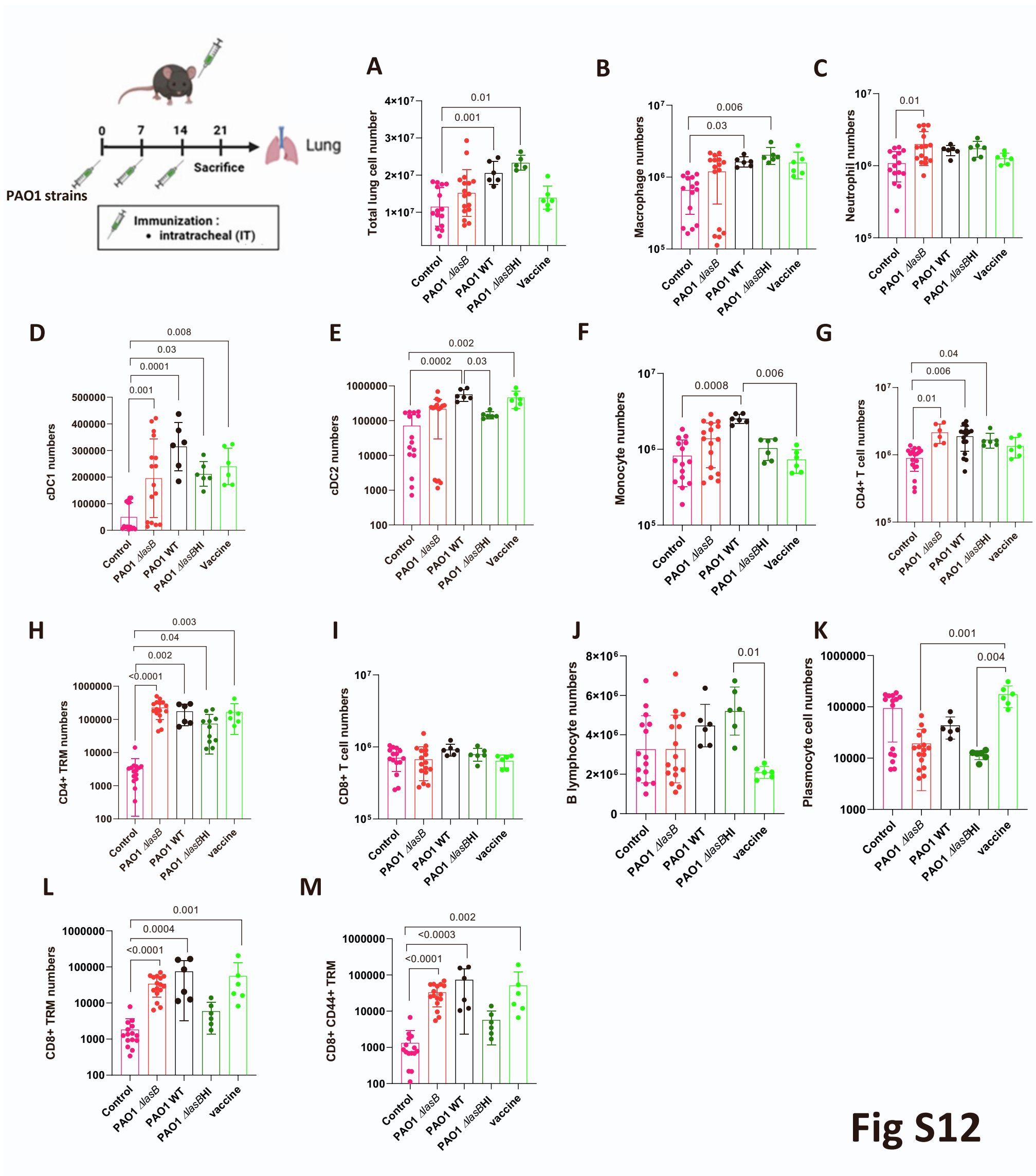


**Fig S12**: **Comparative immunogenicity (lung cellular content) of PAO1-WT-LB-24hrs, *ΔLasB*-PAO1-LB-24hrs, heat-inactivated (HI) *ΔLasB*-PAO1-24hrs (*ΔLasB*-PAO1-HI-24hrs), *ΔLasB*-PAO1-ASM-15days ='V'**

Lung cells **(see Fig S10)** were recovered at d21 and analysed by flow cytometry

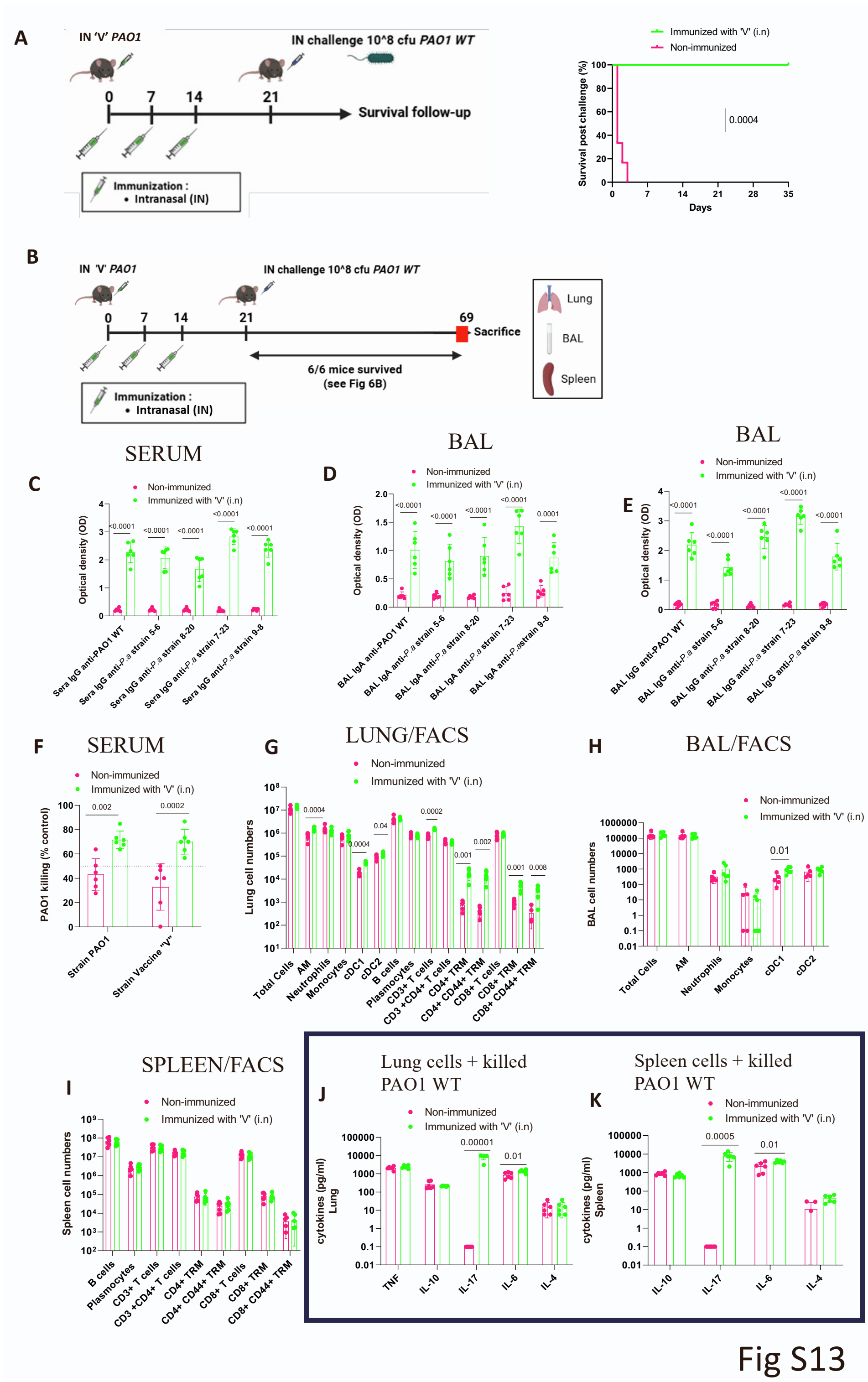


**Fig S13: Challenge and assessment of 'intermediate' memory responses following i.n immunization with 'V' and re-challenge with PAO1-WT**

**A)** Mice were immunized as above, but intra-nasally (i.n), instead of i.t. 7 days after the last boost, male C57BL/6 mice (n=6) were challenged with a lethal dose of $10^8$ CFU of PAO1-WT and survival was monitored. The p-value indicates significant differences in a Mantel-Haenszel log-rank test.

**(B)** Vaccinated mice which survived the challenge in **A)** were observed until day 69 (i.e 48 days post-challenge) when antibodies levels were measured :

(**C, D** and **E**) : serum anti-PAO1-WT (and anti-P.a clinical strains mucoid/5-6; 8-20; and non-mucoid/7-23;9-8) total IgG ; BAL anti-PAO1-WT (and anti-P.a clinical strains) total IgG; BAL anti-PAO1-WT (and anti-P.a clinical strains) IgA. **(F)** Rabbit Complement-dependent killing of PAO1-WT and 'V' (with 1/2 dilutions of sera from 'V'-immunized and non-immunized mice (n = 6)). **(G)** Day 69 FACS analysis of lung B lymphocytes, plasmocytes, alveolar macrophages (AM), neutrophils, monocytes, dendritic cells (cDC1 and cDC2), CD3+ T, CD3+CD4+ T, CD3+CD8+ T, CD4+ TRM, CD8+ TRM, CD4+CD44+TRM, CD8+CD44+ TRM, CD8+TRM and CD8+CD44+TRM T cells. NB : TRM are defined as CD3+CD69+CD103+ T cells. **H-I)** : Day 69 BAL and spleen FACS analysis of myeloid and lymphoid cells (see markers above). **J-K) :** At day 69, following the same protocol as in Fig 5 and Fig 8, lung **(J)** and spleen (**K)** were harvested and 10^6 cells were incubated with gentamycin-killed bacteria (MOI =1) during 48hrs**.** TNF, IL-10, IL17, IL-6 and IL-4 cytokines levels were then measured (ELISA) in cell supernatants . Error bars represent standard deviations. Two way ANOVA comparaison was used to determine significance $p<0.05$ for (C, D, E and F). Multiple t tests with was used for (G, H, I, K) to determine significance $p<0.05$. Error bars represent standard deviations (SD).

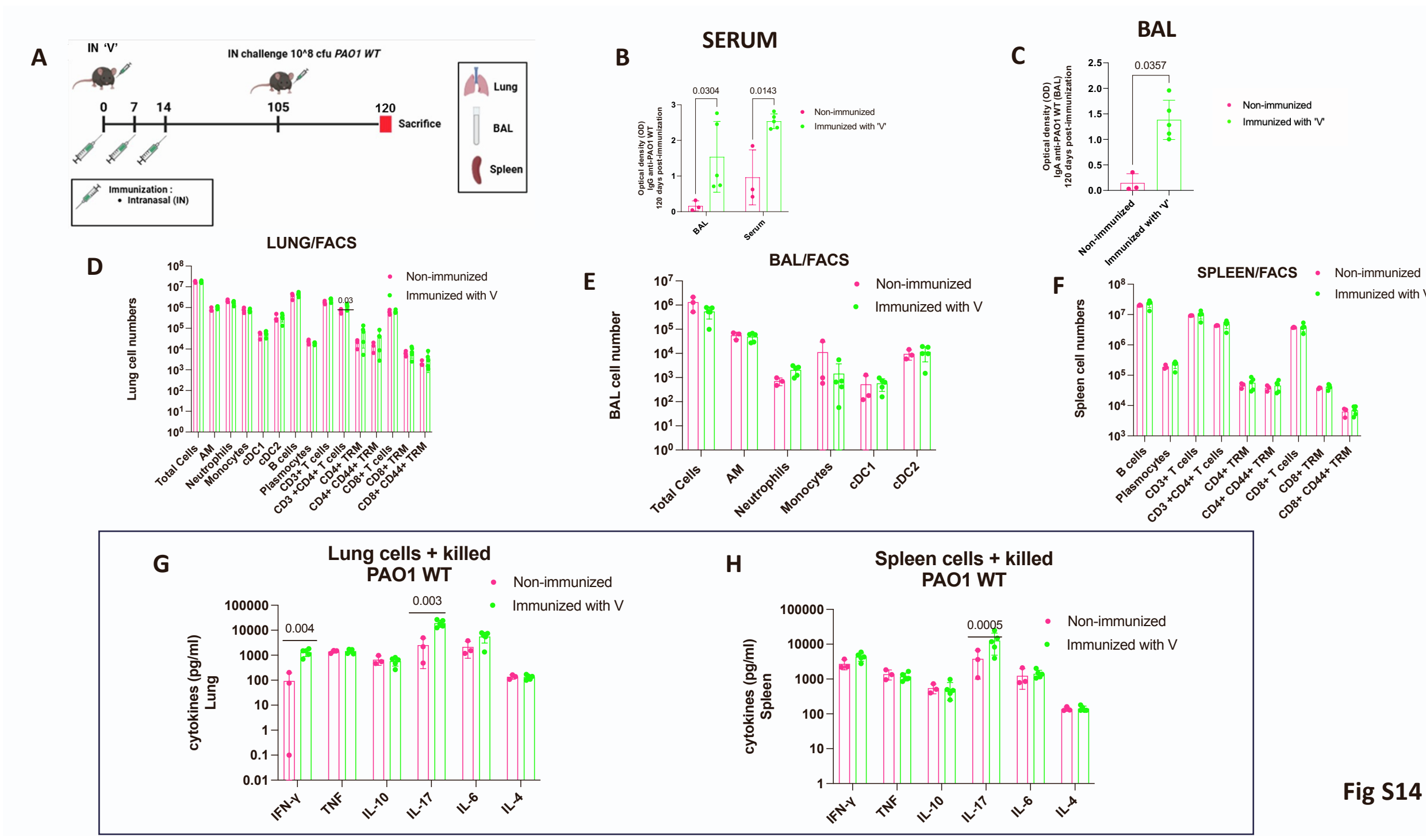


**Fig S14 : Assessment of 'long term' memory responses following i.n immunization with 'V' and re-challenge with PAO1-WT 105 days after the last boost.**

**A)** Mice (male C57BL/6, n=6) were immunized with 'V' or mock-immunised with PBS as above. 105 days after the last boost, mice were challenged with a sub-lethal dose of $10^7$ CFU of PAO1-WT. Seven days later, antibodies levels anti-PAO1 WT were measured in serum and BAL (panels B-C). Lung, BAL and spleen flow cytometry analysis of lung B lymphocytes, plasmocytes, alveolar macrophages (AM), neutrophils, monocytes, dendritic cells (cDC1 and cDC2), CD3+ T, CD3+CD4+ T, CD3+CD8+ T, CD4+ TRM, CD8+ TRM, CD4+CD44+TRM, CD8+CD44+ TRM, CD8+TRM and CD8+CD44+TRM T cells is shown in panels D-F. NB: TRM are defined as CD3+CD69+CD103+ T cells. (panels D-F). At day 120, following the same protocol as in Fig 5 and Fig 8, lung (panel G) and spleen (panel H) were harvested and 10^6 cells were incubated

with gentamycin-killed bacteria (MOI =1) during 48hrs**.** TNF, IL-10, IL17, IL-6 and IL-4 cytokines levels were then measured (ELISA) in cell supernatants. Two way ANOVA (A), Mann-Whitney test (B) and t-tests multiple comparisons were performed (C, D, E, F, G and H) ($p<0.05$). Error bars represent standard deviations (SD).

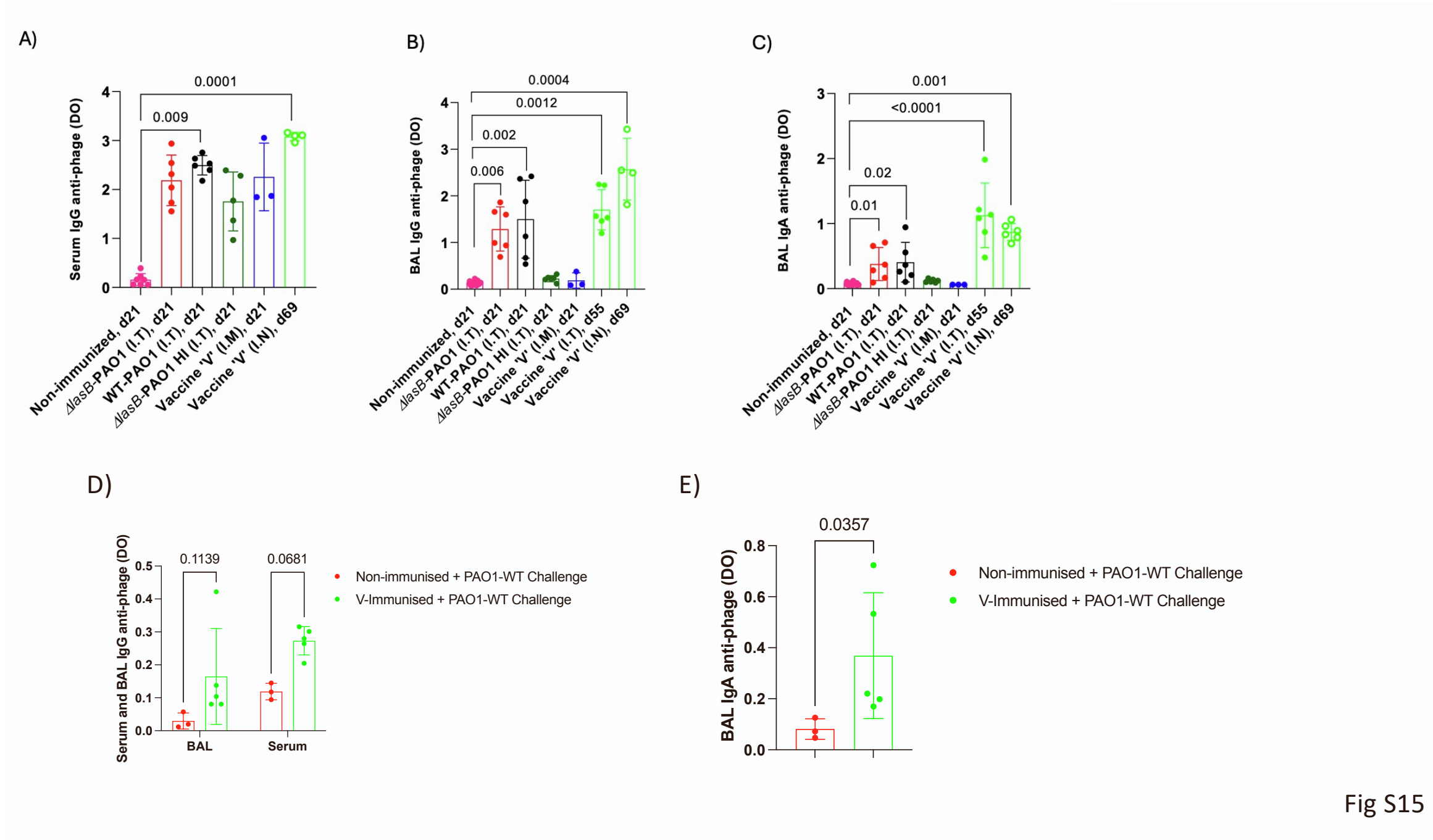


**Fig S15: Detection of anti-phage antibodies in sera and BALs from immunized mice**

**A)** C57BL/6 male were either non-immunized or immunized at days 0, 7, 14 either i.t, i.m or i.n, with either 10^6CFU of live *ΔLasB*-PAO1, live PAO1-WT, *ΔLasB*-PAO1-HI (heat-inactivated) or with the vaccine 'V' PAO1 strain. At either day 21, 55 or 69 (see corresponding Figures), blood and BALs were collected and used for assessment of anti-phage serum total IgG **(A)**, anti-phage BAL total IgG **(B)**, or anti-phage BAL IgA **(C)**. Kruskal-Wallis Multiple comparaison test was used to determine significance p<0.05. Error bars represent standard deviation.

**D-E)** C57BL/6 male were either mock-PBS-immunized or V-immunized i.n at days 0, 7, 14 with live 10^6 CFU. At day 105, all mice were further challenged with 10^7 cfu of PAO1 WT. 15 days later, mice were sacrificed, and anti-phage antibodies were assessed in sera (D) and BALs (E). Kruskal-Wallis Multiple comparaison test was used to determine significance (p<0.05). Error bars represent standard deviation.

**Supplemental references**


1. Born-Bony M, Cornu C, Villeret B, Gratio V, Voulhoux R, Sallenave JM. Intrapulmonary-administered myeloid derived suppressor cells rescue mice from Pseudomonas aeruginosa infection and promote a regulatory/repair phenotype. Mucosal Immunol. juin 2025;18(3):700‑16.

2. Holloway BW. Genetic Recombination in Pseudomonas aeruginosa. Microbiology. 1955;13(3):572‑81.

3. Kaniga K, Delor I, Cornelis GR. A wide-host-range suicide vector for improving reverse genetics in Gram-negative bacteria: inactivation of the *blaA* gene of *Yersinia enterocolitica*. Gene. 20 déc 1991;109(1):137‑41.

4. Patel H, Manning J, Ewels P, Garcia MU, Peltzer A, Hammarén R, et al. nf-core/rnaseq: nf-core/rnaseq v3.19.0 - Tungsten Turtle [Internet]. Zenodo; 2025 [cité 6 oct 2025]. Disponible sur: https://zenodo.org/records/15631172

5. Bray NL, Pimentel H, Melsted P, Pachter L. Near-optimal probabilistic RNA-seq quantification. Nat Biotechnol. mai 2016;34(5):525‑7.

6. Love MI, Huber W, Anders S. Moderated estimation of fold change and dispersion for RNA-seq data with DESeq2. Genome Biol. 5 déc 2014;15(12):550.

7. Wu W, Huang J, Duan B, Traficante DC, Hong H, Risech M, et al. Th17-stimulating protein vaccines confer protection against Pseudomonas aeruginosa pneumonia. Am J Respir Crit Care Med. 1 sept 2012;186(5):420‑7.

8. Wick RR, Judd LM, Gorrie CL, Holt KE. Completing bacterial genome assemblies with multiplex MinION sequencing. Microb Genomics. oct 2017;3(10):e000132.

9. De Coster W, D’Hert S, Schultz DT, Cruts M, Van Broeckhoven C. NanoPack: visualizing and processing long-read sequencing data. Bioinforma Oxf Engl. 1 août 2018;34(15):2666‑9.

10. Li H. Minimap2: pairwise alignment for nucleotide sequences. Bioinforma Oxf Engl. 15 sept 2018;34(18):3094‑100.

11. Kolmogorov M, Yuan J, Lin Y, Pevzner PA. Assembly of long, error-prone reads using repeat graphs. Nat Biotechnol. mai 2019;37(5):540‑6.

12. Marçais G, Delcher AL, Phillippy AM, Coston R, Salzberg SL, Zimin A. MUMmer4: A fast and versatile genome alignment system. PLoS Comput Biol. janv 2018;14(1):e1005944.

13. Robinson JT, Thorvaldsdóttir H, Winckler W, Guttman M, Lander ES, Getz G, et al. Integrative Genomics Viewer. Nat Biotechnol. janv 2011;29(1):24‑6.

14. Tatusova T, DiCuccio M, Badretdin A, Chetvernin V, Nawrocki EP, Zaslavsky L, et al. NCBI prokaryotic genome annotation pipeline. Nucleic Acids Res. 19 août 2016;44(14):6614‑24.

15. Sereme Y, Schrimp C, Faury H, Agapoff M, Lefebvre-Wloszczowski E, Chang Marchand Y, et al. A live attenuated vaccine to prevent severe neonatal Escherichia coli K1 infections. Nat Commun. 8 avr 2024;15(1):3021.

16. O'Loughlin CT, Miller LC, Siryaporn A, Drescher K, Semmelhack MF, Bassler BL. A quorum-sensing inhibitor blocks Pseudomonas aeruginosa virulence and biofilm formation. Proc Natl Acad Sci U S A . 2013 Oct 29;110(44):17981-6.

17. Schlitzer A, Sivakamasundari V, Chen J, Sumatoh HRB, Schreuder J, Lum J, et al. Identification of cDC1- and cDC2-committed DC progenitors reveals early lineage priming at the common DC progenitor stage in the bone marrow. Nat Immunol. juill 2015;16(7):718‑28.

18. Fejer G, Wegner MD, Györy I, Cohen I, Engelhard P, Voronov E, et al. Nontransformed, GM-CSF-dependent macrophage lines are a unique model to study tissue macrophage functions. Proc Natl Acad Sci U S A. 11 juin 2013;110(24):E2191-2198.

19. Ramsay PL, Luo Z, Magdaleno SM, Whitbourne SK, Cao X, Park MS, et al. Transcriptional regulation of CCSP by interferon-γ in vitro and in vivo. Am J Physiol-Lung Cell Mol Physiol. 1 janv 2003;284(1):L108‑18.

20. Granoff DM. Relative importance of complement-mediated bactericidal and opsonic activity for protection against meningococcal disease. Vaccine. 24 juin 2009;27:B117‑25.

21. Sweere JM, Van Belleghem JD, Ishak H, Bach MS, Popescu M, Sunkari V, et al. Bacteriophage trigger antiviral immunity and prevent clearance of bacterial infection. Science. 29 mars 2019;363(6434):eaat9691.

22. Toder DS, Ferrell SJ, Nezezon JL, Rust L, Iglewski BH. lasA and lasB genes of Pseudomonas aeruginosa: analysis of transcription and gene product activity. Infect Immun. avr 1994;62(4):1320-7.

23. Llanes C, Pourcel C, Richardot C, Plésiat P, Fichant G, Cavallo JD, et al. Diversity of β-lactam resistance mechanisms in cystic fibrosis isolates of Pseudomonas aeruginosa: a French multicentre study. J Antimicrob Chemother. 1 août 2013;68(8):1763-71.
